# Supplementary material for: Accessing gold p-acid reactivity under electrochemical anode oxidation (EAO) through oxidation relay
Source: Nat Commun. 2023 Dec 13;14:8265. doi: 10.1038/s41467-023-44025-0 (PMC10719393; doi:10.1038/s41467-023-44025-0)
Supplement: Supplementary file 1 — Supplementary Information [file 41467_2023_44025_MOESM1_ESM.pdf]

Supplementary Information for Accessing Gold p-Acid Reactivity under Electrochemical Anode Oxidation (EAO) through Oxidation Relay

Shuyao Zhang, Jingwen Wei, Xiaohan Ye, Angel Perez & Xiaodong Shi

Supplementary Methods S2

1. General Methods and Materials S2
2. General Procedures S2
3. Condition Optimization and Substrate Scope Screening S5
4. Compounds Characterization S7
5. NMR Spectra Data S18

1. General Methods and Materials

All of the reactions dealing with air and/or moisture-sensitive compounds were carried out under an atmosphere of argon using oven/flame-dried glassware and standard syringe/septa techniques. Unless otherwise noted, all commercial reagents and solvents were obtained from the commercial provider and used without further purification. ^1H NMR and ^{13}C NMR spectra were recorded on Agilent 400/600 MHz spectrometers spectrometers. Chemical shifts were reported relative to internal tetramethylsilane (δ 0.00 ppm) or $CDCl_3$ (δ 7.26 ppm) for ^1H and $CDCl_3$ (δ 77.00 ppm) for ^{13}C. Flash column chromatography was performed on 230-430 mesh silica gel. Analytical thin layer chromatography was performed with precoated glass baked plates (250μ) and visualized by fluorescence and by charring after treatment with potassium permanganate stain.

2. General Procedures

2.0 General procedure for the ElectraSyn Set-up

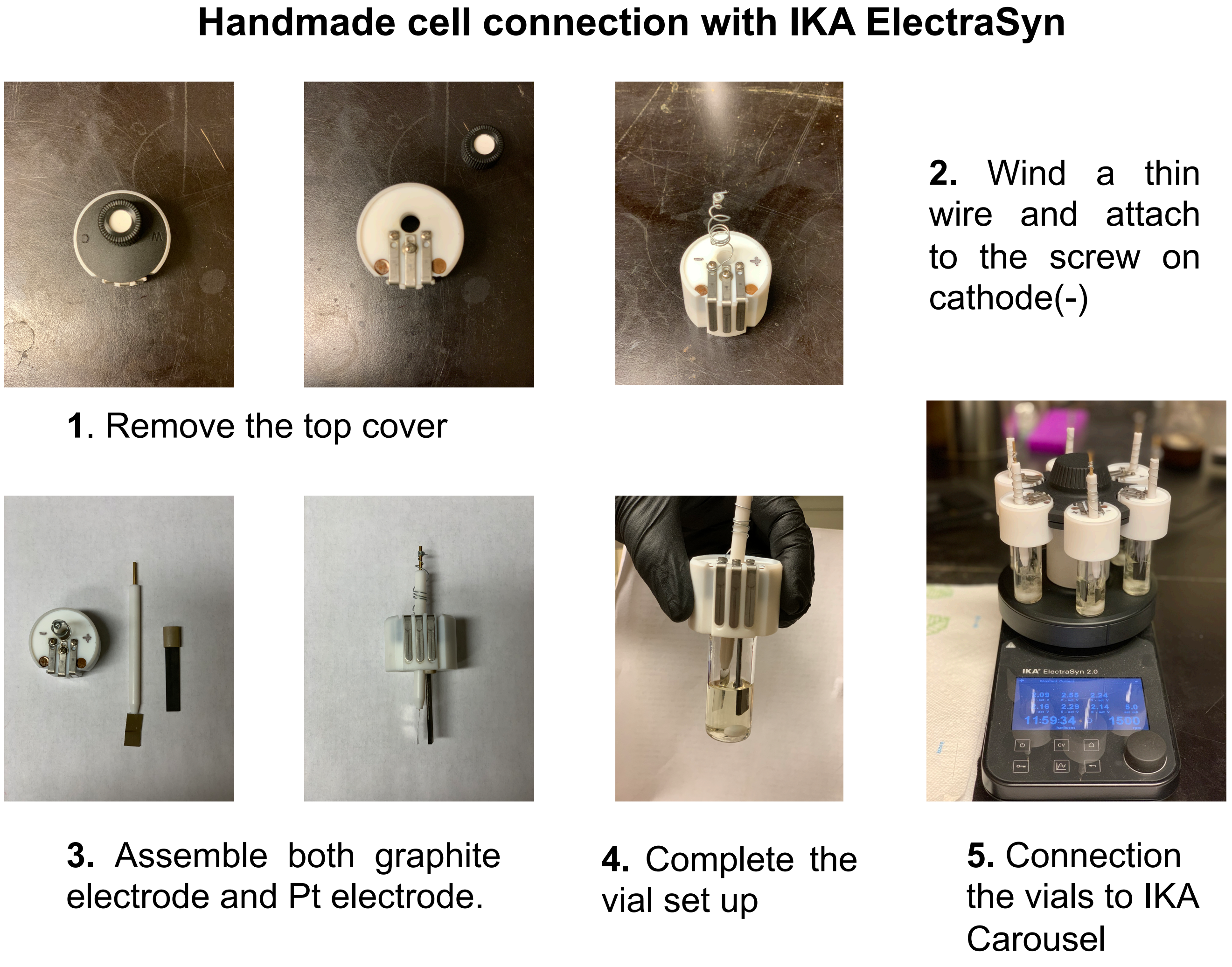

Supplementary Fig. 1. ElectraSyn Set-up

2.1 General procedure for EAO promoted alkene/alkyne π-activation reaction

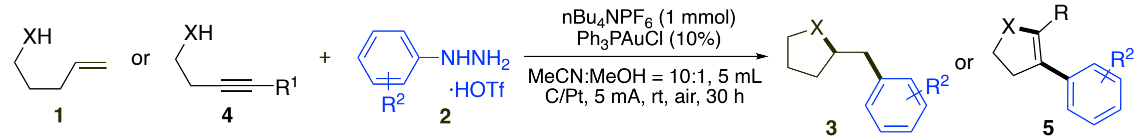

Supplementary Fig. 2. General procedure for EAO promoted alkene/alkyne π-activation reaction

To a 10 mL ElectraSyn screwed vial with 387 mg nBu$_4$NPF$_6$ (1 mmol) in MeCN:MeOH = 10:1 (5.5 mL), alkene **1** or alkyne 4 (0.5 mmol, 1.0 equiv.), Ph$_3$PAuCl (0.05 mmol, 10 mol%) and the first batch of aryl hydrazine HOTf salt **2** (0.5 mmol, 1.0 equiv) was added. The vial was placed on IKA Carousel and run under constant current at 5 mA for 10 h. After the time is over, the cap was opened and another 1 eq hydrazine was added, then the reaction was performed under same condition for another 10 h. This step was repeated twice until all 3 eq hydrazine HOTf salt **2** was consumed. the solvent was removed under reduced pressure and the residue was purified by flash chromatography on silica gel to give desired product **3** or **5**.

2.2 General procedure for the synthesis of different internal alkyne 4

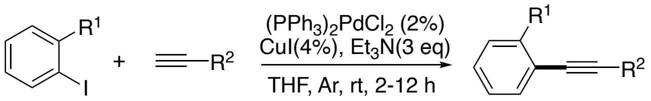

Supplementary Fig. 3. General procedure for the synthesis of different internal alkyne **4**

All alkyne substrates **4** were synthesized with literature reported Sonogashira coupling strategy. To a round bottom flask was added iodobenzene substrate (5 mmol, 1 eq), (PPh$_3$)$_2$PdCl$_2$ (0.1 mmol, 2%) and CuI (0.2 mmol, 4%). The flask was capped

and flushed with Ar. THF (25 mL, 0.2 M), Et_3N (15 mmol, 3 eq) and terminal alkyne (6 mmol, 1.2 eq) were added in sequence. The reaction was stirred under rt for 2-12 h (monitored by TLC). Once the reaction finished, the mixture was filtrated and washed with EA. The solvent was removed under reduced pressure and the residue was purified by flash chromatography on silica gel to get the desired product **4**.

3. Condition Optimization and Substrate Scope Screening

3.1 Optimization studies of EAO promoted alkene/alkyne π-activation reaction

Supplementary Table 1 | Reaction optimization

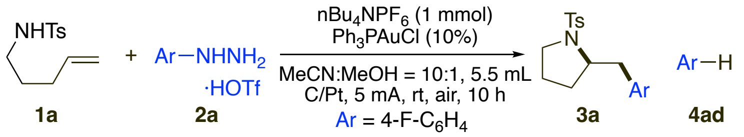

Entry	Condition Variation	Yield		Note
		3a	4ad	
1	none	32%	29%	-
2	MeCN 5 mL only	trace	19%	Gold decomposition
3	MeCN:MeOH = 4:1, 5 mL	16%	30%	-
4	MeCN:MeOH = 1:1, 5 mL	12%	26%	-
5	MeCN:MeOH =1:4, 5 mL	6%	32%	-
6	Only MeOH	trace	24%	-
7	$Ph_3PAu(TA\text{-}Me)OTf$ instead	12%	46%	-
8	JohnPhosAuCl instead	0%	43%	Gold decomposition
9	MeDalPhosAuCl instead	trace	37%	Gold decomposition
10	$Ph_2P(N_1Bn)BTAAuCl$ instead	7%	44%	-
11	$dppm(AuBr)_2$ instead	trace	41%	Gold decomposition
12	$IPrAuNTf_2$ instead	0%	48%	Gold decomposition
13	$(2\text{-}MePh)_3PAuCl$ instead	trace	36%	Gold decomposition
14	nBu_4NBF_4 (1 mmol)	15%	31%	
15	$LiClO_4$ (1 mmol)	37%	32%	-
16	nBu_4NPF_6 (0.5 mmol)	38%	37%	-
17	nBu_4NPF_6 (1.5 mmol)	35%	40%	-
18	nBu_4NPF_6 (1 mmol) + $LiClO_4$ (0.5 mmol)	35%	41%	-
19	EtOH instead of MeOH	20%	27%	-
20	iPrOH instead of MeOH	17%	28%	-
21	tBuOH instead of MeOH	10%	22%	-
22	HFIP instead of MeOH	4%	9%	Gold decomposition
23	CF_3CH_2OH instead of MeOH	0%	6%	Gold decomposition
24	H_2O instead of MeOH	6%	33%	-
25	DCM instead of MeOH	0%	48%	Gold decomposition
26	2 eq alkene instead	33%	25%	-
27	45 °C	trace	44%	Gold decomposition
28	45 °C, O_2	5%	14%	Gold decomposition
29	rt, N_2	6%	43%	Gold decomposition
30	rt, O_2	24%	9%	-
31	3 mA, 15 h	32%	27%	-

32	2 mA, 25 h	35%	23%	-
33	10 mA, 5 h	9%	37%	Gold decomposition
34	double alkene and hydrazine (20 h)	34%	35%	-
35	Double alkene, hydrazine and gold (20 h)	54%	22%	-
36	0.5 mmol alkene, 1.5 mmol hydrazine, 0.05 mmol gold (30 h)	70%	21%	-
37	0.5 mmol alkene, 1.5 mmol hydrazine, 0.05 mmol gold, 0.5 mmol nBu_4NPF_6 (30 h)	70%	23%	-
**38**	**0.5 mmol alkene, 1.5 mmol hydrazine, 0.05 mmol gold (add 0.5 mmol hydrazine, run 10 h, repeat 3 times)**	**92%**	**18%**	**-**

3.2 Substrate scope of failed alkenes/hydrazines

We tested various different alkene and hydrazine substrates. Attached is a list of substrates that is not reactive or will decompose under optimized conditions.

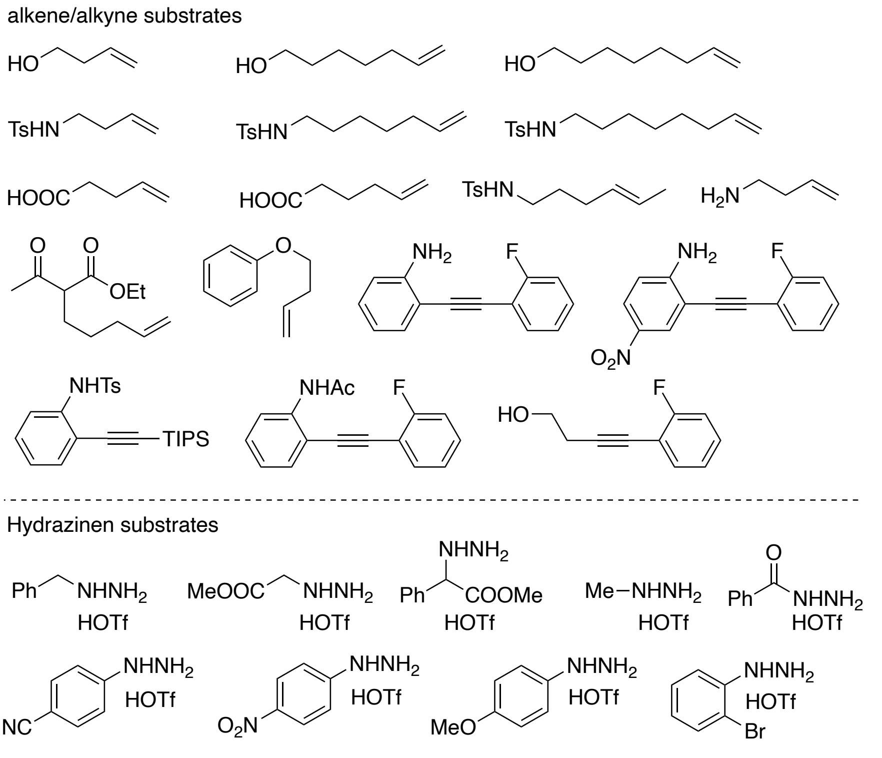

Supplementary Fig. 4. Substrate scope of failed alkenes/hydrazines

4. Compound Characterization

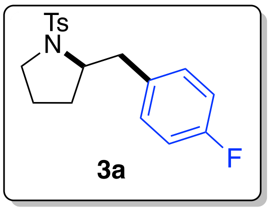

3a

2-(4-fluorobenzyl)-1-tosylpyrrolidine
3a was prepared following the general procedure 2.1 and purified by column chromatography (hexane:EA = 5:1) as a white solid.
^{1}H NMR (600 MHz, Chloroform-*d*) δ 7.75 (d, *J* = 8.3 Hz, 2H), 7.35 – 7.29 (m, 2H), 7.21 (dd, *J* = 8.5, 5.5 Hz, 2H), 6.98 (t, *J* = 8.7 Hz, 2H), 3.83 – 3.75 (m, 1H), 3.36 (dddd, *J* = 10.1, 5.4, 4.2, 2.7 Hz, 1H), 3.20 – 3.09 (m, 2H), 2.79 (dd, *J* = 13.5, 9.1 Hz, 1H), 2.43 (s, 3H), 1.64 – 1.58 (m, 2H), 1.48 – 1.41 (m, 2H).
^{13}C NMR (151 MHz, $CDCl_3$) δ 161.71(d, *J* = 244.7 Hz), 143.40, 134.64, 134.06, 131.07 (d, *J* = 7.9 Hz), 129.70, 127.52, 115.21 (d, *J* = 21.1 Hz), 61.44, 49.26, 41.76, 29.86, 23.81, 21.53.
^{19}F NMR (564 MHz, Chloroform-*d*) δ -116.75.
HRMS m/z (ESI) calcd. for $C_{18}H_{21}FNO_2S^+$ $(M+H)^+$ 334.1272, found 334.1315.

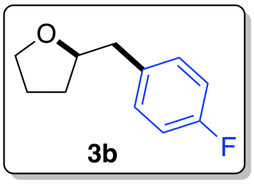

3b

2-(4-fluorobenzyl)tetrahydrofuran
3b was prepared following the general procedure 2.1 and purified by column chromatography (hexane:EA = 20:1) as a colorless oil.
^{1}H NMR (600 MHz, Chloroform-*d*) δ 7.21 – 7.16 (m, 2H), 6.97 (t, *J* = 8.7 Hz, 1H), 4.03 (dq, *J* = 7.7, 6.3 Hz, 1H), 3.88 (ddd, *J* = 8.3, 7.1, 6.2 Hz, 1H), 3.74 (td, *J* = 8.0, 6.3 Hz, 1H), 2.86 (dd, *J* = 13.8, 6.7 Hz, 1H), 2.74 (dd, *J* = 13.8, 6.0 Hz, 1H), 1.93 (dddd, *J* = 11.2, 8.2, 6.2, 4.8 Hz, 1H), 1.90 – 1.81 (m, 2H), 1.56 – 1.51 (m, 1H).
^{13}C NMR (151 MHz, Chloroform-*d*) δ 161.53 (d, *J* = 243.5 Hz), 134.68, 130.59 (d, *J* = 7.8 Hz), 115.08 (d, *J* = 21.0 Hz), 79.95, 68.00, 41.04, 30.96, 25.63.
^{19}F NMR (564 MHz, Chloroform-*d*) δ -117.40.
HRMS m/z (ESI) calcd. for $C_{11}H_{14}FO^+$ $(M+H)^+$ 181.1023, found 181.1051.

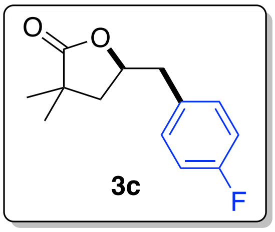

3c

5-(4-fluorobenzyl)-3,3-dimethyldihydrofuran-2(3*H*)-one
3c was prepared following the general procedure 2.1 and purified by column chromatography (hexane:EA = 20:1) as a colorless oil.
^{1}H NMR (600 MHz, Chloroform-*d*) δ 7.20 (dd, *J* = 8.5, 5.5 Hz, 2H), 7.01 (t, *J* = 8.7 Hz, 2H), 4.60 (ddt, *J* = 10.0, 6.8, 5.8 Hz, 1H), 3.04 (dd, *J* = 14.2, 6.8 Hz, 1H), 2.88 (dd, *J* = 14.2, 5.8 Hz, 1H), 2.09 (dd, *J* = 12.7, 5.8 Hz, 1H), 1.80 (dd, *J* = 12.7, 10.0 Hz, 1H), 1.24 (d, *J* = 2.2 Hz, 6H).
^{13}C NMR (151 MHz, Chloroform-*d*) δ 181.68, 161.93 (d, *J* = 245.5 Hz), 131.96, 130.89 (d, *J* = 8.0 Hz), 115.46 (d, *J* = 21.3 Hz), 42.83, 40.75, 40.44, 24.97, 24.41.
^{19}F NMR (564 MHz, Chloroform-*d*) δ -115.94.
HRMS m/z (ESI) calcd. for $C_{13}H_{16}FO_2^+$ $(M+H)^+$ 223.1129, found 223.1158.

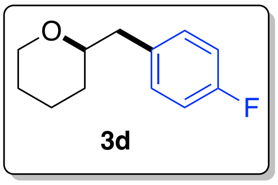

3d

2-(4-fluorobenzyl)tetrahydro-2*H*-pyran

3d was prepared following the general procedure 2.1 and purified by column chromatography (hexane:EA = 20:1) as a colorless oil.

^{1}H NMR (600 MHz, Chloroform-*d*) δ 7.16 (dd, *J* = 8.4, 5.6 Hz, 2H), 6.96 (t, *J* = 8.7 Hz, 2H), 4.01 – 3.93 (m, 1H), 3.48 – 3.36 (m, 2H), 2.82 (dd, *J* = 13.9, 7.0 Hz, 1H), 2.62 (dd, *J* = 13.9, 6.1 Hz, 1H), 1.81 (dq, *J* = 11.7, 2.5 Hz, 1H), 1.56 (td, *J* = 13.6, 12.6, 7.2 Hz, 2H), 1.51 – 1.40 (m, 2H), 1.28 (tdd, *J* = 12.8, 10.8, 5.0 Hz, 1H).

^{13}C NMR (151 MHz, $CDCl_3$) δ 161.48 (d, *J* = 242.7 Hz), 134.50, 130.71 (d, *J* = 7.9 Hz), 114.97 (d, *J* = 20.8 Hz), 78.71, 68.67, 42.29, 31.43, 26.01, 23.48.

^{19}F NMR (564 MHz, Chloroform-*d*) δ -117.45.

HRMS m/z (ESI) calcd. for $C_{12}H_{16}FO^+$ $(M+H)^+$ 195.1180, found 195.1217.

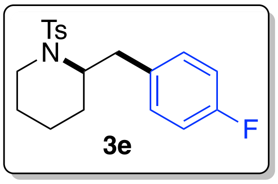

2-(4-fluorobenzyl)-1-tosylpiperidine

3e was prepared following the general procedure 2.1 and purified by column chromatography (hexane:EA = 5:1) as a pale yellow solid.

^{1}H NMR (600 MHz, Chloroform-*d*) δ 7.59 (d, *J* = 8.1 Hz, 2H), 7.22 (d, *J* = 8.0 Hz, 2H), 7.14 – 7.04 (m, 2H), 6.94 (t, *J* = 8.7 Hz, 2H), 4.30 – 4.19 (m, 1H), 3.84 – 3.72 (m, 1H), 3.07 (td, *J* = 13.4, 2.5 Hz, 1H), 2.92 – 2.75 (m, 2H), 2.40 (s, 3H), 1.60 (qd, *J* = 16.8, 13.0 Hz, 3H), 1.53 – 1.35 (m, 3H).

^{13}C NMR (151 MHz, $CDCl_3$) δ 161.60 (d, *J* = 244.4 Hz), 142.90, 138.33, 134.28, 130.55 (d, *J* = 7.8 Hz), 129.59, 126.95, 115.31 (d, *J* = 21.0 Hz), 54.44, 40.88, 35.03, 26.16, 24.83, 21.50, 18.35.

^{19}F NMR (564 MHz, Chloroform-*d*) δ -116.66.

HRMS m/z (ESI) calcd. for $C_{19}H_{23}FNO_2S^+$ $(M+H)^+$ 348.1428, found 348.1439.

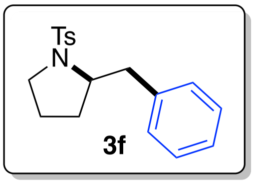

2-benzyl-1-tosylpyrrolidine

3f was prepared following the general procedure 2.1 and purified by column chromatography (hexane:EA = 5:1) as a colorless oil.

^{1}H NMR (600 MHz, Chloroform-*d*) δ 7.76 (d, *J* = 8.2 Hz, 2H), 7.31 (dd, *J* = 11.7, 7.7 Hz, 4H), 7.23 (dd, *J* = 16.0, 7.3 Hz, 3H), 3.82 (ddt, *J* = 9.7, 7.7, 3.4 Hz, 1H), 3.48 – 3.33 (m, 1H), 3.25 (dd, *J* = 13.3, 3.5 Hz, 1H), 3.13 (dt, *J* = 10.1, 7.6 Hz, 1H), 2.75 (dd, *J* = 13.3, 9.7 Hz, 1H), 2.43 (s, 3H), 1.70 – 1.59 (m, 2H), 1.54 – 1.36 (m, 2H).

^{13}C NMR (151 MHz, $CDCl_3$) δ 143.35, 138.53, 134.63, 129.69, 129.66, 128.45, 127.54, 126.43, 61.63, 49.28, 42.75, 29.85, 23.81, 21.56.

HRMS m/z (ESI) calcd. for $C_{18}H_{22}NO_2S^+$ $(M+H)^+$ 316.1366, found 316.1412.

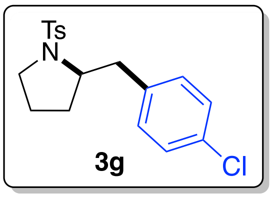

2-(4-chlorobenzyl)-1-tosylpyrrolidine

3g was prepared following the general procedure 2.1 and purified by column chromatography (hexane:EA = 5:1) as a pale yellow oil.

^{1}H NMR (600 MHz, Chloroform-*d*) δ 7.74 (d, *J* = 8.3 Hz, 2H), 7.32 (d, *J* = 8.0 Hz, 2H), 7.29 – 7.26 (m, 2H), 7.19 (d, *J* = 8.4 Hz, 2H), 3.78 (dh, *J* = 11.4, 3.5, 2.7 Hz, 1H), 3.36 (ddd, *J* = 10.4, 6.2, 3.8 Hz, 1H), 3.20 – 3.08 (m, 2H), 2.79 (dd, *J* = 13.4, 9.1 Hz, 1H), 2.43 (s, 3H), 1.63 – 1.55 (m, 2H), 1.50 – 1.40 (m, 2H).

^{13}C NMR (151 MHz, $CDCl_3$) δ 143.47, 136.84, 134.48, 132.32, 131.04, 129.73, 128.55, 127.52, 61.30, 49.27, 41.96, 29.88, 23.84, 21.57.

HRMS m/z (ESI) calcd. for $C_{18}H_{21}ClNO_2S^+$ $(M+H)^+$ 350.0976, found 350.1001.

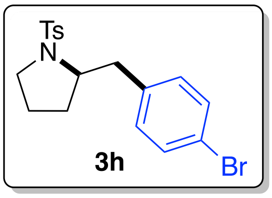

2-(4-bromobenzyl)-1-tosylpyrrolidine

3h was prepared following the general procedure 2.1 and purified by column chromatography (hexane:EA = 5:1) as a white solid.

^{1}H NMR (600 MHz, Chloroform-*d*) δ 7.74 (d, *J* = 8.0 Hz, 2H), 7.42 (d, *J* = 8.3 Hz, 2H), 7.32 (d, *J* = 8.0 Hz, 2H), 7.13 (d, *J* = 8.1 Hz, 2H), 3.78 (td, *J* = 8.2, 6.8, 3.7 Hz, 1H), 3.36 (ddd, *J* = 10.4, 6.3, 3.8 Hz, 1H), 3.13 (tt, *J* = 14.2, 5.5 Hz, 2H), 2.78 (dd, *J* = 13.4, 9.1 Hz, 1H), 2.43 (s, 3H), 1.70 – 1.54 (m, 2H), 1.45 (ddt, *J* = 11.9, 9.3, 5.5 Hz, 2H).

^{13}C NMR (151 MHz, $CDCl_3$) δ 143.48, 137.36, 134.48, 131.51, 131.45, 129.73, 127.52, 120.40, 61.23, 49.27, 42.02, 29.89, 23.84, 21.57.

HRMS m/z (ESI) calcd. for $C_{18}H_{21}BrNO_2S^+$ $(M+H)^+$ 394.0471, found 394.0510.

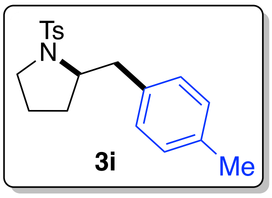

2-(4-methylbenzyl)-1-tosylpyrrolidine

3i was prepared following the general procedure 2.1 and purified by column chromatography (hexane:EA = 5:1) as colorless oil.

^{1}H NMR (600 MHz, Chloroform-*d*) δ 7.75 (d, *J* = 8.2 Hz, 2H), 7.31 (d, *J* = 7.9 Hz, 2H), 7.12 (q, *J* = 8.0 Hz, 4H), 3.83 – 3.77 (m, 1H), 3.40 (ddd, *J* = 10.7, 7.1, 4.4 Hz, 1H), 3.24 – 3.19 (m, 1H), 3.12 (dt, *J* = 9.9, 7.3 Hz, 1H), 2.70 (dd, *J* = 13.3, 9.7 Hz, 1H), 2.42 (s, 3H), 2.33 (s, 3H), 1.73 – 1.60 (m, 2H), 1.50 – 1.35 (m, 2H).

^{13}C NMR (151 MHz, $CDCl_3$) δ 143.29, 135.93, 135.42, 134.66, 129.64, 129.50, 129.11, 127.52, 61.73, 49.24, 42.28, 29.78, 23.78, 21.53, 21.06.

HRMS m/z (ESI) calcd. for $C_{19}H_{24}NO_2S^+$ $(M+H)^+$ 330.1522, found 330.1551.

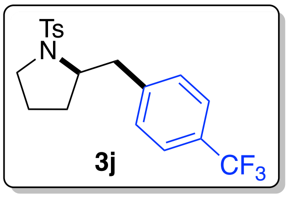

1-tosyl-2-(4-(trifluoromethyl)benzyl)pyrrolidine

3j was prepared following the general procedure 2.1 and purified by column chromatography (hexane:EA = 10:1) as white solid.

^{1}H NMR (600 MHz, Chloroform-*d*) δ 7.75 (d, *J* = 7.9 Hz, 2H), 7.56 (d, *J* = 7.9 Hz, 2H), 7.37 (d, *J* = 7.9 Hz, 2H), 7.33 (d, *J* = 7.9 Hz, 2H), 3.82 (tt, *J* = 7.6, 3.6 Hz, 1H), 3.37 (ddd, *J* = 10.2, 6.5, 4.0 Hz, 1H), 3.27 (dd, *J* = 13.4, 3.5 Hz, 1H), 3.14 (dt, *J* = 10.4, 7.1 Hz, 1H), 2.88 (dd, *J* = 13.3, 9.2 Hz, 1H), 2.43 (s, 3H), 1.68 – 1.51 (m, 2H), 1.46 (dq, *J* = 12.4, 7.2, 6.7 Hz, 2H).

^{13}C NMR (151 MHz, $CDCl_3$) δ 143.56, 142.52, 134.38, 130.02, 129.77, 128.81 (q, *J* = 32.4, 32.0 Hz), 127.52, 125.35, 125.32, 61.15, 49.28, 42.47, 29.96, 23.84, 21.56.

^{19}F NMR (564 MHz, Chloroform-*d*) δ -62.34.

HRMS m/z (ESI) calcd. for $C_{19}H_{21}F_3NO_2S^+$ $(M+H)^+$ 384.1240, found 384.1278.

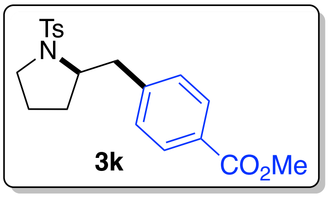

Methyl-4-((1-tosylpyrrolidin-2-yl)methyl)benzoate

3k was prepared following the general procedure 2.1 and purified by column chromatography (hexane:EA = 3:1) as yellow solid.

^{1}**H NMR** (600 MHz, Chloroform-*d*) δ 7.97 (d, *J* = 8.2 Hz, 2H), 7.75 (d, *J* = 8.3 Hz, 2H), 7.36 – 7.29 (m, 4H), 3.91 (s, 3H), 3.83 (tt, *J* = 7.1, 3.5 Hz, 1H), 3.40 – 3.35 (m, 1H), 3.27 (dd, *J* = 13.3, 3.6 Hz, 1H), 3.13 (dt, *J* = 10.0, 6.9 Hz, 1H), 2.87 (dd, *J* = 13.3, 9.2 Hz, 1H), 2.43 (s, 3H), 1.65 – 1.58 (m, 2H), 1.51 – 1.41 (m, 2H).
^{13}**C NMR** (151 MHz, $CDCl_3$) δ 167.07, 143.86, 143.47, 134.51, 129.72, 128.44, 127.51, 61.14, 52.07, 49.23, 42.68, 29.97, 23.82, 21.54.
HRMS m/z (ESI) calcd. for $C_{20}H_{24}NO_4S^+$ $(M+H)^+$ 374.1421, found 374.1449.

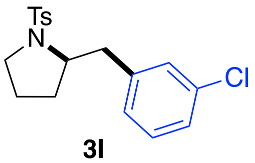

3l

2-(3-chlorobenzyl)-1-tosylpyrrolidine
3l was prepared following the general procedure 2.1 and purified by column chromatography (hexane:EA = 5:1) as pale yellow oil.
^{1}**H NMR** (600 MHz, Chloroform-*d*) δ 7.75 (d, *J* = 8.3 Hz, 2H), 7.34 – 7.30 (m, 2H), 7.22 (qd, *J* = 7.3, 1.5 Hz, 3H), 7.14 (dt, *J* = 7.2, 1.6 Hz, 1H), 3.81 (ddt, *J* = 10.4, 6.6, 3.4 Hz, 1H), 3.39 (ddd, *J* = 10.8, 6.8, 4.1 Hz, 1H), 3.21 (dd, *J* = 13.4, 3.6 Hz, 1H), 3.14 (dt, *J* = 9.9, 7.3 Hz, 1H), 2.75 (dd, *J* = 13.4, 9.5 Hz, 1H), 2.43 (s, 3H), 1.71 – 1.61 (m, 2H), 1.52 – 1.41 (m, 2H).
^{13}**C NMR** (151 MHz, $CDCl_3$) δ 143.45, 140.49, 134.62, 134.17, 129.73, 129.61, 127.88, 127.62, 127.53, 126.67, 61.24, 49.22, 42.35, 29.94, 23.84, 21.54.
HRMS m/z (ESI) calcd. for $C_{18}H_{21}ClNO_2S^+$ $(M+H)^+$ 350.0976, found 350.1006.

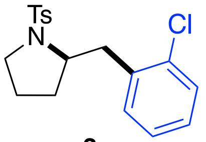

3m

2-(2-chlorobenzyl)-1-tosylpyrrolidine
3m was prepared following the general procedure 2.1 and purified by column chromatography (hexane:EA = 5:1) as colorless oil.
^{1}**H NMR** (600 MHz, Chloroform-*d*) δ 7.77 (d, *J* = 8.2 Hz, 2H), 7.35 (dd, *J* = 7.9, 1.4 Hz, 1H), 7.32 (td, *J* = 4.8, 2.4 Hz, 3H), 7.21 (td, *J* = 7.4, 1.4 Hz, 1H), 7.17 (td, *J* = 7.6, 1.8 Hz, 1H), 3.93 (ddd, *J* = 10.1, 7.8, 3.9 Hz, 1H), 3.47 (ddd, *J* = 10.1, 7.2, 4.3 Hz, 1H), 3.41 (dd, *J* = 13.4, 4.3 Hz, 1H), 3.17 (ddd, *J* = 10.0, 8.2, 7.0 Hz, 1H), 2.92 (dd, *J* = 13.4, 9.8 Hz, 1H), 2.42 (s, 3H), 1.86 – 1.74 (m, 1H), 1.64 (ddt, *J* = 13.8, 6.6, 3.9 Hz, 1H), 1.56 – 1.49 (m, 1H), 1.35 (ddt, *J* = 12.7, 9.5, 7.3 Hz, 1H).
^{13}**C NMR** (151 MHz, $CDCl_3$) δ 143.36, 136.36, 134.56, 134.38, 132.00, 129.68, 129.58, 127.96, 127.58, 126.86, 60.32, 49.29, 39.51, 29.74, 23.90, 21.56.
HRMS m/z (ESI) calcd. for $C_{18}H_{21}ClNO_2S^+$ $(M+H)^+$ 350.0976, found 350.1008.

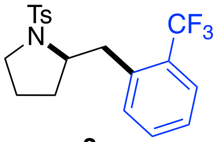

3n

1-tosyl-2-(2-(trifluoromethyl)benzyl)pyrrolidine
3n was prepared following the general procedure 2.1 and purified by column chromatography (hexane:EA = 10:1) as colorless oil.
^{1}**H NMR** (600 MHz, Chloroform-*d*) δ 7.75 (d, *J* = 8.3 Hz, 2H), 7.63 (d, *J* = 7.9 Hz, 1H), 7.53 – 7.47 (m, 2H), 7.32 (t, *J* = 7.8 Hz, 3H), 3.99 (tq, *J* = 8.6, 4.4, 3.8 Hz, 1H), 3.47 (ddd, *J* = 10.1, 7.1, 4.3 Hz, 1H), 3.40 (dd, *J* = 14.2, 4.9 Hz, 1H), 3.20 (ddd, *J* = 10.2, 8.2, 6.8 Hz, 1H), 3.04 – 2.95 (m, 1H), 2.43 (s, 3H), 1.87 – 1.76 (m, 1H), 1.57 – 1.49 (m, 2H), 1.42 – 1.32 (m, 1H).
^{13}**C NMR** (151 MHz, $CDCl_3$) δ 143.41, 137.18, 134.66, 132.10, 131.74, 129.72, 129.07 (q, *J* = 32.0, 32.0 Hz), 127.52, 126.52, 126.09, 126.05, 61.17, 49.07, 38.27, 29.73, 23.83, 21.56.
^{19}**F NMR** (564 MHz, Chloroform-*d*) δ -58.61.
HRMS m/z (ESI) calcd. for $C_{19}H_{21}F_3NO_2S^+$ $(M+H)^+$ 384.1240, found 384.1265.

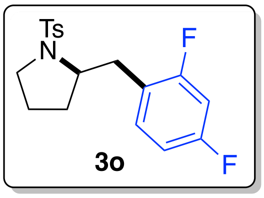

2-(2,4-difluorobenzyl)-1-tosylpyrrolidine

3o was prepared following the general procedure 2.1 and purified by column chromatography (hexane:EA = 5:1) as yellow oil.

^{1}H NMR (600 MHz, Chloroform-*d*) δ 7.74 (d, *J* = 8.3 Hz, 2H), 7.36 – 7.31 (m, 2H), 7.11 – 7.03 (m, 2H), 6.99 – 6.94 (m, 1H), 3.77 (tt, *J* = 7.4, 3.6 Hz, 1H), 3.37 (ddd, *J* = 9.5, 6.9, 4.3 Hz, 1H), 3.18 – 3.09 (m, 2H), 2.78 (dd, *J* = 13.5, 9.0 Hz, 1H), 2.44 (s, 3H), 1.66 – 1.58 (m, 2H), 1.51 – 1.38 (m, 2H).

^{13}C NMR (151 MHz, $CDCl_3$) δ 143.55, 135.33, 134.40, 129.77, 127.52, 125.62, 118.36 (d, *J* = 16.6 Hz), 117.12 (d, *J* = 17.0 Hz), 61.14, 49.27, 41.77, 29.93, 23.84, 21.57.

^{19}F NMR (564 MHz, Chloroform-*d*) δ -138.08, -141.25.

HRMS m/z (ESI) calcd. for $C_{18}H_{20}F_2NO_2S^+$ $(M+H)^+$ 352.1177, found 352.1189.

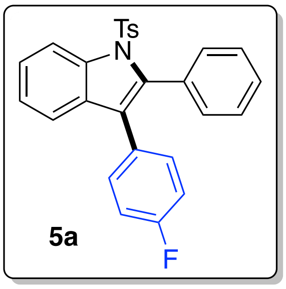

3-(4-fluorophenyl)-2-phenyl-1-tosyl-1*H*-indole

5a was prepared following the general procedure 2.1 and purified by column chromatography (hexane:EA = 10:1) as white solid.

^{1}H NMR (600 MHz, Chloroform-*d*) δ 8.41 (d, *J* = 8.4 Hz, 1H), 7.47 – 7.41 (m, 2H), 7.35 (dd, *J* = 12.2, 7.9 Hz, 4H), 7.32 – 7.27 (m, 4H), 7.23 (d, *J* = 7.3 Hz, 2H), 7.09 (d, *J* = 8.0 Hz, 2H), 7.05 (dd, *J* = 8.4, 5.5 Hz, 2H), 6.92 (t, *J* = 8.5 Hz, 2H), 2.32 (s, 3H).

^{13}C NMR (151 MHz, $CDCl_3$) δ 144.67, 137.12, 136.91, 135.31, 132.05, 131.45 (d, *J* = 8.0 Hz), 130.72, 130.26, 129.36, 128.63, 127.39, 126.96, 125.29, 124.24, 123.69, 119.74, 116.24, 115.29 (d, *J* = 21.3 Hz), 21.62.

^{19}F NMR (564 MHz, Chloroform-*d*) δ -114.90.

HRMS m/z (ESI) calcd. for $C_{27}H_{21}FNO_2S^+$ $(M+H)^+$ 442.1272, found 442.1317.

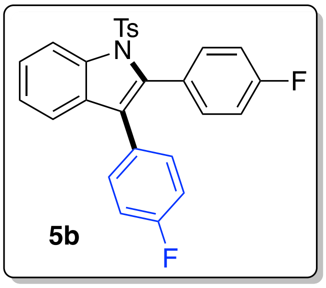

2,3-bis(4-fluorophenyl)-1-tosyl-1*H*-indole

5b was prepared following the general procedure 2.1 and purified by column chromatography (hexane:EA = 10:1) as white solid.

^{1}H NMR (600 MHz, Chloroform-*d*) δ 8.41 (dd, *J* = 9.0, 1.0 Hz, 1H), 7.43 (ddd, *J* = 7.7, 6.7, 1.6 Hz, 2H), 7.34 – 7.29 (m, 3H), 7.20 – 7.16 (m, 2H), 7.11 – 7.08 (m, 2H), 7.06 – 7.02 (m, 3H), 7.00 – 6.96 (m, 2H), 6.96 – 6.92 (m, 2H), 2.33 (s, 3H).

^{13}C NMR (151 MHz, $CDCl_3$) δ 163.70, 162.65, 162.05, 161.02, 144.79, 137.15, 135.73, 135.38, 133.86 (d, *J* = 8.4 Hz), 131.42 (d, *J* = 8.1 Hz), 130.11, 129.40, 129.27, 128.38, 126.86, 126.69, 126.67, 125.44, 124.31, 123.97, 119.77, 116.23, 115.40 (d, *J* = 21.5 Hz), 114.61 (d, *J* = 21.7 Hz), 21.59.

^{19}F NMR (564 MHz, Chloroform-*d*) δ -112.15, -114.60.

HRMS m/z (ESI) calcd. for $C_{27}H_{20}F_2NO_2S^+$ $(M+H)^+$ 460.1177, found 460.1193.

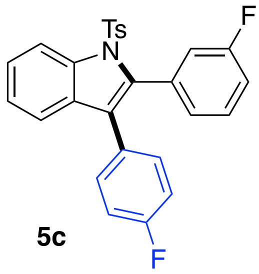

5c

2-(3-fluorophenyl)-3-(4-fluorophenyl)-1-tosyl-1*H*-indole

5c was prepared following the general procedure 2.1 and purified by column chromatography (hexane:EA = 10:1) as white solid.

^{1}H NMR (600 MHz, Chloroform-*d*) δ 8.40 (dt, *J* = 8.2, 1.0 Hz, 1H), 7.44 (ddd, *J* = 9.0, 7.6, 1.1 Hz, 2H), 7.38 – 7.35 (m, 2H), 7.31 (ddd, *J* = 8.1, 7.1, 1.0 Hz, 1H), 7.28 (dd, *J* = 8.0, 5.9 Hz, 1H), 7.13 – 7.10 (m, 2H), 7.09 – 7.03 (m, 4H), 6.97 – 6.93 (m, 2H), 6.90 (ddd, *J* = 9.5, 2.6, 1.5 Hz, 1H), 2.33 (s, 3H).

^{13}C NMR (151 MHz, $CDCl_3$) δ 162.74, 162.61, 161.10, 160.97, 144.92, 137.17, 135.15, 131.39 (d, *J* = 7.9 Hz), 130.14, 129.46, 128.93 (d, *J* = 8.1 Hz), 127.93, 126.91, 125.63, 124.39, 124.31, 119.93, 118.98, 116.26, 115.67 (d, *J* = 21.3 Hz), 115.46 (d, *J* = 21.6 Hz), 21.62.

^{19}F NMR (564 MHz, Chloroform-*d*) δ -113.71, -114.44.

HRMS m/z (ESI) calcd. for $C_{27}H_{20}F_2NO_2S^+$ $(M+H)^+$ 460.1177, found 460.1207.

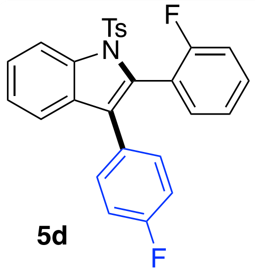

5d

2-(2-fluorophenyl)-3-(4-fluorophenyl)-1-tosyl-1*H*-indole

5d was prepared following the general procedure 2.1 and purified by column chromatography (hexane:EA = 10:1) as white solid.

^{1}H NMR (600 MHz, Chloroform-*d*) δ 8.35 (dt, *J* = 8.5, 0.8 Hz, 1H), 7.48 (dt, *J* = 7.9, 1.0 Hz, 1H), 7.46 – 7.40 (m, 3H), 7.40 – 7.36 (m, 1H), 7.30 (td, *J* = 7.5, 1.0 Hz, 1H), 7.16 (td, *J* = 7.4, 1.8 Hz, 1H), 7.14 – 7.07 (m, 5H), 7.05 (ddd, *J* = 9.5, 8.3, 1.1 Hz, 1H), 6.94 (t, *J* = 8.7 Hz, 2H), 2.34 (s, 3H).

^{13}C NMR (151 MHz, $CDCl_3$) δ 144.81, 136.92, 135.46, 133.78, 131.16 (d, *J* = 7.6 Hz), 131.04 (d, *J* = 7.8 Hz)129.81, 129.52, 126.95, 125.46, 124.72, 124.03, 123.29, 119.88, 115.63, 115.43 (d, *J* = 7.4 Hz), 115.29 (d, *J* = 7.8 Hz), 21.63.

^{19}F NMR (564 MHz, Chloroform-*d*) δ -110.23, -114.58.

HRMS m/z (ESI) calcd. for $C_{27}H_{20}F_2NO_2S^+$ $(M+H)^+$ 460.1177, found 460.1209.

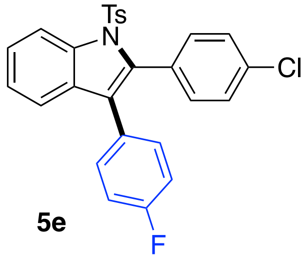

5e

2-(4-chlorophenyl)-3-(4-fluorophenyl)-1-tosyl-1*H*-indole

5e was prepared following the general procedure 2.1 and purified by column chromatography (hexane:EA = 10:1) as pale yellow solid.

^{1}H NMR (400 MHz, Chloroform-*d*) δ 8.40 (d, *J* = 8.4 Hz, 1H), 7.42 (d, *J* = 8.0 Hz, 2H), 7.31 (dd, *J* = 12.0, 8.5 Hz, 5H), 7.16 (d, *J* = 8.2 Hz, 2H), 7.09 (d, *J* = 8.0 Hz, 2H), 7.03 (dd, *J* = 8.6, 5.6 Hz, 2H), 6.95 (t, *J* = 8.7 Hz, 2H), 2.33 (s, 3H).

^{13}C NMR (101 MHz, $CDCl_3$) δ 163.12, 144.85, 135.19, 134.77, 133.25, 131.42 (d, *J* = 8.0 Hz), 129.42, 129.24, 127.76, 126.86, 125.56, 124.41, 119.84, 116.32, 115.48 (d, *J* = 21.4 Hz), 21.60.

^{19}F NMR (564 MHz, Chloroform-*d*) δ -114.41.

HRMS m/z (ESI) calcd. for $C_{27}H_{20}ClFNO_2S^+$ $(M+H)^+$ 476.0882, found 476.0912.

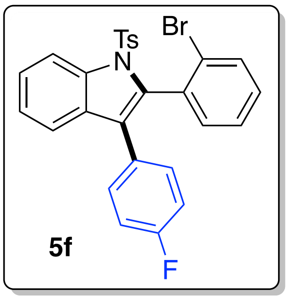

2-(2-bromophenyl)-3-(4-fluorophenyl)-1-tosyl-1*H*-indole

5f was prepared following the general procedure 2.1 and purified by column chromatography (hexane:EA = 10:1) as pale yellow solid.

^{1}H NMR (600 MHz, Chloroform-*d*) δ 8.38 (dt, *J* = 8.4, 0.9 Hz, 1H), 7.58 – 7.54 (m, 1H), 7.54 – 7.49 (m, 3H), 7.45 (ddd, *J* = 8.5, 7.2, 1.3 Hz, 1H), 7.35 – 7.30 (m, 1H), 7.28 (dd, *J* = 7.2, 1.9 Hz, 2H), 7.22 (dd, *J* = 6.8, 2.5 Hz, 1H), 7.17 – 7.10 (m, 4H), 6.97 – 6.90 (m, 2H), 2.35 (s, 3H).

^{13}C NMR (151 MHz, $CDCl_3$) δ 162.72, 161.09, 144.90, 136.38, 135.89, 134.48, 133.98, 132.42, 131.08 (d, *J* = 7.7 Hz), 130.52, 129.59, 129.40, 128.36, 127.25, 126.86, 126.33, 125.46, 123.89, 123.50, 120.00, 115.37 (d, *J* = 8.8 Hz), 115.26, 21.65.

^{19}F NMR (564 MHz, Chloroform-*d*) δ -114.63.

HRMS m/z (ESI) calcd. for $C_{27}H_{20}BrFNO_2S^+$ $(M+H)^+$ 522.0356, found 522.0381.

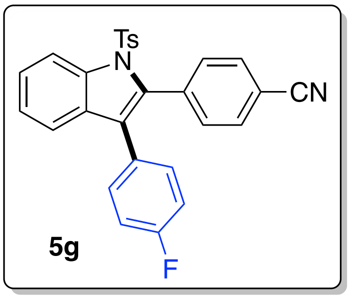

4-(3-(4-fluorophenyl)-1-tosyl-1*H*-indol-2-yl)benzonitrile

5g was prepared following the general procedure 2.1 and purified by column chromatography (hexane:EA = 5:1) as white solid.

^{1}H NMR (600 MHz, Chloroform-*d*) δ 8.39 (d, *J* = 8.4 Hz, 1H), 7.60 (d, *J* = 8.1 Hz, 2H), 7.47 (ddd, *J* = 8.5, 7.1, 1.3 Hz, 1H), 7.40 (dd, *J* = 16.9, 8.0 Hz, 3H), 7.32 (dd, *J* = 15.5, 7.9 Hz, 3H), 7.11 (d, *J* = 8.1 Hz, 2H), 7.02 – 6.94 (m, 4H), 2.33 (s, 3H).

^{13}C NMR (151 MHz, $CDCl_3$) δ 149.14, 148.87, 148.61, 145.16, 137.47, 135.78, 134.70, 134.65, 132.50, 131.42 (d, *J* = 8.0 Hz), 131.18, 129.54, 126.77, 126.14, 125.66, 124.76, 120.16, 116.46, 115.73 (d, *J* = 21.7 Hz), 112.18, 21.65.

^{19}F NMR (564 MHz, Chloroform-*d*) δ -113.68.

HRMS m/z (ESI) calcd. for $C_{28}H_{20}FN_2O_2S^+$ $(M+H)^+$ 467.1224, found 467.1256.

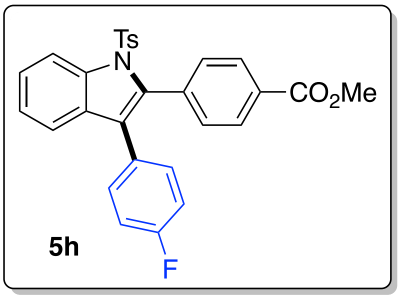

methyl 4-(3-(4-fluorophenyl)-1-tosyl-1*H*-indol-2-yl)benzoate

5h was prepared following the general procedure 2.1 and purified by column chromatography (hexane:EA = 4:1) as white solid.

^{1}H NMR (600 MHz, Chloroform-*d*) δ 8.40 (dt, *J* = 8.4, 0.9 Hz, 1H), 7.98 (d, *J* = 8.4 Hz, 2H), 7.49 – 7.38 (m, 2H), 7.36 – 7.31 (m, 5H), 7.12 – 7.08 (m, 2H), 7.04 – 6.99 (m, 2H), 6.96 – 6.90 (m, 2H), 3.94 (s, 3H), 2.33 (s, 3H).

^{13}C NMR (151 MHz, $CDCl_3$) δ 166.80, 162.73, 161.09, 144.92, 137.34, 135.68, 135.57, 134.92, 131.97, 131.40 (d, *J* = 8.2 Hz), 130.31, 129.97, 129.44, 128.59, 128.06, 126.84, 125.72, 124.80, 124.49, 119.95, 116.35, 115.49 (d, *J* = 21.7 Hz), 52.27, 21.61.

^{19}F NMR (564 MHz, Chloroform-*d*) δ -114.29.

HRMS m/z (ESI) calcd. for $C_{29}H_{24}FNO_4S^+$ $(M+H)^+$ 500.1326, found 500.1352.

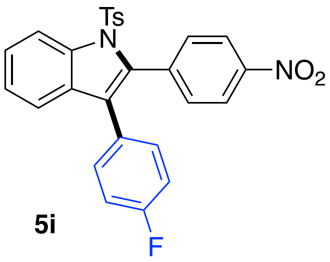

5i

3-(4-fluorophenyl)-2-(4-nitrophenyl)-1-tosyl-1*H*-indole
5i was prepared following the general procedure 2.1 and purified by column chromatography (hexane:EA = 3:1) as yellow solid.
^{1}**H NMR** (600 MHz, Chloroform-*d*) δ 8.39 (d, *J* = 8.4 Hz, 1H), 8.17 (d, *J* = 8.8 Hz, 2H), 7.52 – 7.44 (m, 3H), 7.44 – 7.40 (m, 1H), 7.37 – 7.29 (m, 3H), 7.12 (d, *J* = 8.2 Hz, 2H), 7.04 – 6.92 (m, 4H), 2.34 (s, 3H).
^{13}**C NMR** (151 MHz, $CDCl_3$) δ 149.95, 134.57, 132.69, 131.42 (d, *J* = 8.2 Hz)., 130.40, 129.58, 126.77, 126.27, 124.85, 124.29, 124.12, 123.08, 122.67, 120.23, 116.50, 115.80 (d, *J* = 21.9 Hz), 21.66.
^{19}**F NMR** (564 MHz, Chloroform-*d*) δ -113.51.
HRMS m/z (ESI) calcd. for $C_{27}H_{20}FN_2O_4S^+$ $(M+H)^+$ 487.1122, found 487.1168.

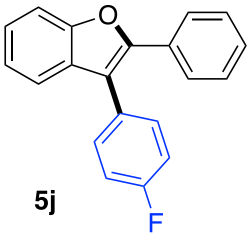

5j

3-(4-fluorophenyl)-2-phenylbenzofuran
5j was prepared following the general procedure 2.1 and purified by column chromatography (hexane:EA = 40:1) as white solid.
^{1}**H NMR** (600 MHz, Chloroform-*d*) δ 7.66 – 7.61 (m, 2H), 7.56 (dt, *J* = 8.2, 0.8 Hz, 1H), 7.49 – 7.44 (m, 3H), 7.36 – 7.30 (m, 4H), 7.27 – 7.23 (m, 1H), 7.19 – 7.15 (m, 2H).
^{13}**C NMR** (151 MHz, $CDCl_3$) δ 163.20, 161.56, 153.97, 150.67, 131.46 (d, *J* = 7.9 Hz), 130.50, 130.16, 128.83, 128.55, 127.02, 124.96, 124.84, 123.05, 119.83, 116.49, 116.12 (d, *J* = 21.4 Hz), 111.23, 101.33.
^{19}**F NMR** (564 MHz, Chloroform-*d*) δ -114.19.

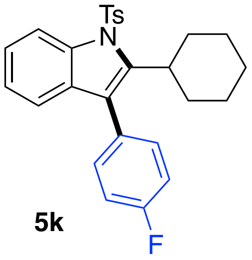

5k

2-cyclohexyl-3-(4-fluorophenyl)-1-tosyl-1*H*-indole
5k was prepared following the general procedure 2.1 and purified by column chromatography (hexane:EA = 10:1) as colorless oil.
^{1}**H NMR** (600 MHz, Chloroform-*d*) δ 8.28 (dd, *J* = 8.4, 0.9 Hz, 1H), 7.65 (d, *J* = 8.4 Hz, 2H), 7.30 (ddd, *J* = 8.5, 7.2, 1.3 Hz, 1H), 7.24 (d, *J* = 8.2 Hz, 2H), 7.22 – 7.19 (m, 2H), 7.16 (td, *J* = 7.5, 0.9 Hz, 1H), 7.12 – 7.07 (m, 2H), 6.97 (dt, *J* = 7.7, 1.0 Hz, 1H), 3.51 – 3.40 (m, 1H), 2.38 (s, 3H), 1.66 – 1.60 (m, 6H), 1.26 – 1.12 (m, 4H).
^{13}**C NMR** (151 MHz, $CDCl_3$) δ 163.10, 161.46, 144.64, 142.22, 136.94, 135.85, 132.39 (d, *J* = 8.2 Hz), 132.07, 130.30, 129.79, 126.30, 124.39, 123.38, 121.63, 118.95, 115.20, 115.10 (d, *J* = 12.4 Hz), 38.03, 33.40, 27.10, 25.82, 21.62.
^{19}**F NMR** (564 MHz, Chloroform-*d*) δ -114.72.
HRMS m/z (ESI) calcd. for $C_{27}H_{27}FNO_2S^+$ $(M+H)^+$ 448.1741, found 448.1774.

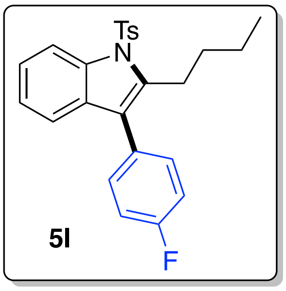

2-butyl-3-(4-fluorophenyl)-1-tosyl-1*H*-indole
5l was prepared following the general procedure 2.1 and purified by column chromatography (hexane:EA = 10:1) as colorless oil.
^{1}H NMR (600 MHz, Chloroform-*d*) δ 8.22 (d, *J* = 8.4 Hz, 1H), 7.63 (d, *J* = 8.4 Hz, 2H), 7.32 – 7.23 (m, 4H), 7.22 – 7.17 (m, 3H), 7.17 – 7.12 (m, 2H), 3.00 – 2.92 (m, 2H), 2.34 (s, 3H), 1.74 – 1.65 (m, 2H), 1.27 (dq, *J* = 14.8, 7.5 Hz, 2H), 0.82 (t, *J* = 7.4 Hz, 3H).
^{13}C NMR (151 MHz, $CDCl_3$) δ 163.00, 161.37, 144.66, 138.78, 136.56, 135.91, 131.60 (d, *J* = 7.9 Hz), 130.63, 129.76, 129.23, 126.33, 124.43, 123.79, 122.66, 119.09, 115.63 (d, *J* = 21.3 Hz), 115.31, 33.38, 26.59, 22.53, 21.60, 13.70.
^{19}F NMR (564 MHz, Chloroform-*d*) δ -114.58.
HRMS m/z (ESI) calcd. for $C_{25}H_{25}FNO_2S^+$ $(M+H)^+$ 422.1585, found 422.1619.

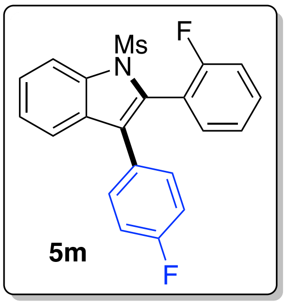

2-(2-fluorophenyl)-3-(4-fluorophenyl)-1-(methylsulfonyl)-1*H*-indole
5m was prepared following the general procedure 2.1 and purified by column chromatography (hexane:EA = 10:1) as white solid.
^{1}H NMR (600 MHz, Chloroform-*d*) δ 8.15 (dt, *J* = 8.5, 0.9 Hz, 1H), 7.60 (dt, *J* = 7.9, 1.0 Hz, 1H), 7.46 (ddd, *J* = 8.4, 7.2, 1.3 Hz, 1H), 7.43 – 7.35 (m, 2H), 7.25 – 7.21 (m, 3H), 7.14 – 7.08 (m, 2H), 7.00 (t, *J* = 8.7 Hz, 2H), 3.09 (s, 3H).
^{13}C NMR (151 MHz, $CDCl_3$) δ 162.90, 162.06, 161.26, 160.43, 136.43, 133.39, 131.25 (d, *J* = 8.6 Hz), 131.17 (d, *J* = 7.8 Hz), 129.95, 129.64, 128.13, 125.73, 124.22, 123.87, 120.19, 119.31, 119.21, 115.54 (d, *J* = 21.6 Hz), 115.22 (d, *J* = 21.8 Hz), 114.71, 40.59.
^{19}F NMR (564 MHz, Chloroform-*d*) δ -111.74, -114.32.
HRMS m/z (ESI) calcd. for $C_{21}H_{26}F_2NO_2S^+$ $(M+H)^+$ 384.0864, found 384.0894.

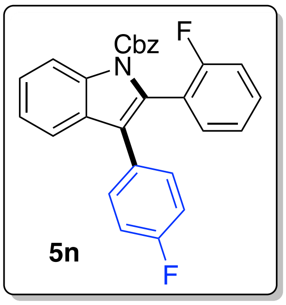

benzyl 2-(2-fluorophenyl)-3-(4-fluorophenyl)-1*H*-indole-1-carboxylate
5n was prepared following the general procedure 2.1 and purified by column chromatography (hexane:EA = 10:1) as white solid.
^{1}H NMR (600 MHz, Chloroform-*d*) δ 8.30 (d, *J* = 8.4 Hz, 1H), 7.54 (d, *J* = 7.8 Hz, 1H), 7.40 (ddd, *J* = 8.5, 7.3, 1.2 Hz, 1H), 7.33 – 7.28 (m, 4H), 7.22 – 7.18 (m, 3H), 7.13 (dd, *J* = 7.7, 1.9 Hz, 2H), 7.05 (td, *J* = 7.4, 1.8 Hz, 1H), 7.00 – 6.89 (m, 4H), 5.23 (d, *J* = 8.0 Hz, 2H).
^{13}C NMR (151 MHz, $CDCl_3$) δ 161.84, 161.10, 160.20, 158.72, 151.34, 136.38, 134.39, 132.03, 131.39 (d, *J* = 8.0 Hz), 130.17 (d, *J* = 8.0 Hz), 129.60, 129.30, 128.53, 128.51, 125.42, 123.53, 123.46, 123.14, 119.62, 115.84, 115.37 (d, *J* = 21.5 Hz), 114.96 (d, *J* = 21.3 Hz), 68.87.
^{19}F NMR (564 MHz, Chloroform-*d*) δ -113.00, -114.91.

HRMS m/z (ESI) calcd. for $C_{28}H_{20}F_2NO_2^+$ $(M+H)^+$ 440.1457, found 440.1489.

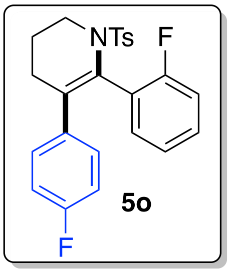

6-(2-fluorophenyl)-5-(4-fluorophenyl)-1-tosyl-1,2,3,4-tetrahydropyridine

5o was prepared following the general procedure 2.1 and purified by column chromatography (hexane:EA = 10:1) as white solid.

^{1}H NMR (600 MHz, Chloroform-*d*) δ 7.45 (d, *J* = 7.9 Hz, 2H), 7.17 (d, *J* = 7.9 Hz, 2H), 7.07 (dt, *J* = 7.7, 5.6 Hz, 2H), 6.88 (qd, *J* = 8.1, 6.0 Hz, 3H), 6.77 (t, *J* = 8.5 Hz, 2H), 6.63 (dd, *J* = 10.0, 8.1 Hz, 1H), 3.84 (s, 2H), 2.39 (d, *J* = 18.2 Hz, 5H), 1.82 (s, 2H).

^{13}C NMR (151 MHz, $CDCl_3$) δ 166.10, 164.58, 162.18, 161.04, 143.11, 137.55, 137.00, 132.76, 130.57, 130.14 (d, *J* = 7.7 Hz), 129.72, 129.43 (d, *J* = 8.4 Hz), 129.27, 127.17, 123.20, 114.85 (d, *J* = 22.3 Hz), 114.68 (d, *J* = 21.2 Hz), 46.21, 29.69, 22.86, 21.58.

^{19}F NMR (564 MHz, Chloroform-*d*) δ -113.85, -115.69.

HRMS m/z (ESI) calcd. for $C_{24}H_{22}F_2NO_2S^+$ $(M+H)^+$ 426.1334, found 426.1355.

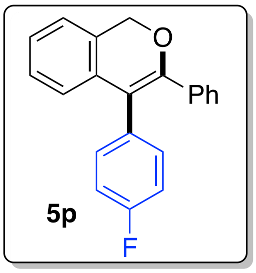

4-(4-fluorophenyl)-3-phenyl-1*H*-isochromene

5p was prepared following the general procedure 2.1 and purified by column chromatography (hexane:EA = 40:1) as white solid.

^{1}H NMR (600 MHz, Chloroform-*d*) δ 7.24 – 7.12 (m, 10H), 7.04 (ddd, *J* = 8.7, 7.7, 1.2 Hz, 2H), 6.80 (d, *J* = 7.4 Hz, 1H), 5.28 (s, 2H).

^{13}C NMR (151 MHz, $CDCl_3$) δ 151.64, 135.18, 133.60, 133.20 (d, *J* = 8.0 Hz), 128.58, 128.05, 127.66, 126.82, 123.74, 122.80, 115.94, 115.66 (d, *J* = 21.1 Hz), 68.76.

^{19}F NMR (564 MHz, Chloroform-*d*) δ -115.19.

HRMS m/z (ESI) calcd. for $C_{21}H_{16}FO^+$ $(M+H)^+$ 303.1180, found 303.1200.

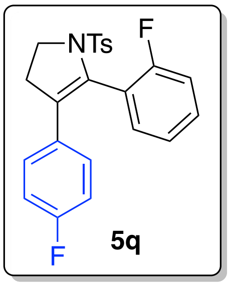

5-(2-fluorophenyl)-4-(4-fluorophenyl)-1-tosyl-2,3-dihydro-1*H*-pyrrole

5q was prepared following the general procedure 2.1 and purified by column chromatography (hexane:EA = 10:1) as pale yellow oil.

^{1}H NMR (600 MHz, Chloroform-*d*) δ 7.62 (d, *J* = 8.3 Hz, 2H), 7.40 – 7.33 (m, 2H), 7.28 (d, *J* = 8.0 Hz, 3H), 7.15 (td, *J* = 7.5, 1.1 Hz, 1H), 7.02 (ddd, *J* = 10.1, 8.3, 1.1 Hz, 1H), 6.86 – 6.76 (m, 4H), 4.08 (t, *J* = 8.6 Hz, 2H), 2.59 (t, *J* = 8.6 Hz, 2H), 2.44 (s, 3H).

^{13}C NMR (151 MHz, $CDCl_3$) δ 160.94, 143.83, 134.96, 132.36, 131.70, 131.21, 130.86 (d, *J* = 8.2 Hz), 129.56, 128.03 (d, *J* = 8.0 Hz), 127.78, 127.35, 125.56, 124.02, 123.61, 122.25, 120.63, 115.97 (d, *J* = 21.4 Hz), 115.16 (d, *J* = 21.6 Hz), 49.30, 33.07, 21.67.

^{19}F NMR (564 MHz, Chloroform-*d*) δ -111.31, -114.17.

HRMS m/z (ESI) calcd. for $C_{23}H_{20}F_2NO_2S^+$ $(M+H)^+$ 412.1177, found 412.1199.

5. NMR Spectra Data

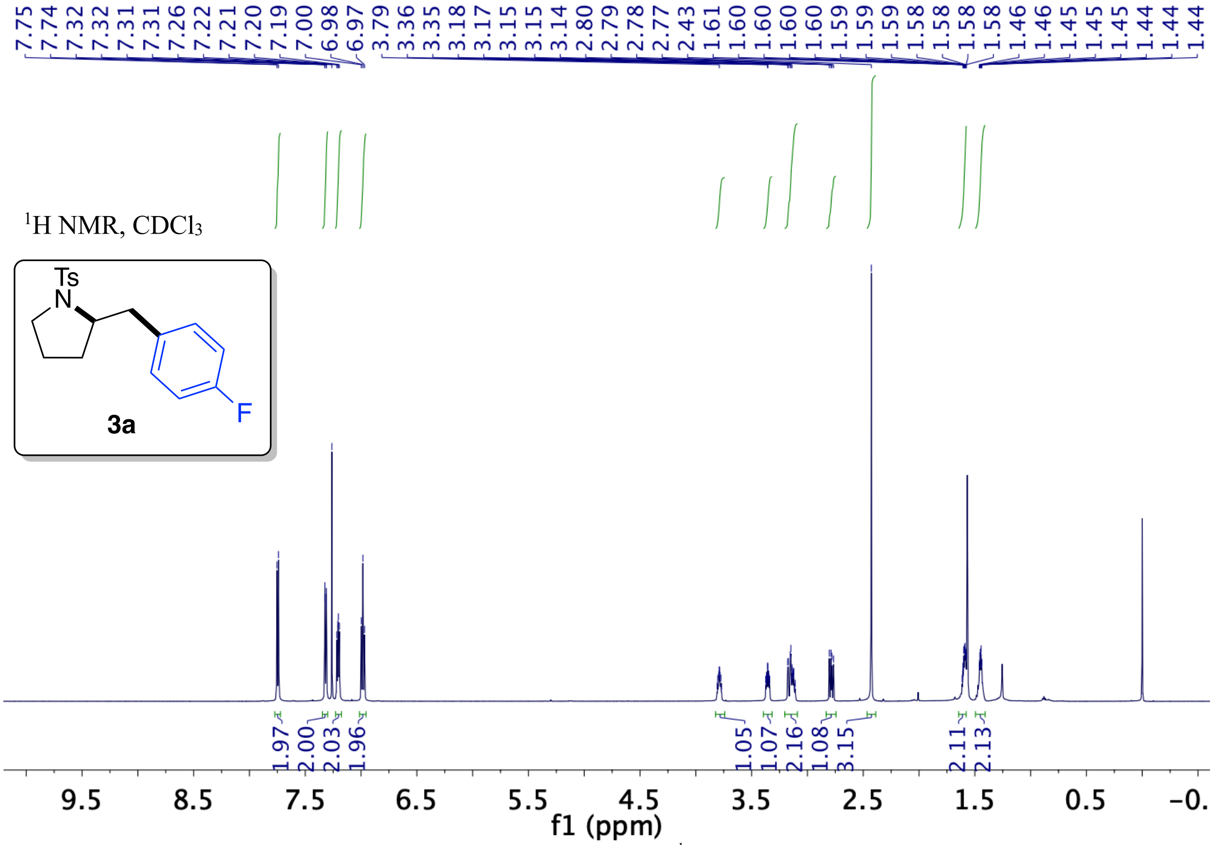

Supplementary Fig. 5. ^{1}H NMR Spectrum for **3a**

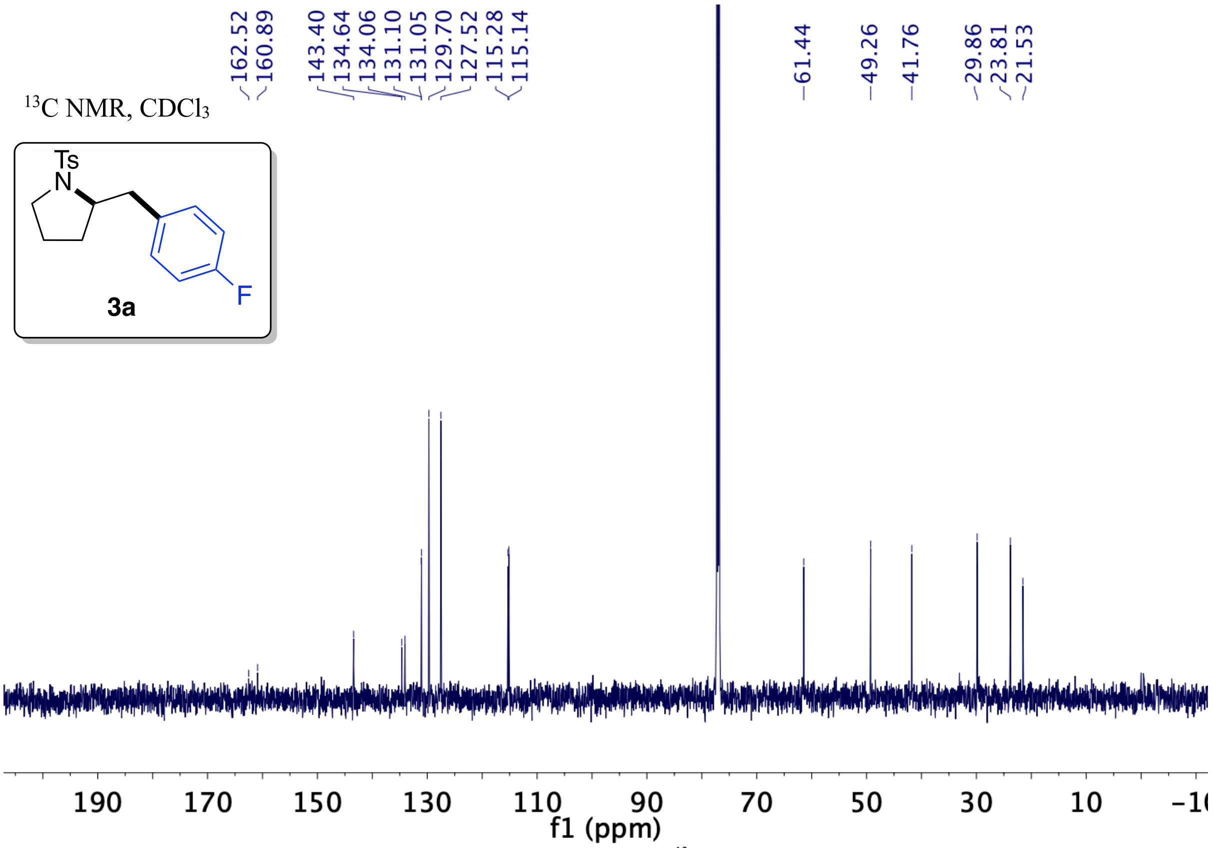

Supplementary Fig. 6. ^{13}C NMR Spectrum for **3a**

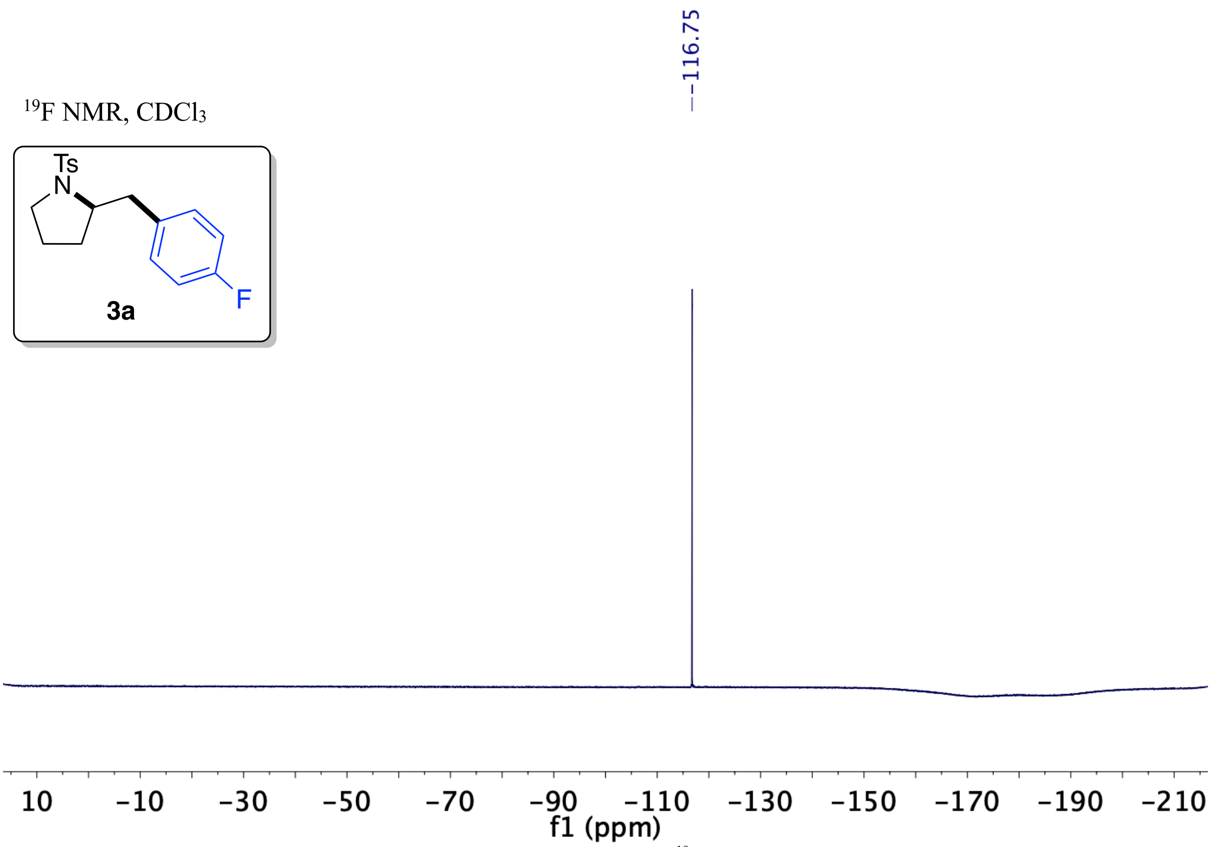

Supplementary Fig. 7. ^{19}F NMR Spectrum for **3a**

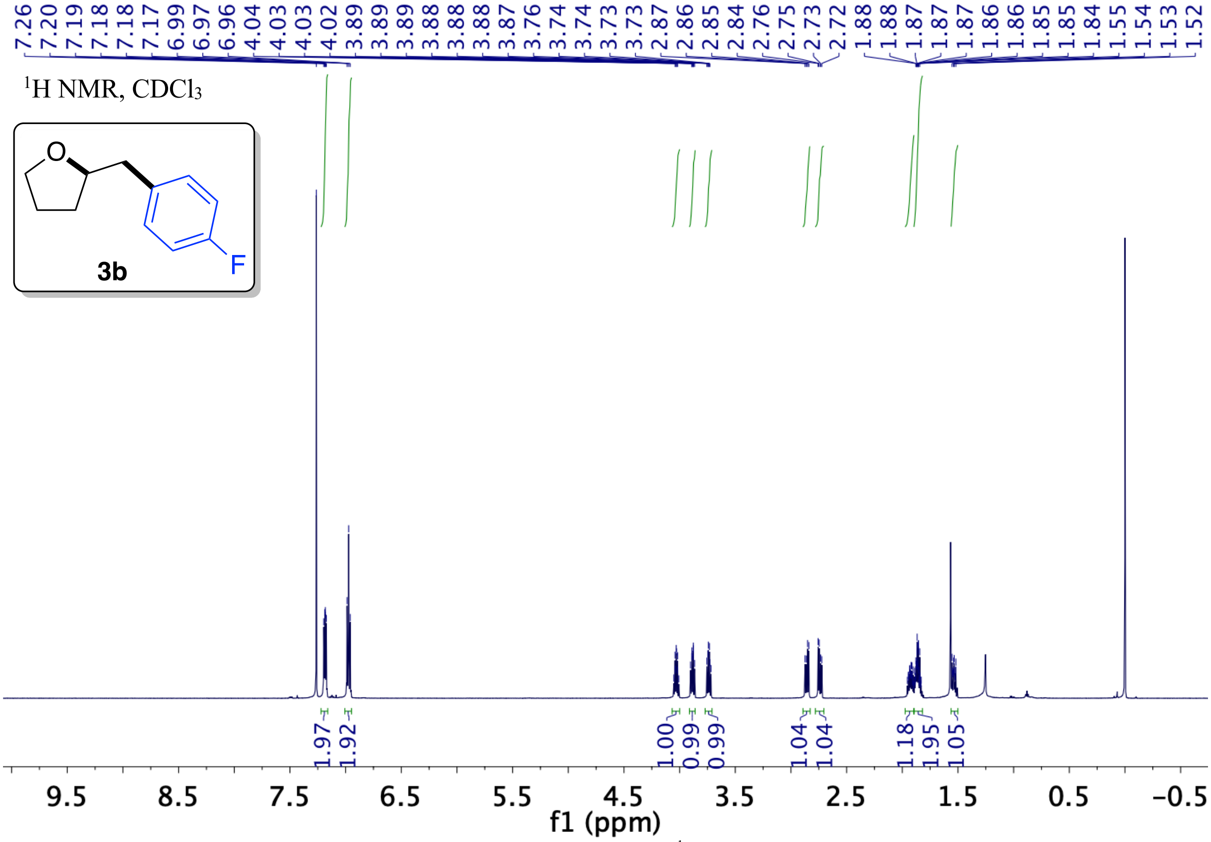

Supplementary Fig. 8. ^{1}H NMR Spectrum for **3b**

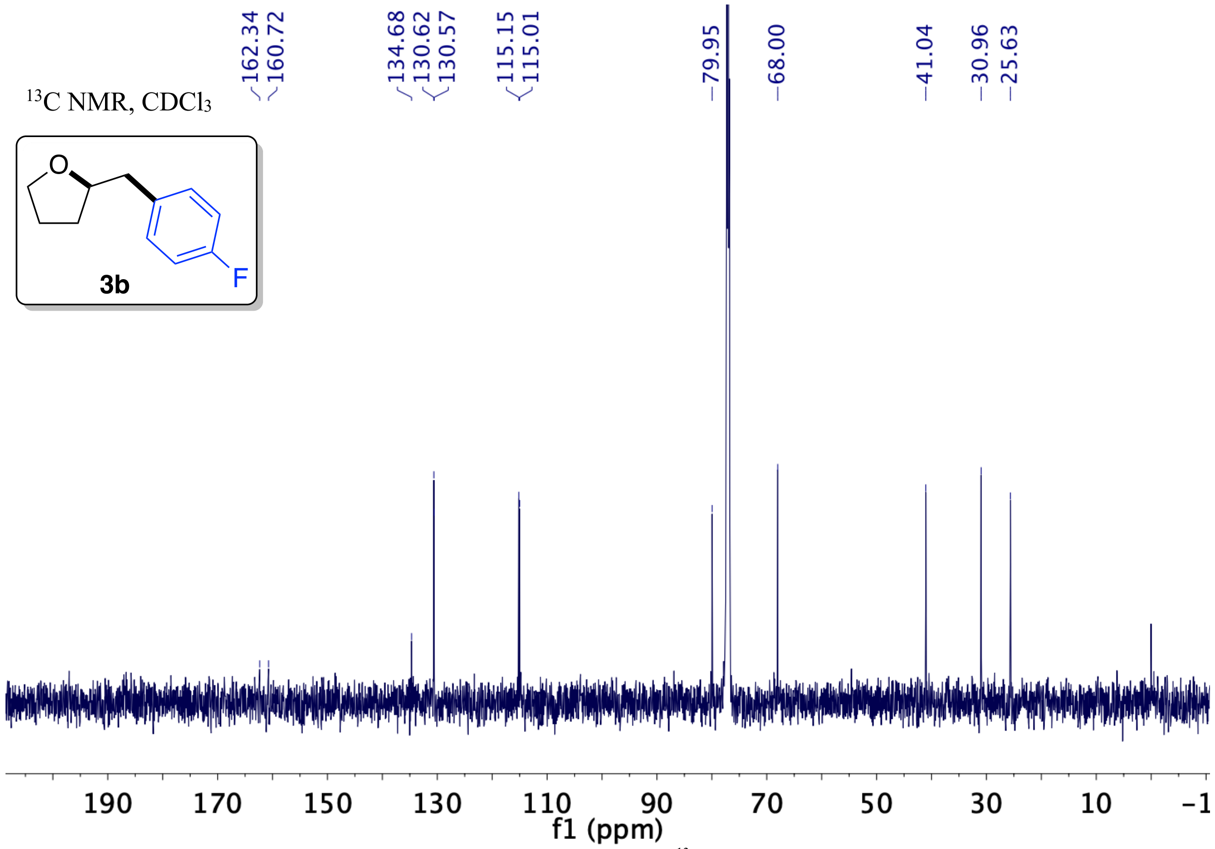

Supplementary Fig. 9. ^{13}C NMR Spectrum for **3b**

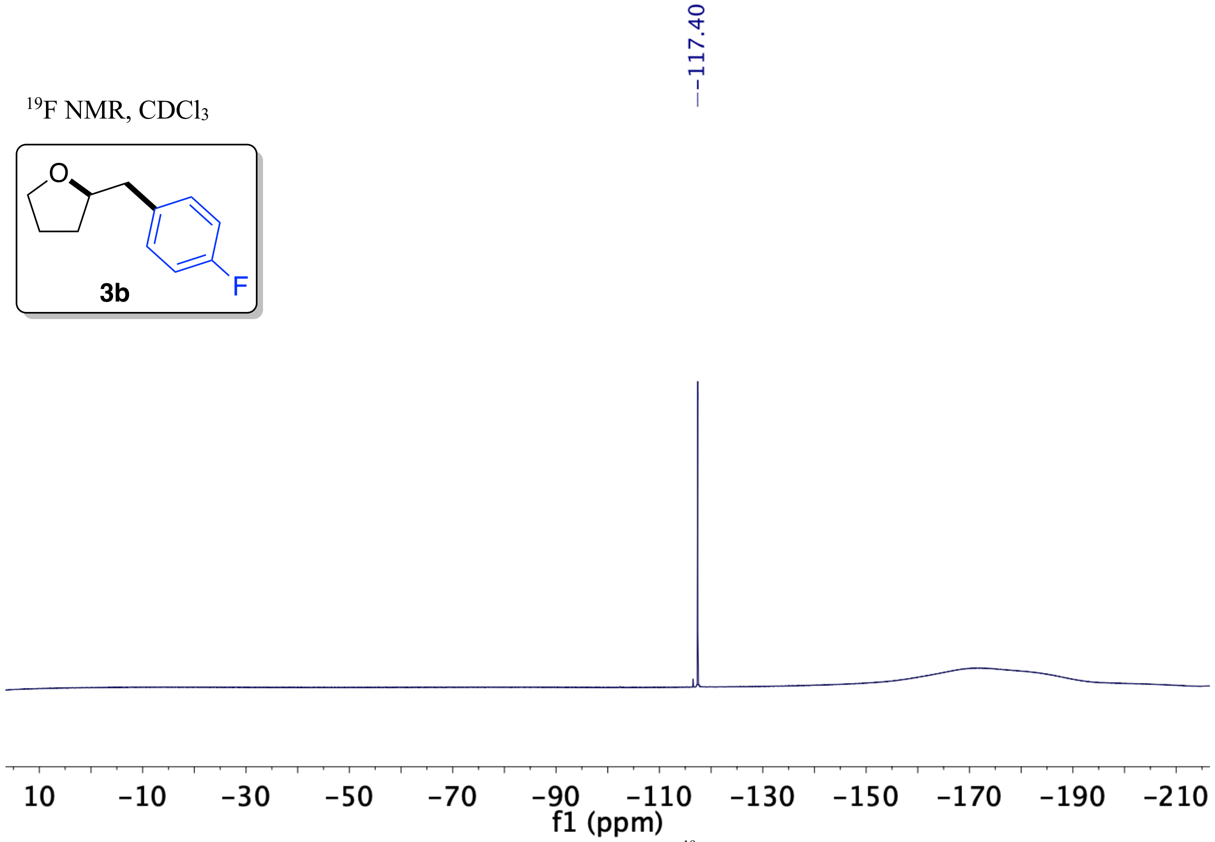

Supplementary Fig. 10. ^{19}F NMR Spectrum for **3b**

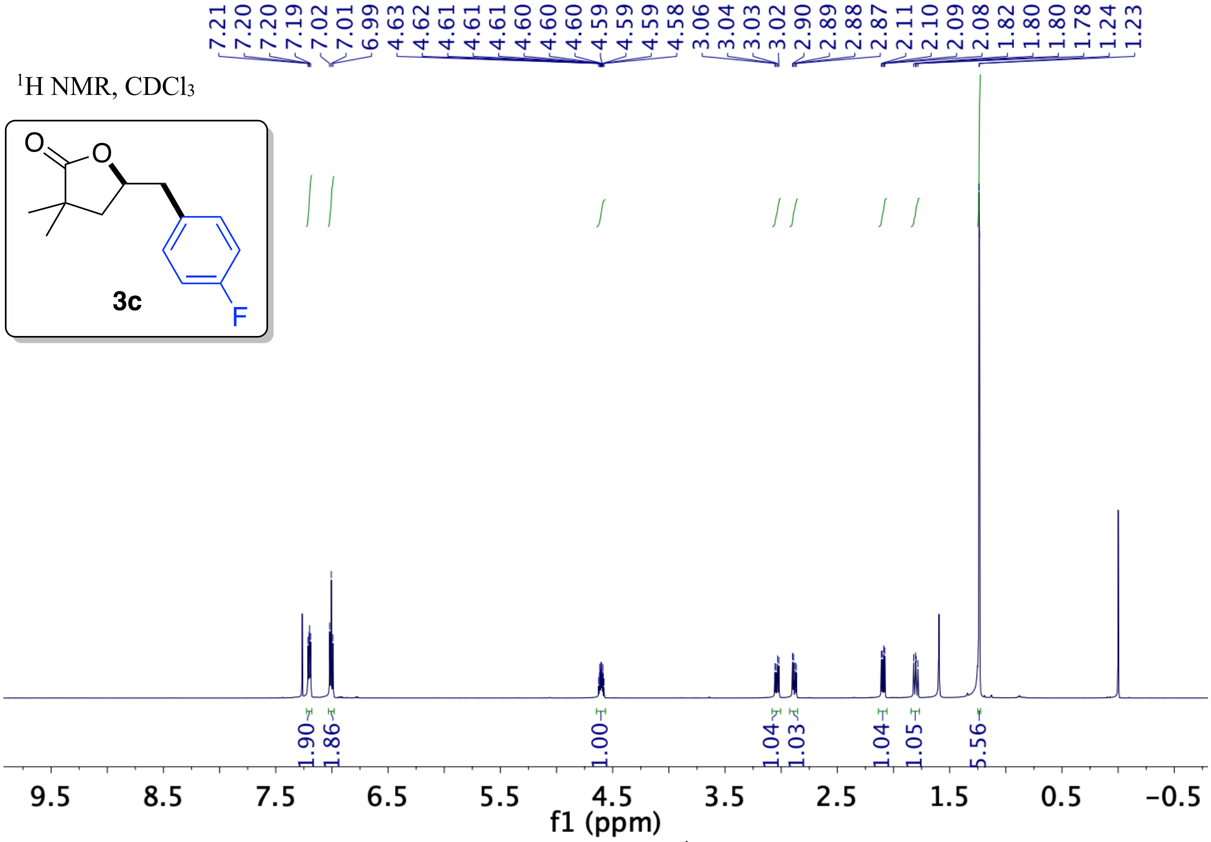

Supplementary Fig. 11. ^{1}H NMR Spectrum for **3c**

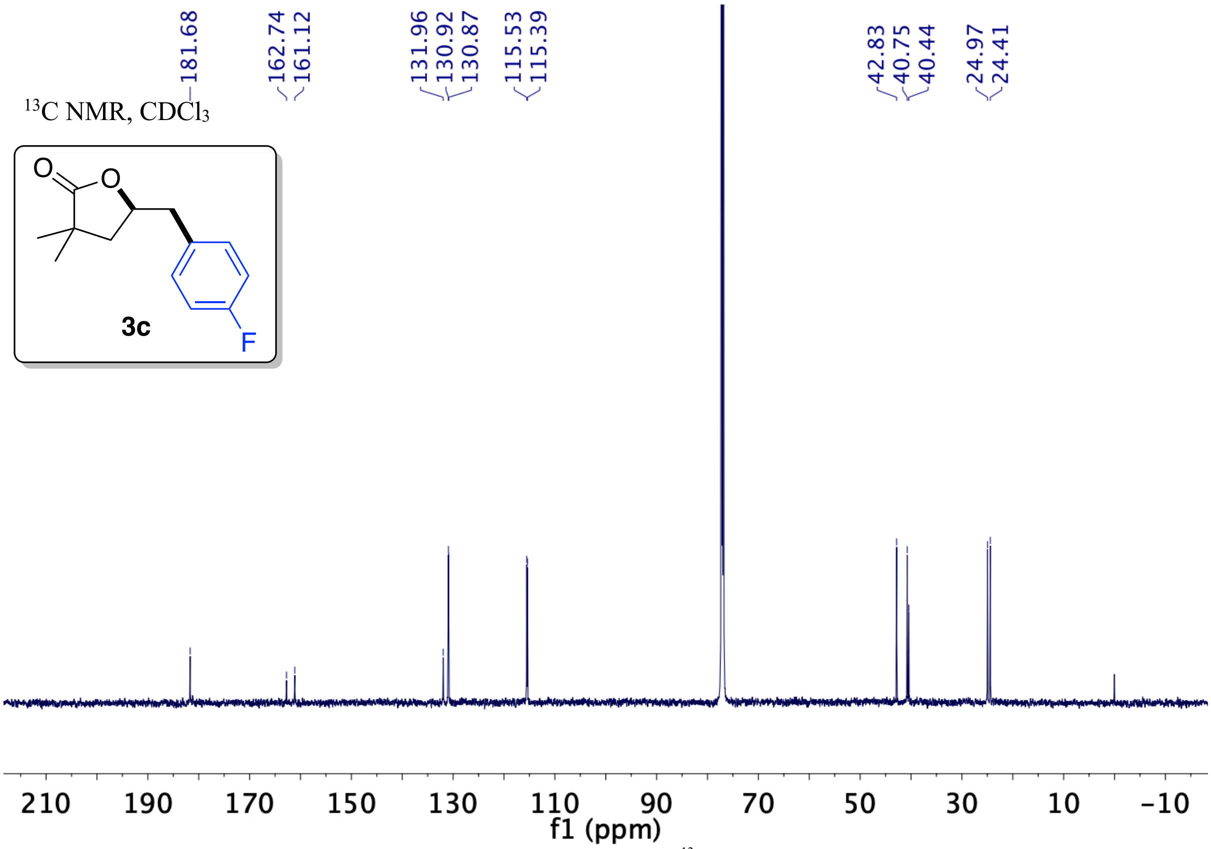

Supplementary Fig. 12. ^{13}C NMR Spectrum for **3c**

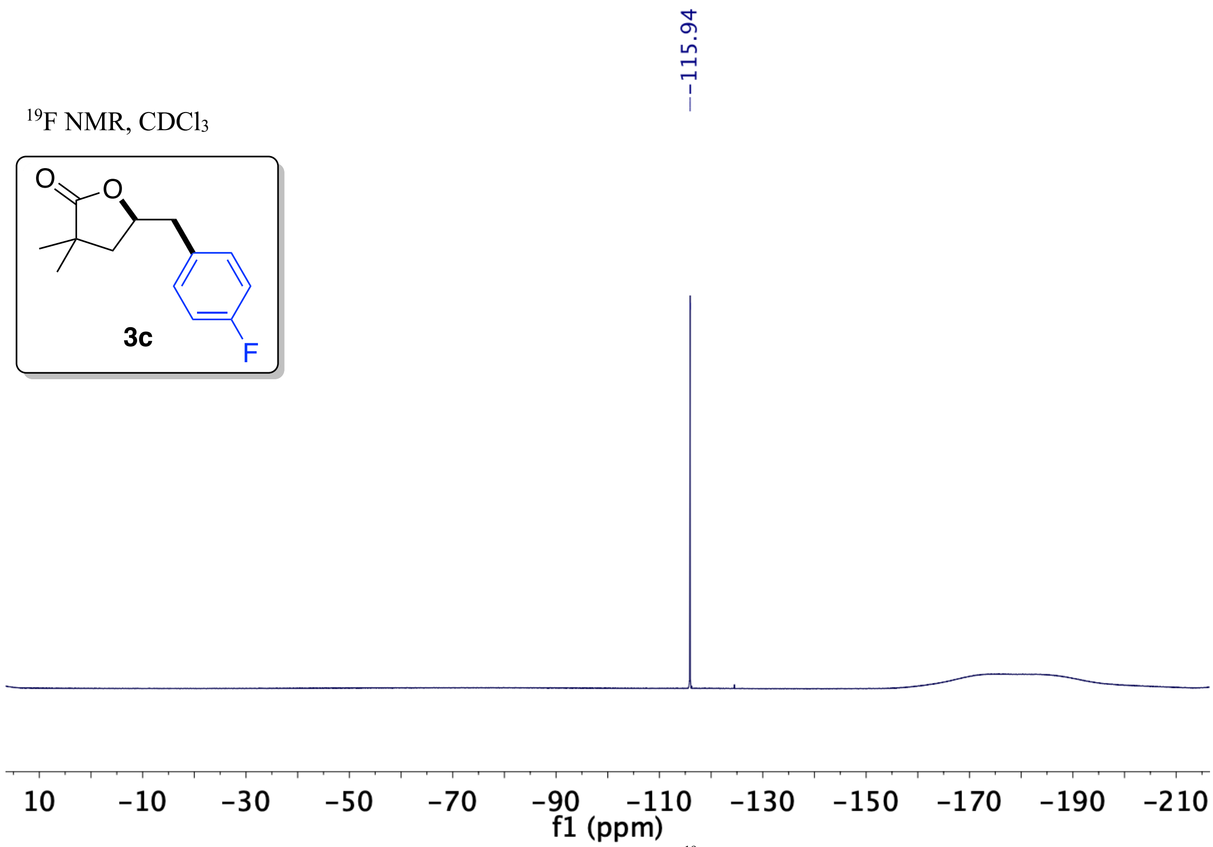

Supplementary Fig. 13. ^{19}F NMR Spectrum for **3c**

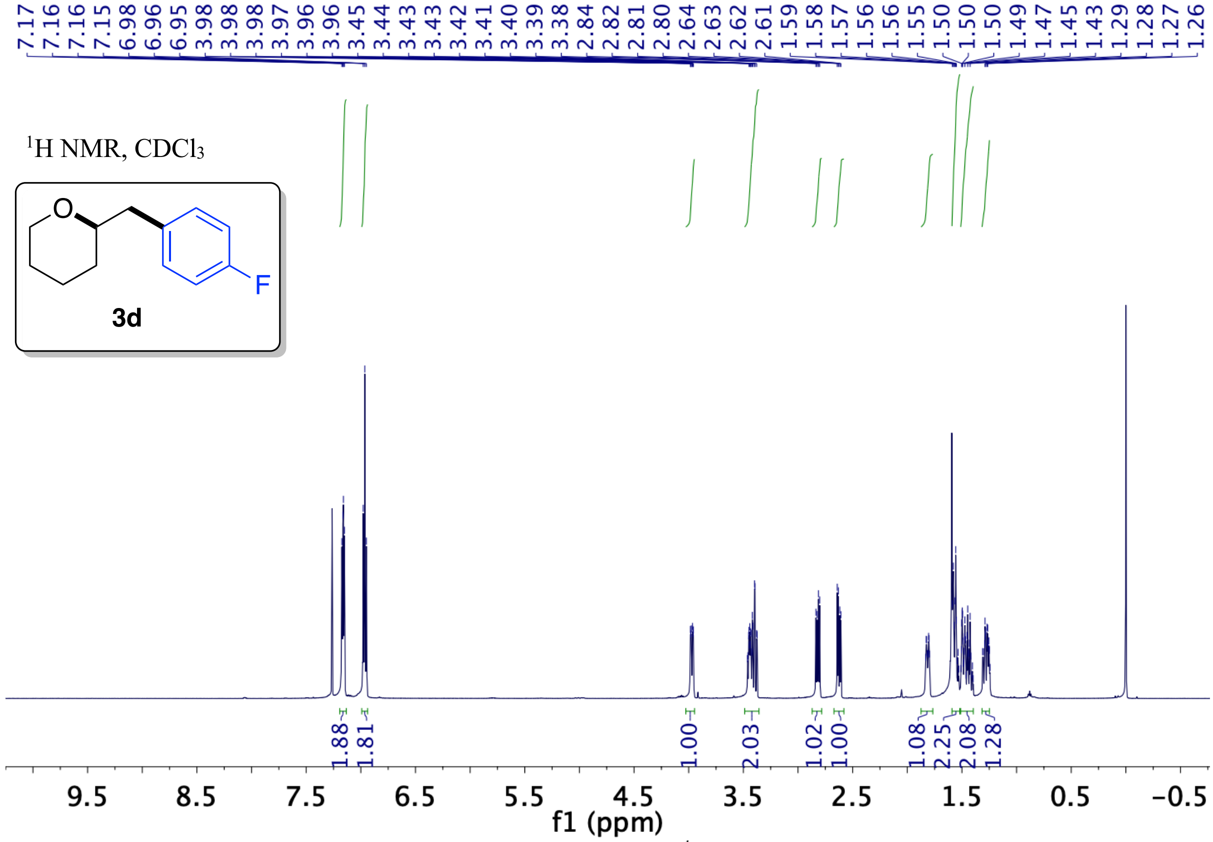

Supplementary Fig. 14. ^{1}H NMR Spectrum for **3d**

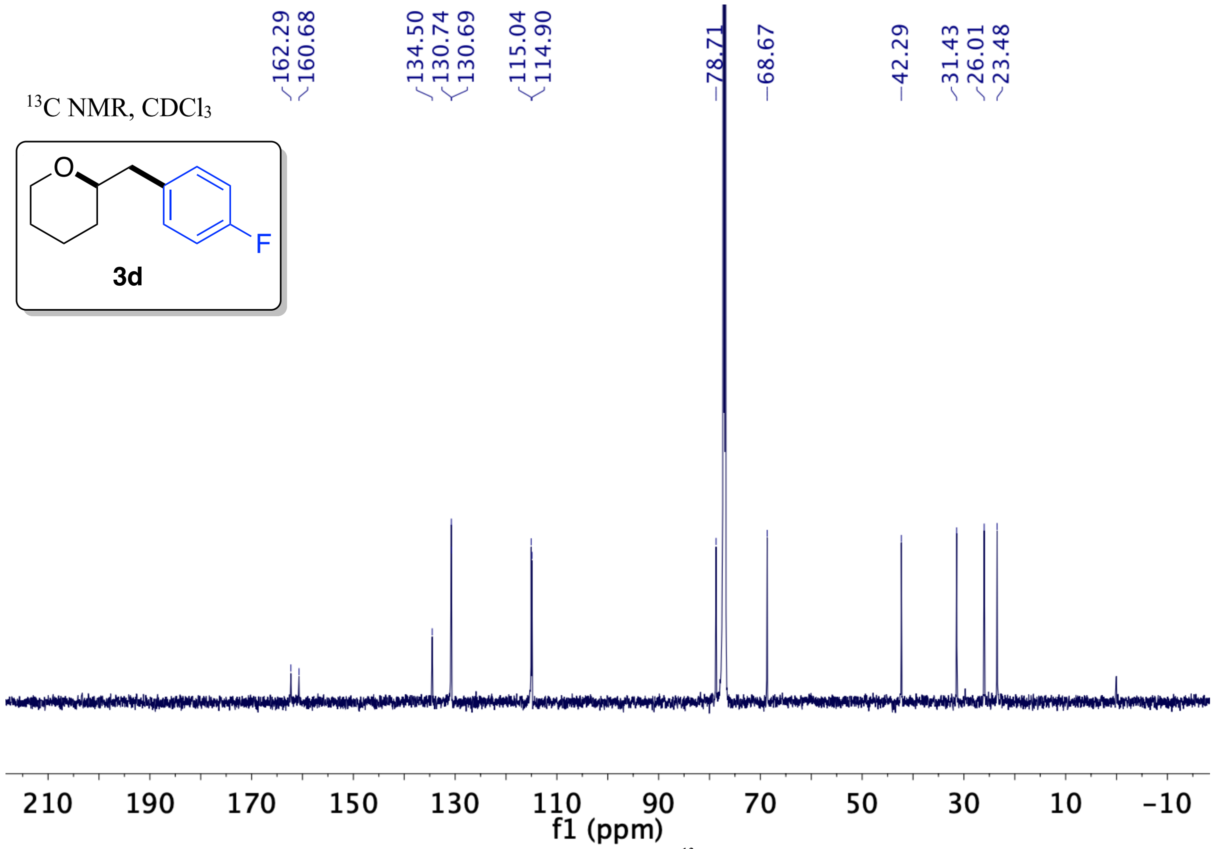

Supplementary Fig. 15. ^{13}C NMR Spectrum for **3d**

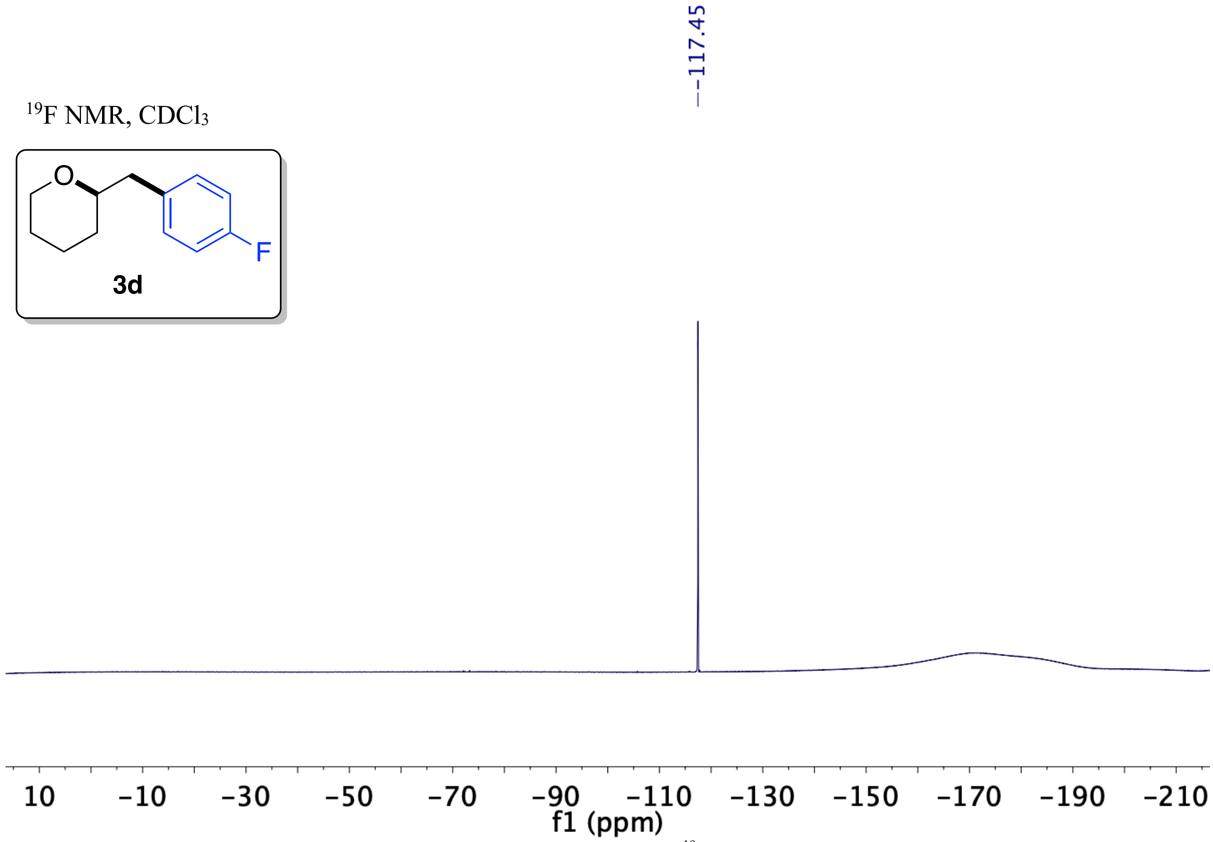

Supplementary Fig. 16. ^{19}F NMR Spectrum for **3d**

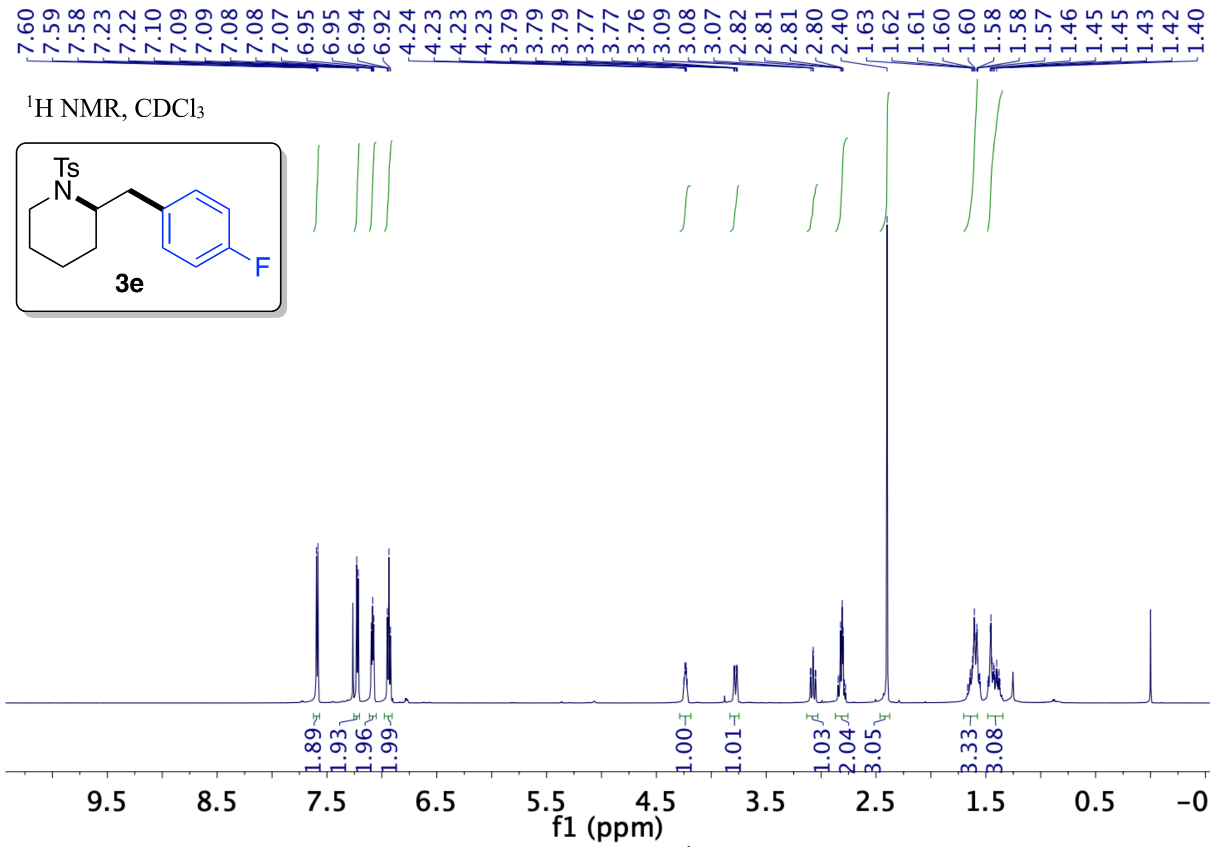

Supplementary Fig. 17. ^{1}H NMR Spectrum for **3e**

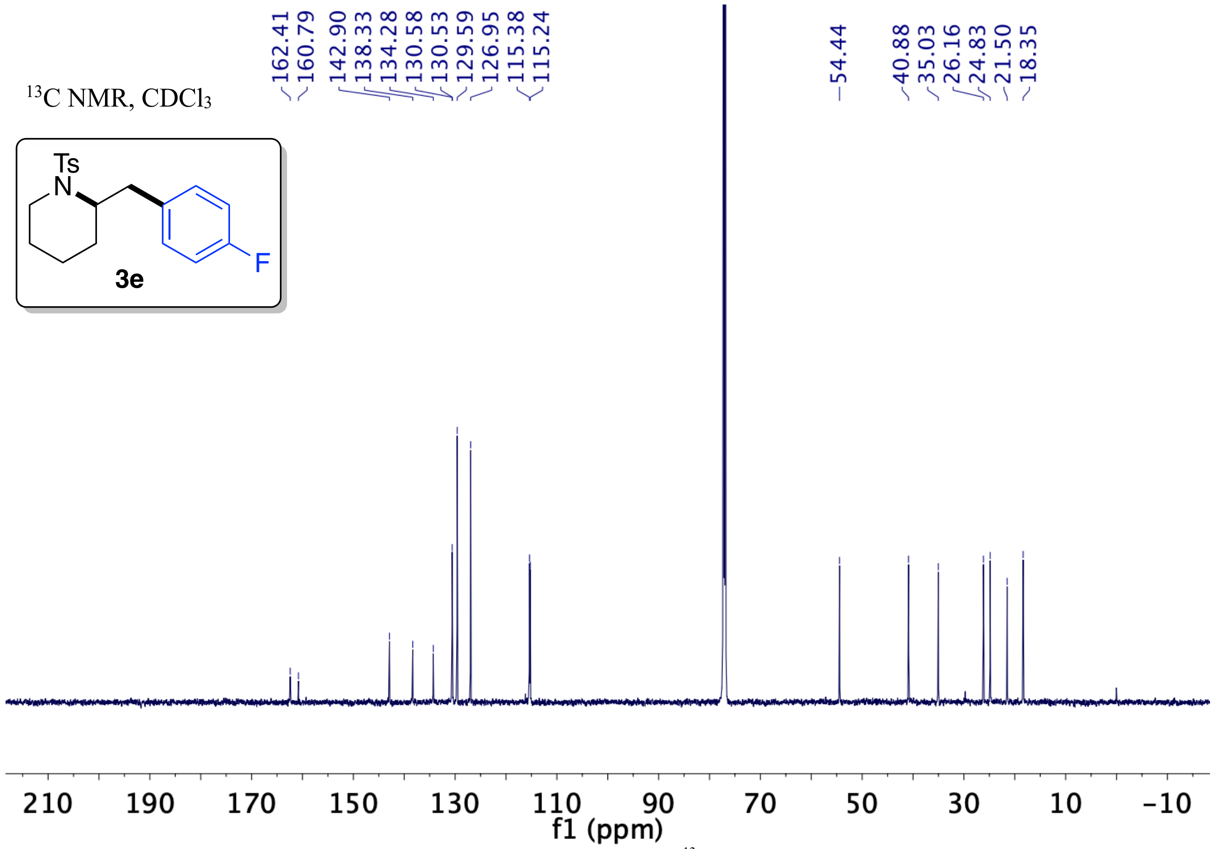

Supplementary Fig. 18. ^{13}C NMR Spectrum for **3e**

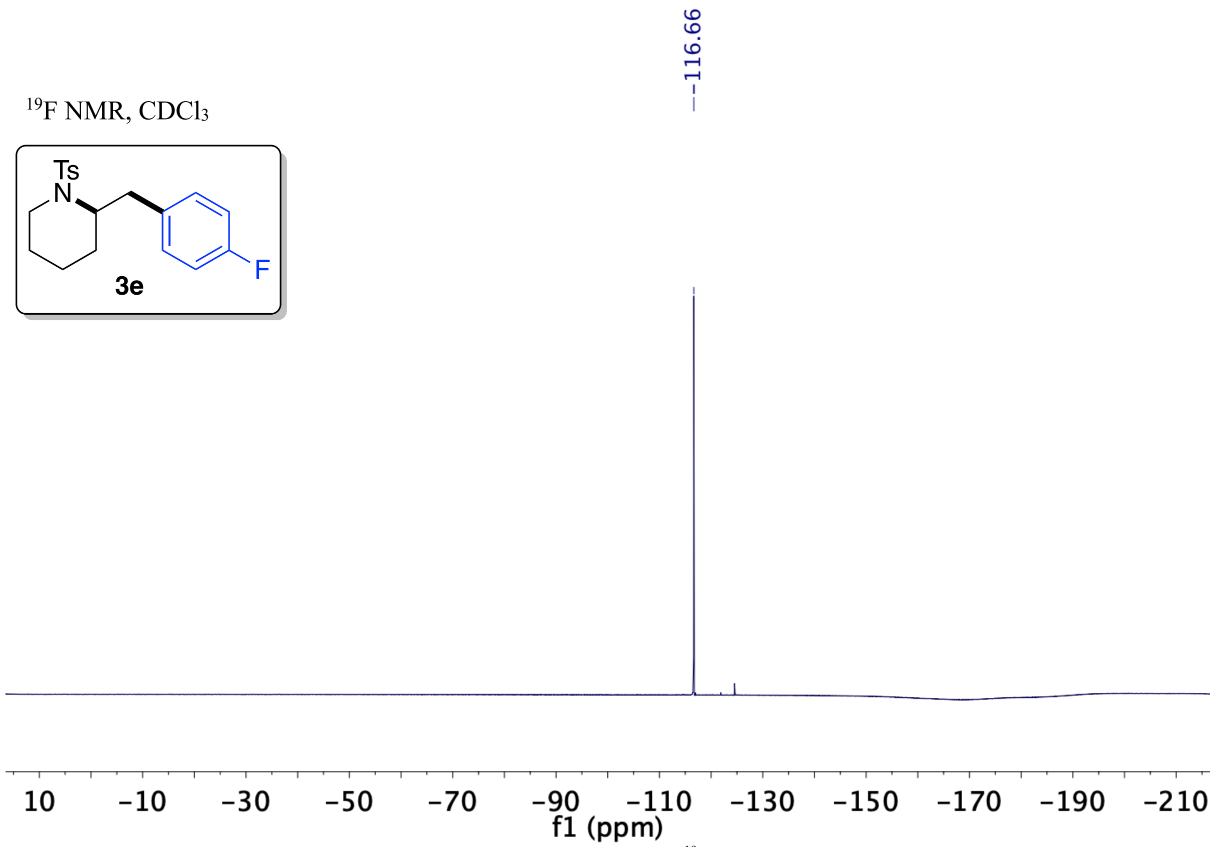

Supplementary Fig. 19. ^{19}F NMR Spectrum for **3e**

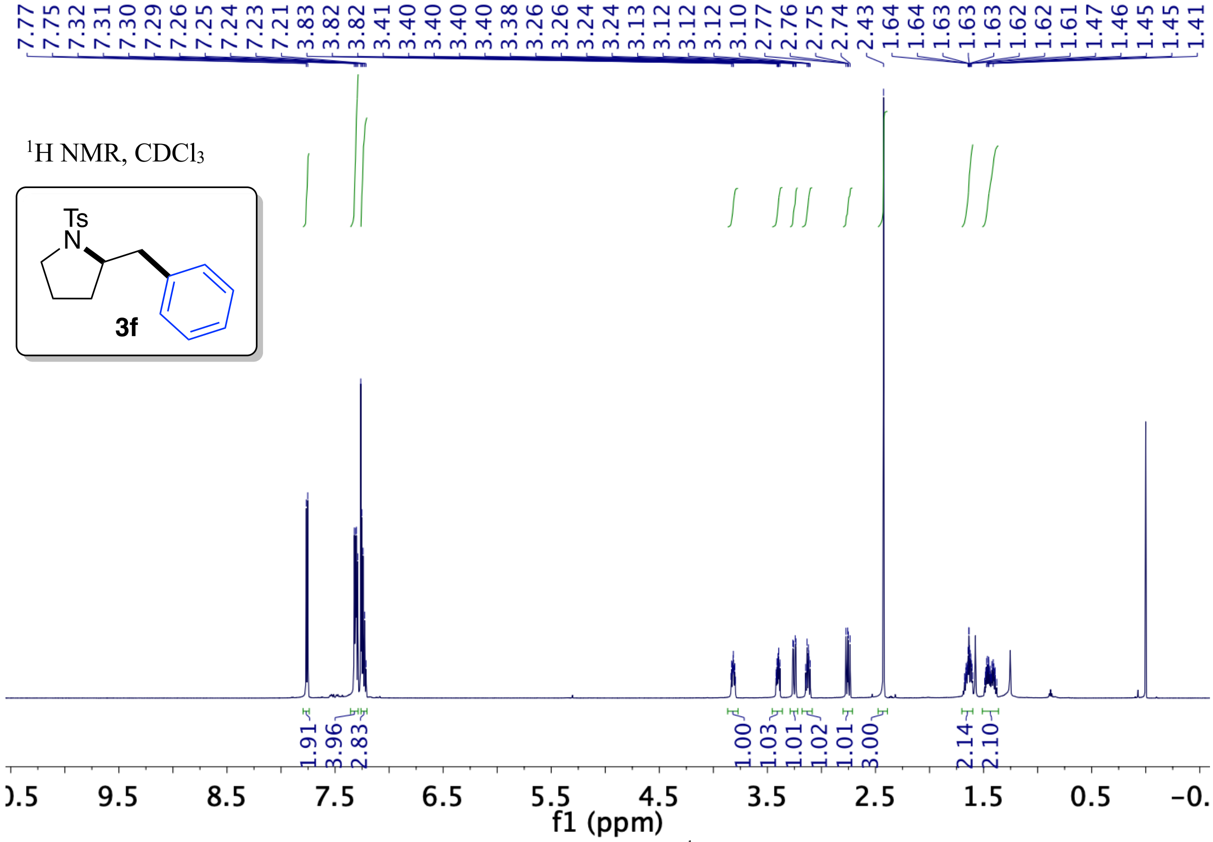

Supplementary Fig. 20. ^{1}H NMR Spectrum for **3f**

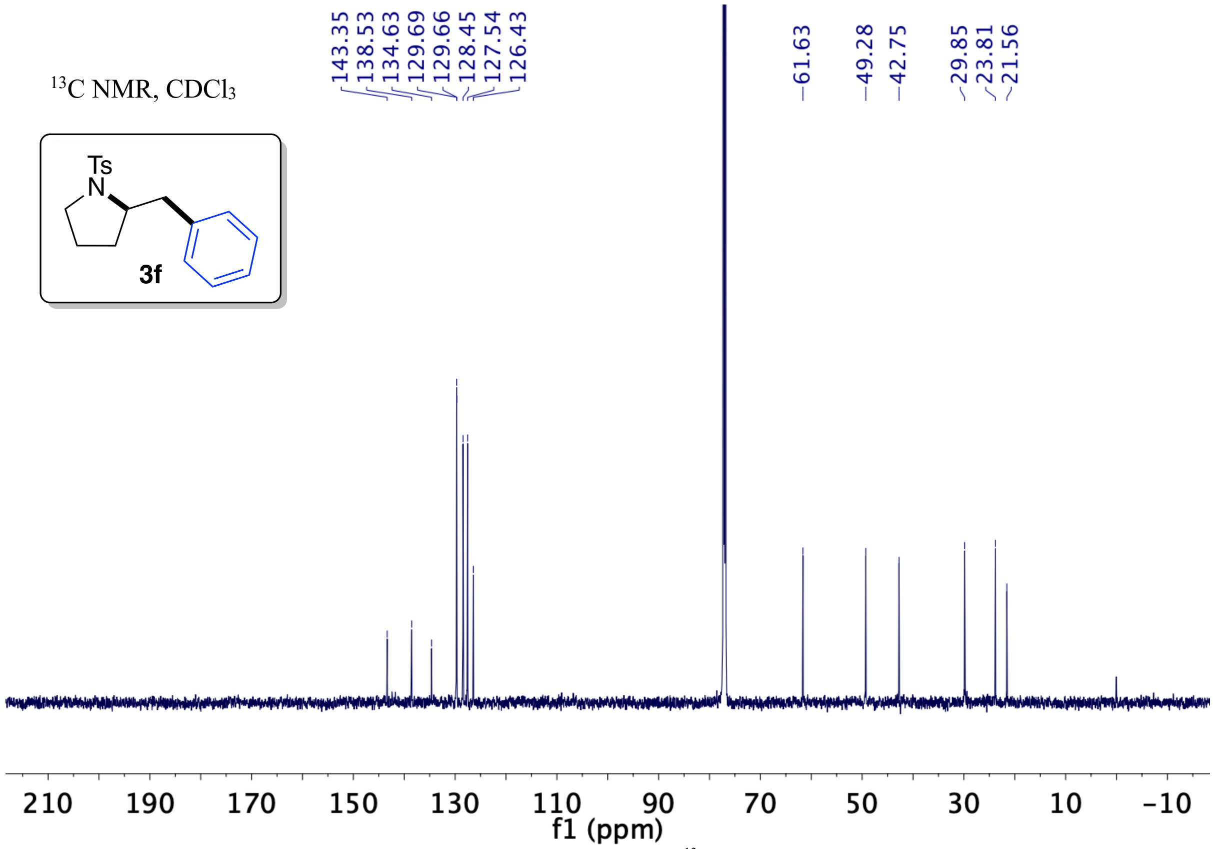

Supplementary Fig. 21. ^{13}C NMR Spectrum for **3f**

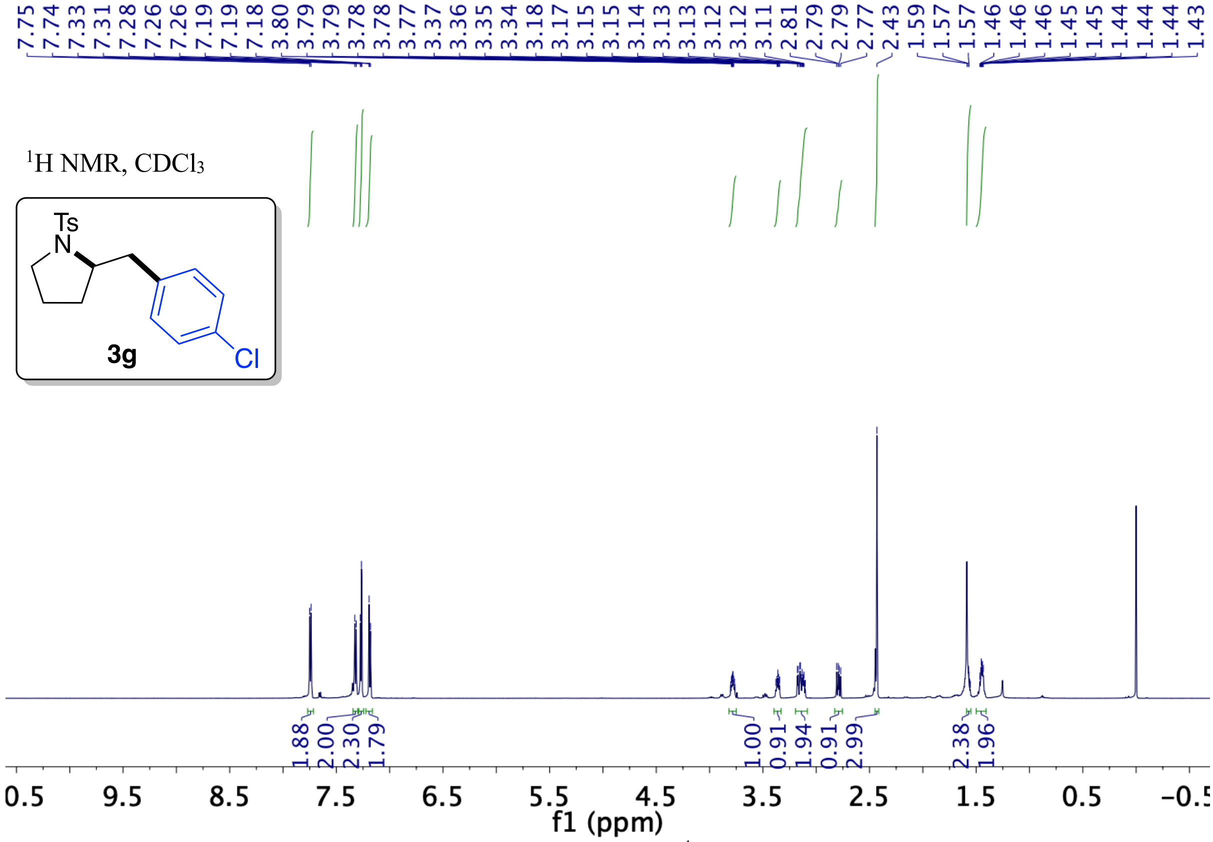

Supplementary Fig. 22. ^{1}H NMR Spectrum for **3g**

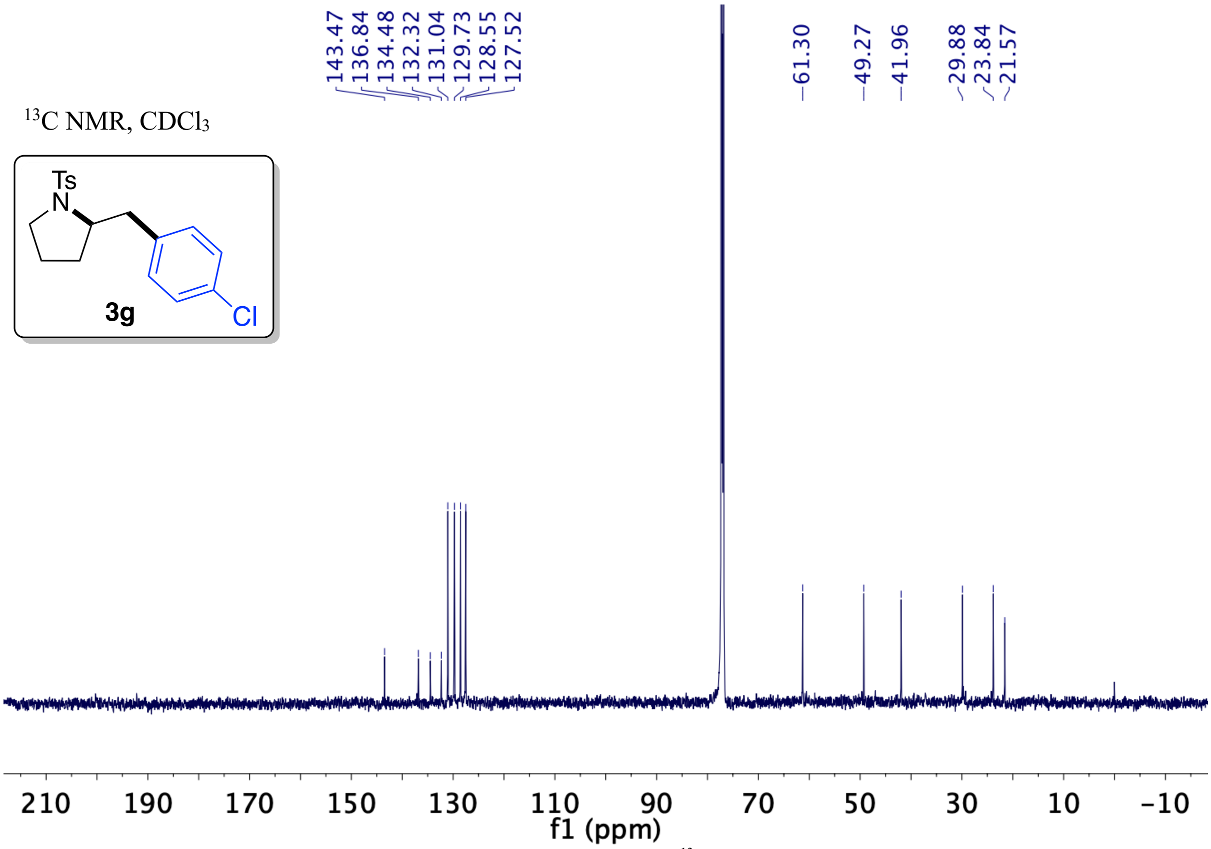

Supplementary Fig. 23. ^{13}C NMR Spectrum for **3g**

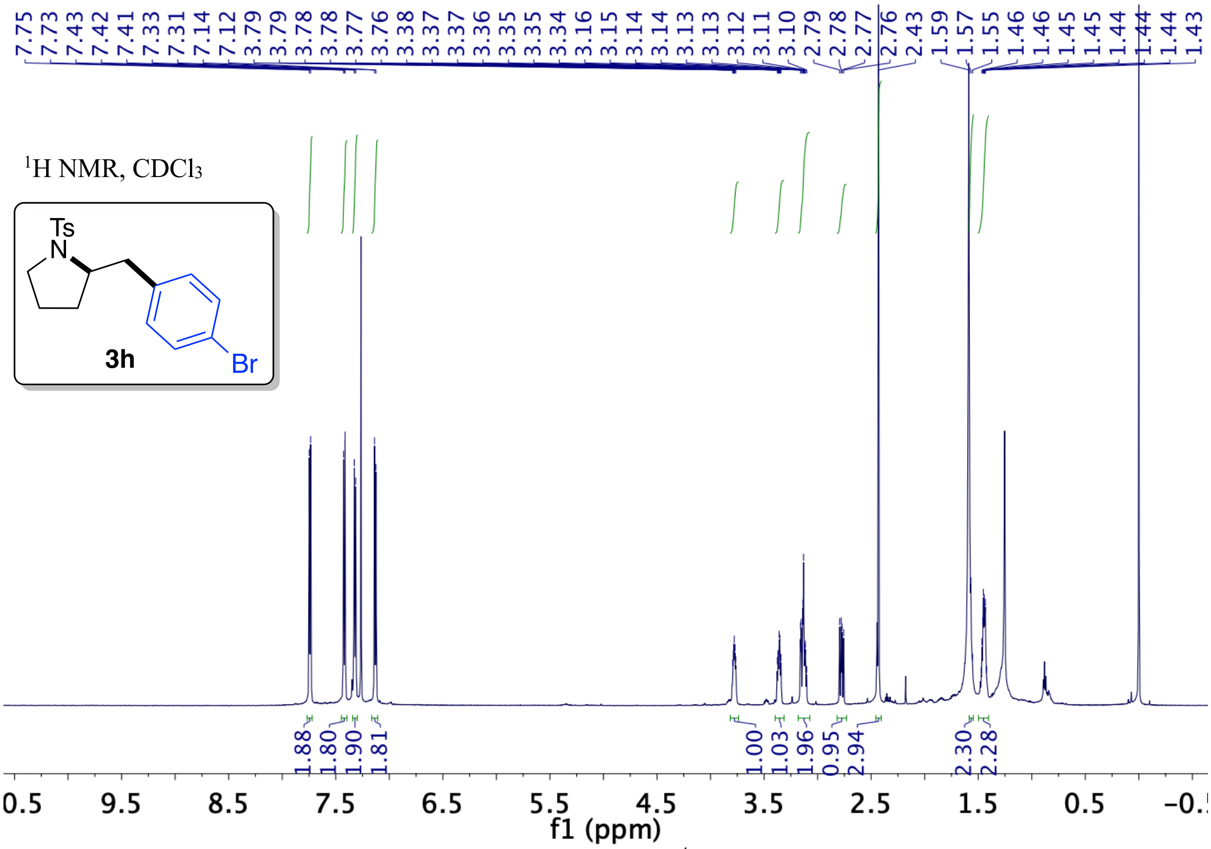

Supplementary Fig. 24. ^{1}H NMR Spectrum for **3h**

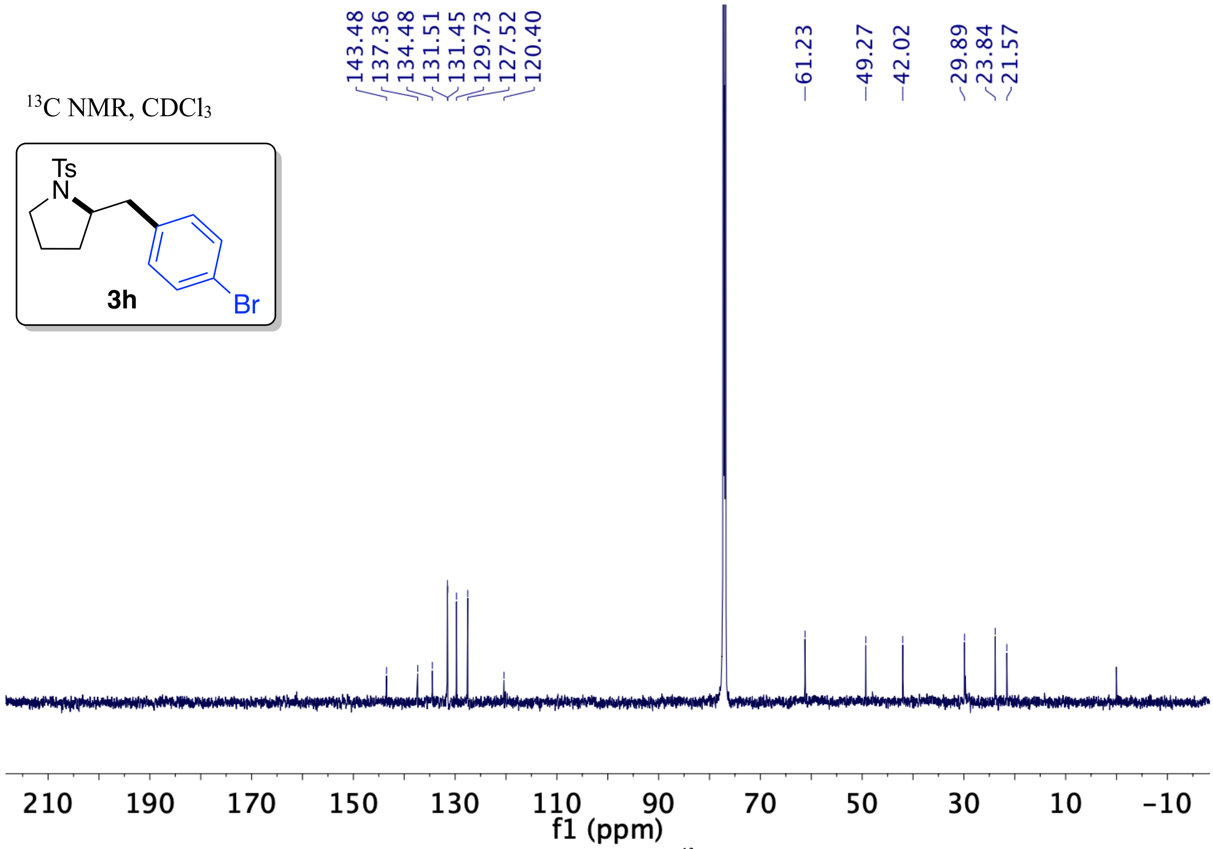

Supplementary Fig. 25. ^{13}C NMR Spectrum for **3h**

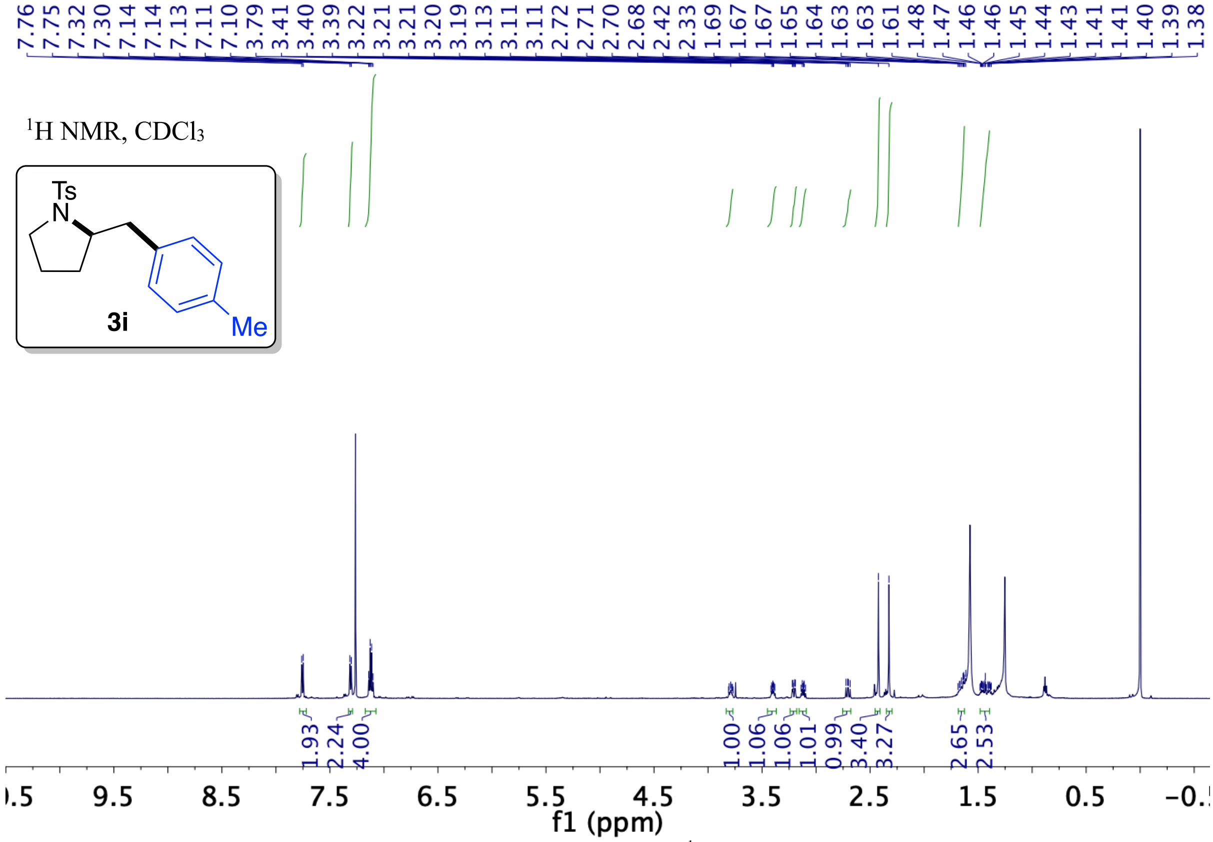

Supplementary Fig. 26. ^{1}H NMR Spectrum for **3i**

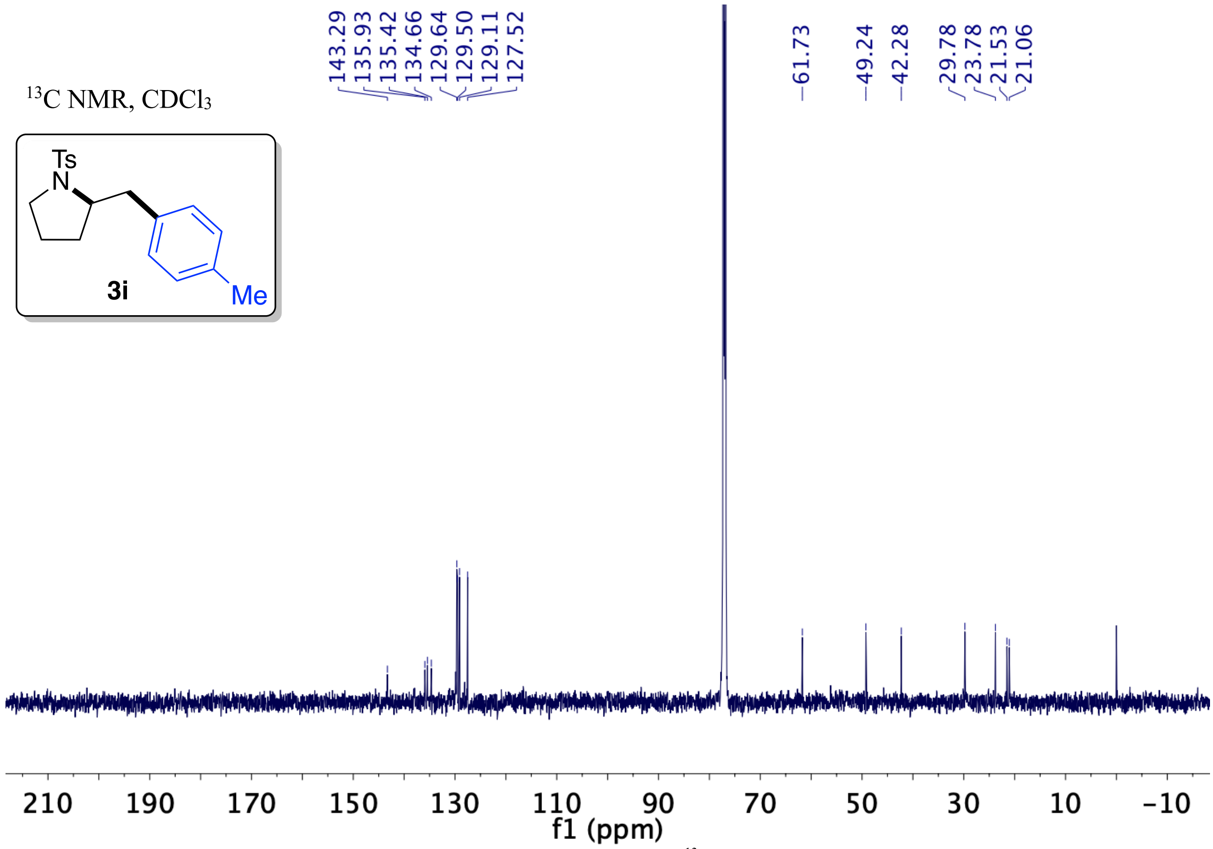

Supplementary Fig. 27. ^{13}C NMR Spectrum for **3i**

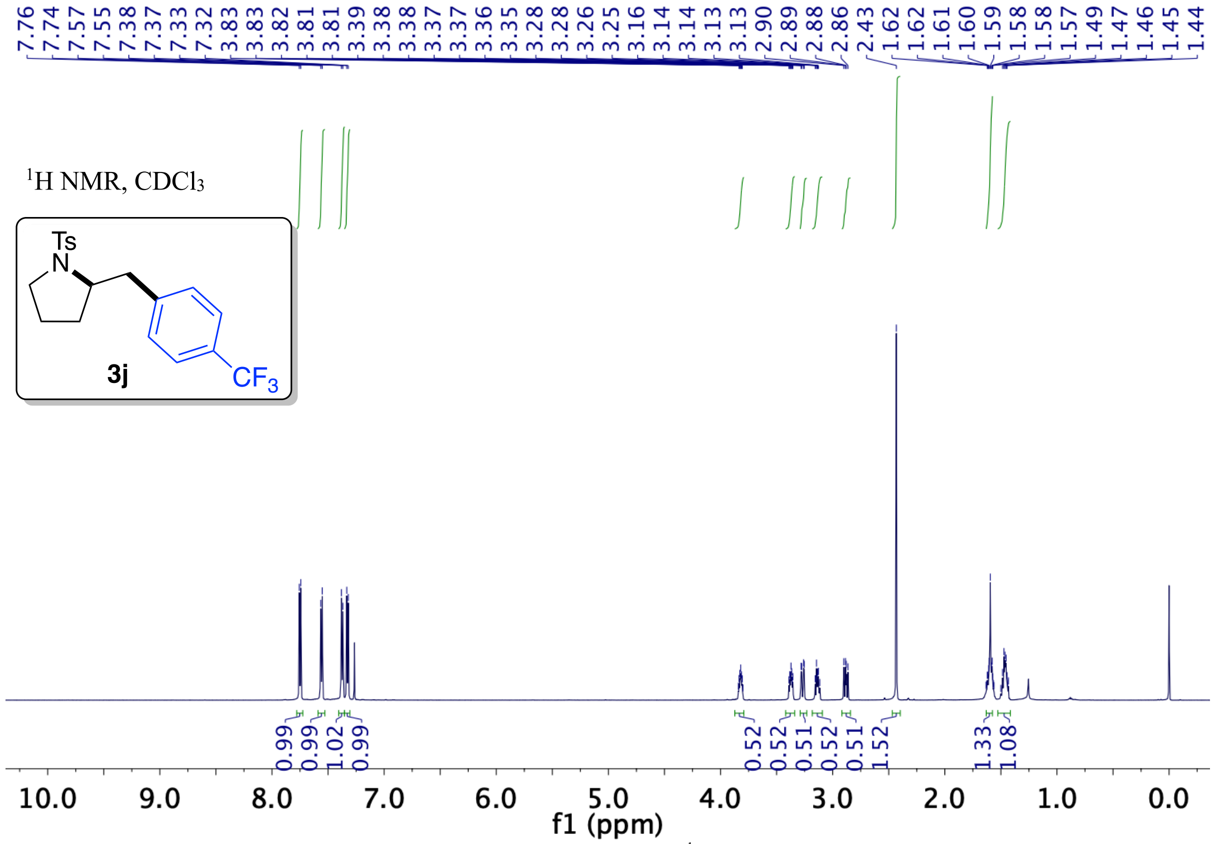

Supplementary Fig. 28. ^{1}H NMR Spectrum for **3j**

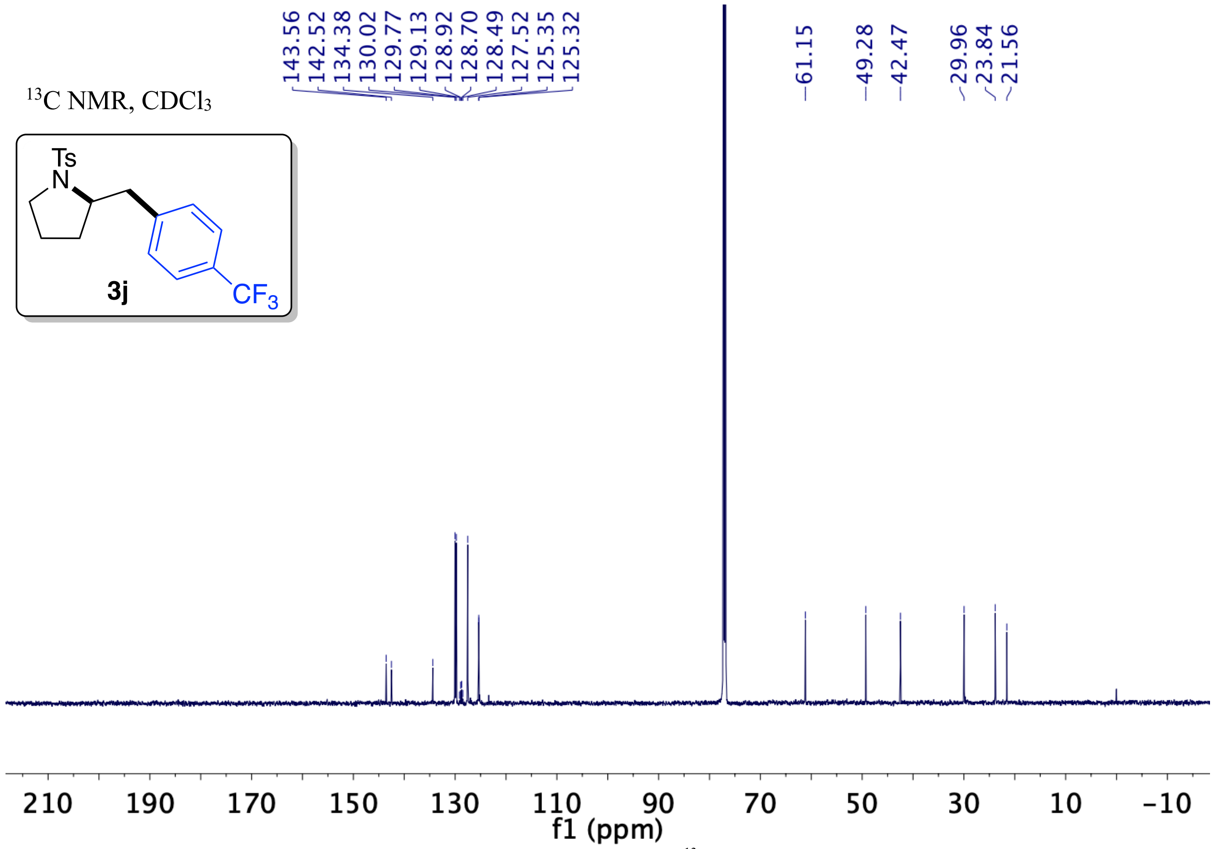

Supplementary Fig. 29. ^{13}C NMR Spectrum for **3j**

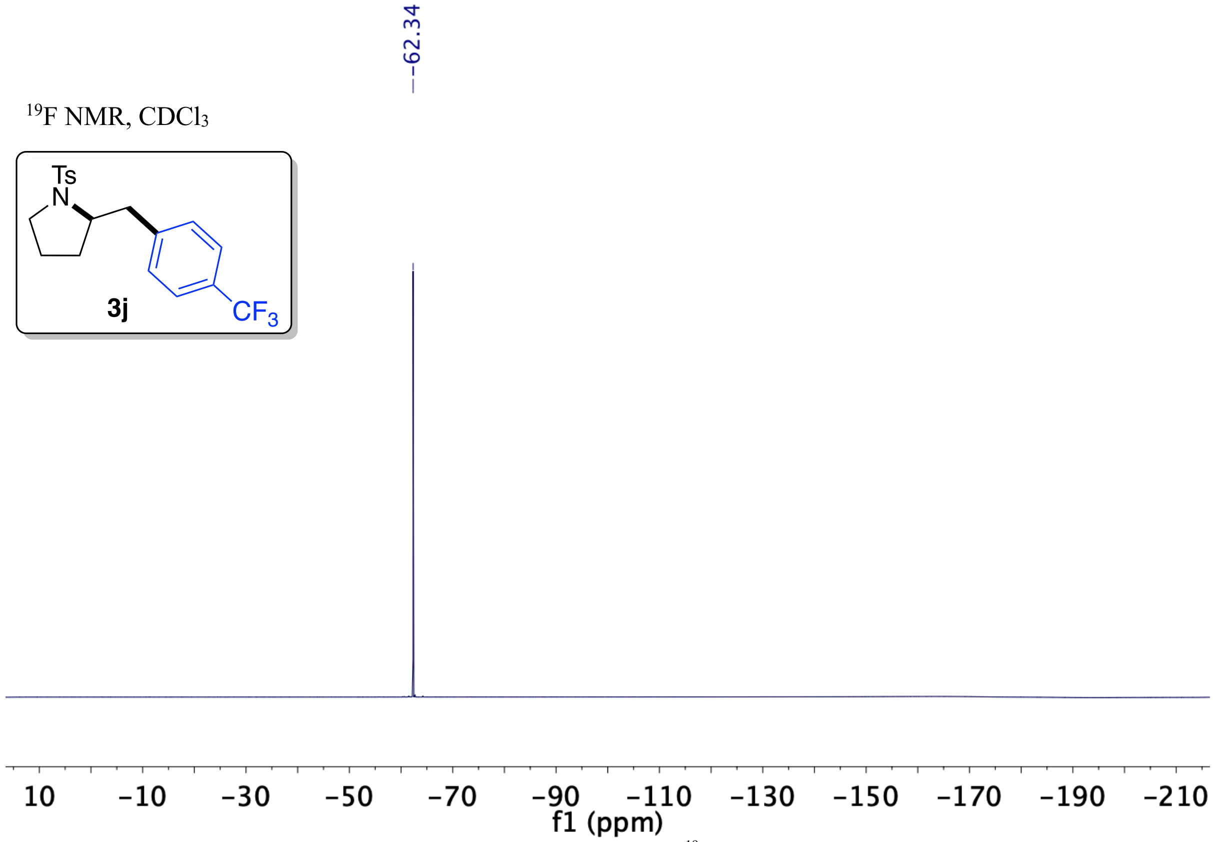

Supplementary Fig. 30. ^{19}F NMR Spectrum for **3j**

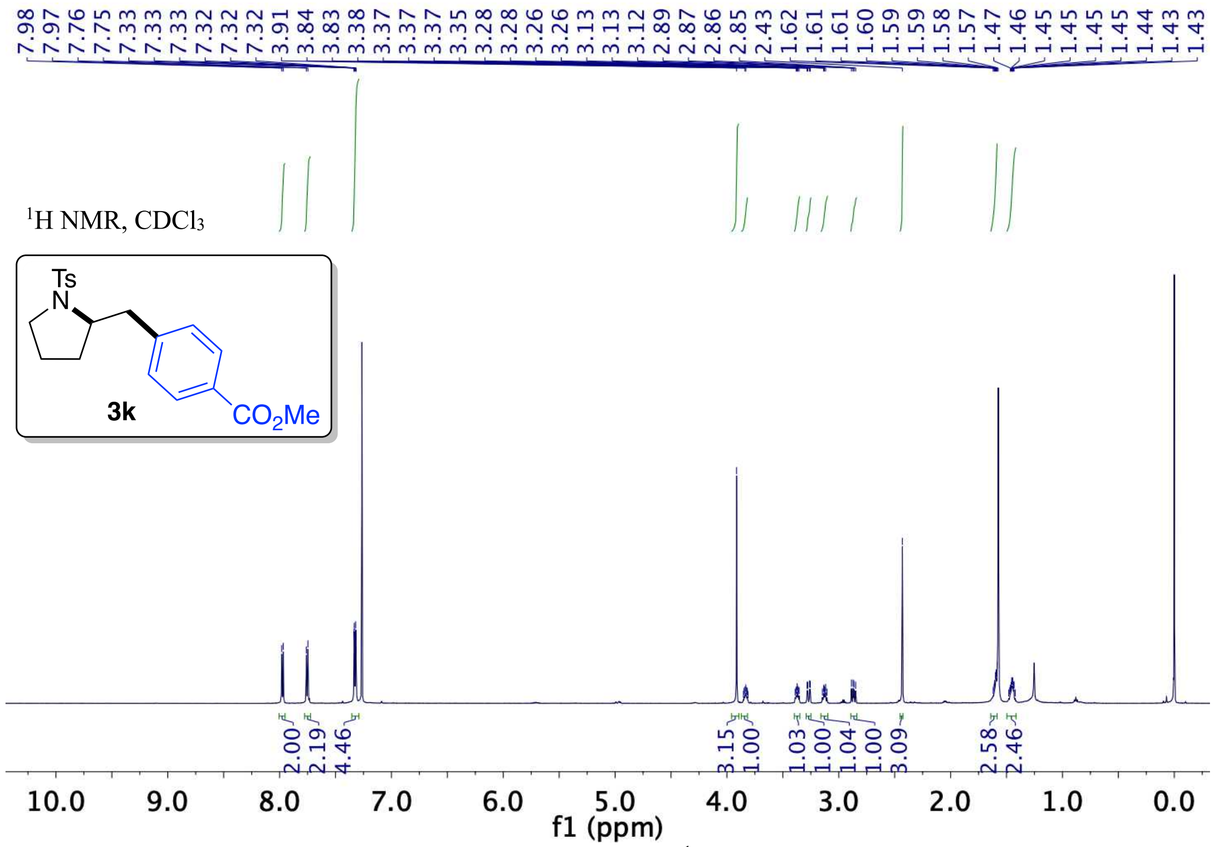

Supplementary Fig. 31. ^{1}H NMR Spectrum for **3k**

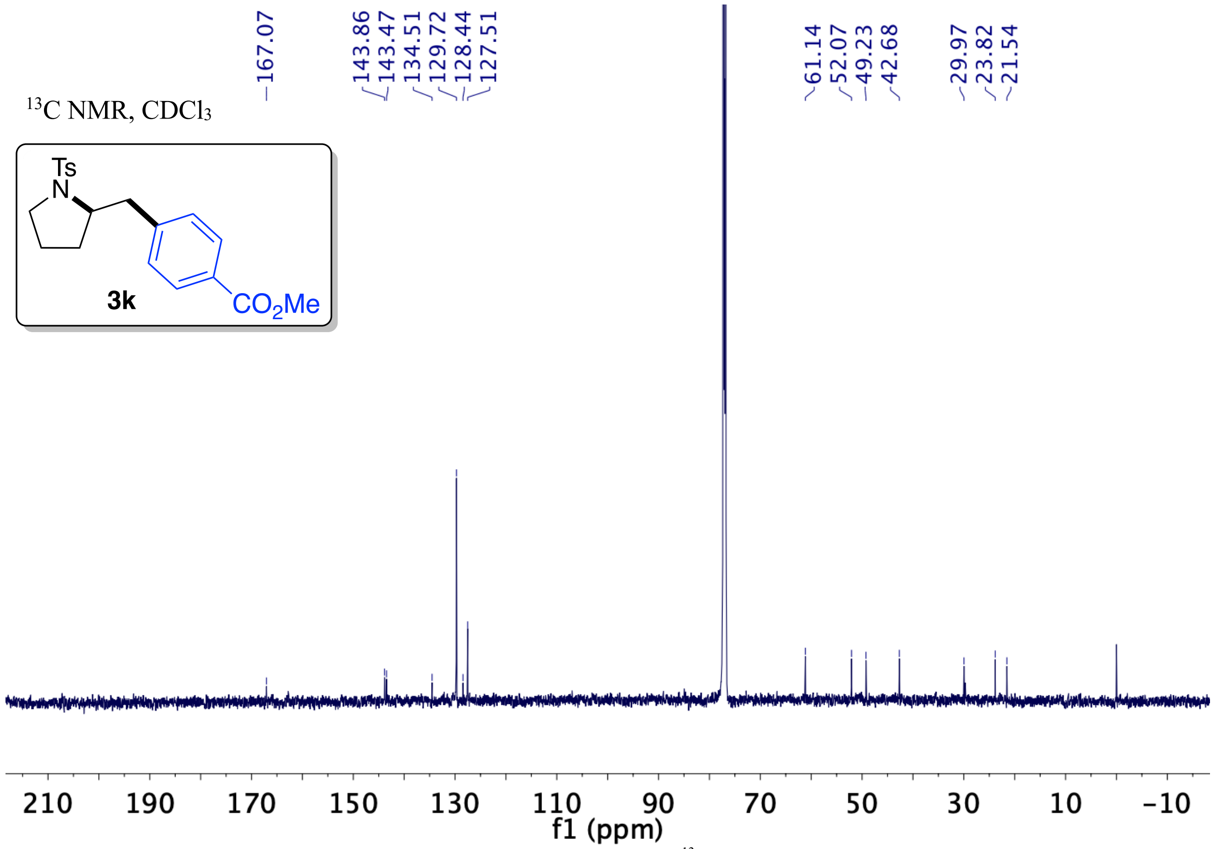

Supplementary Fig. 32. ^{13}C NMR Spectrum for **3k**

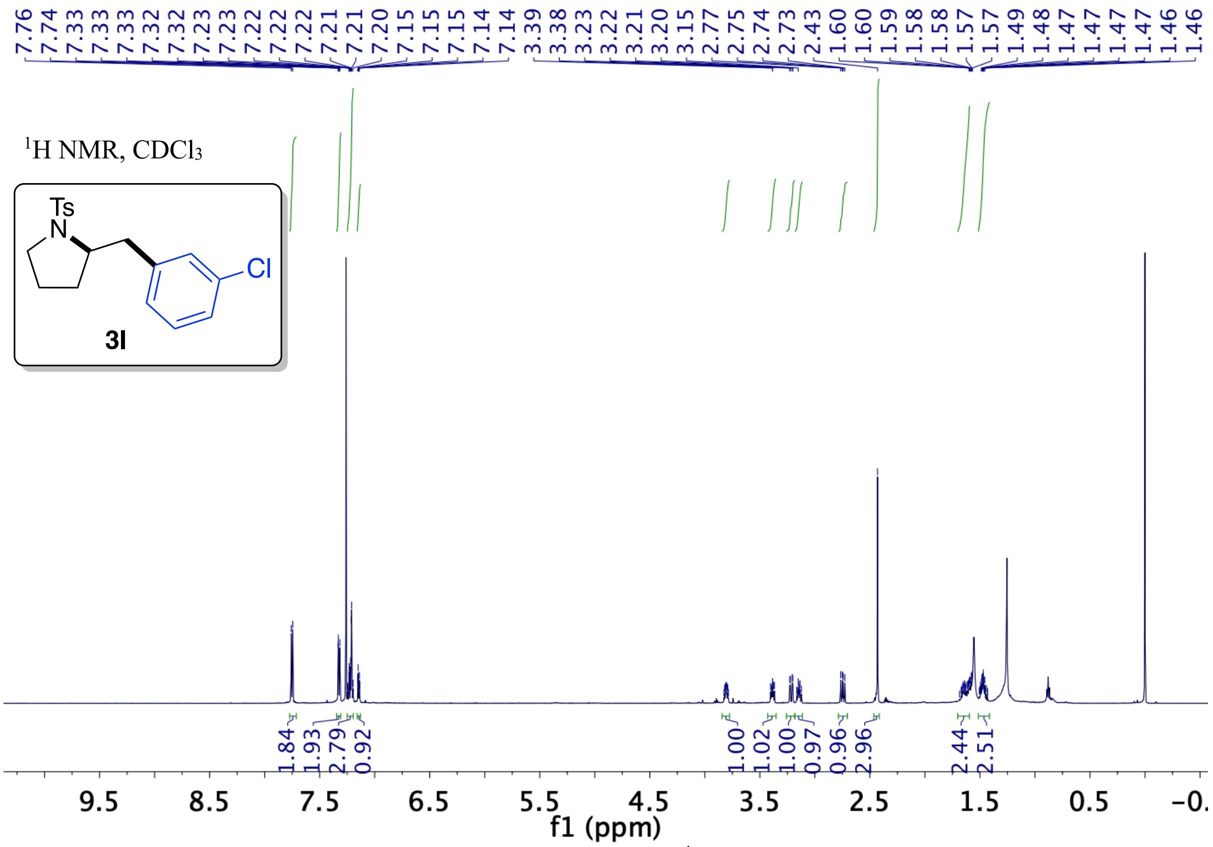

Supplementary Fig. 33. ^{1}H NMR Spectrum for **3l**

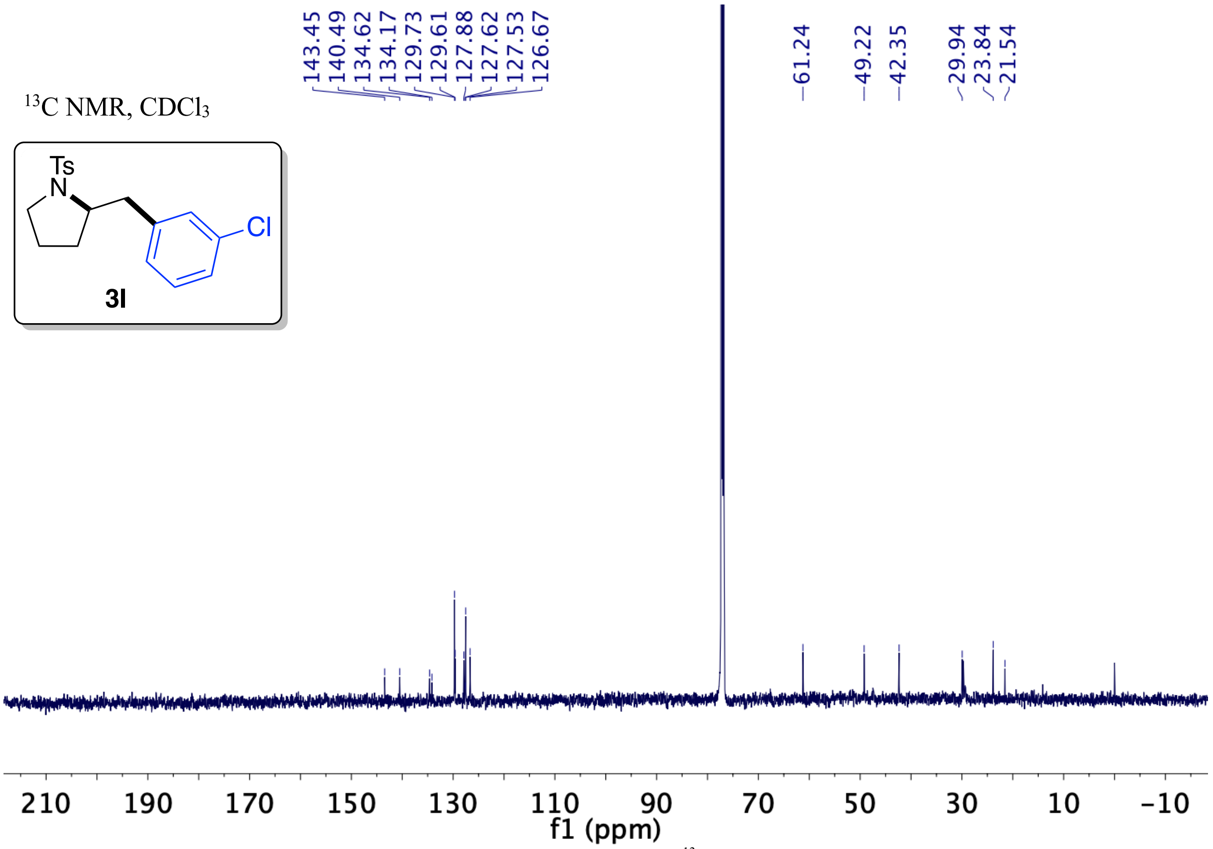

Supplementary Fig. 34. ^{13}C NMR Spectrum for **3l**

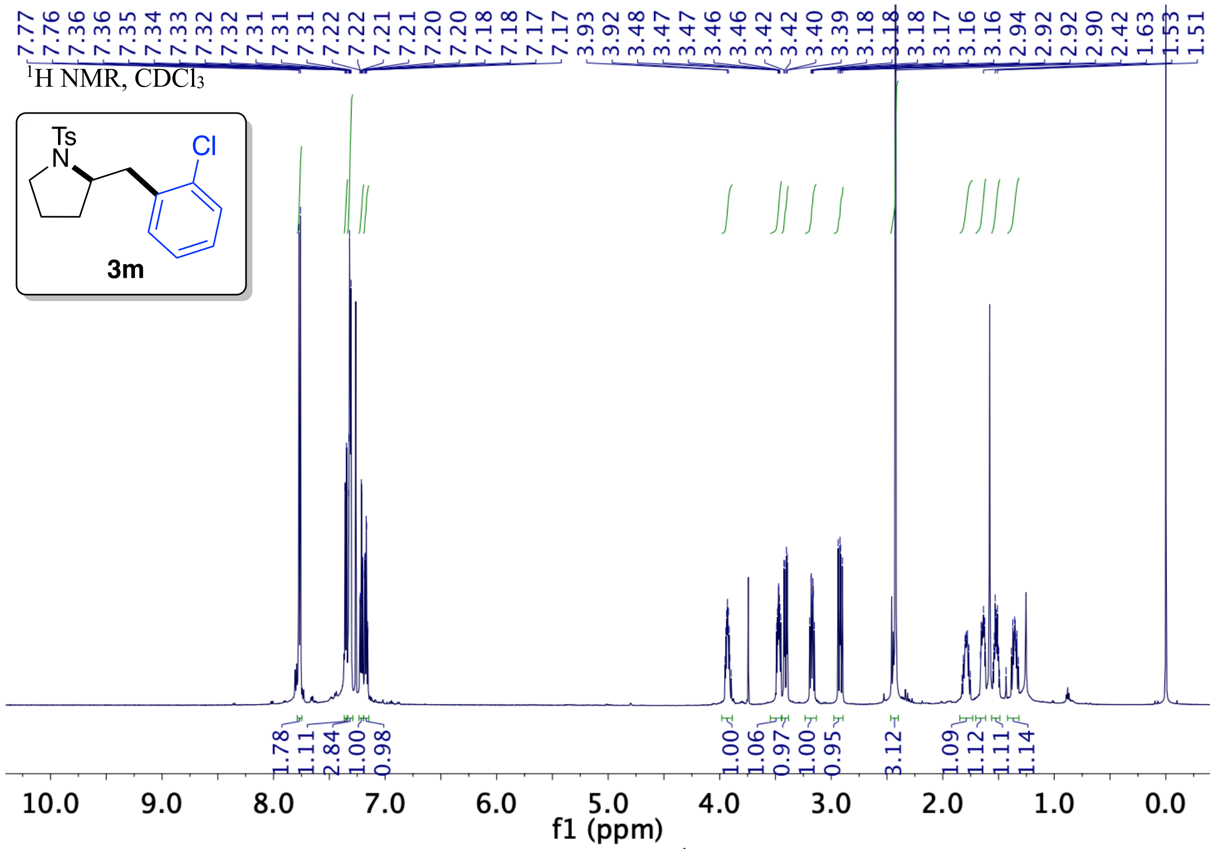

Supplementary Fig. 35. ^{1}H NMR Spectrum for **3m**

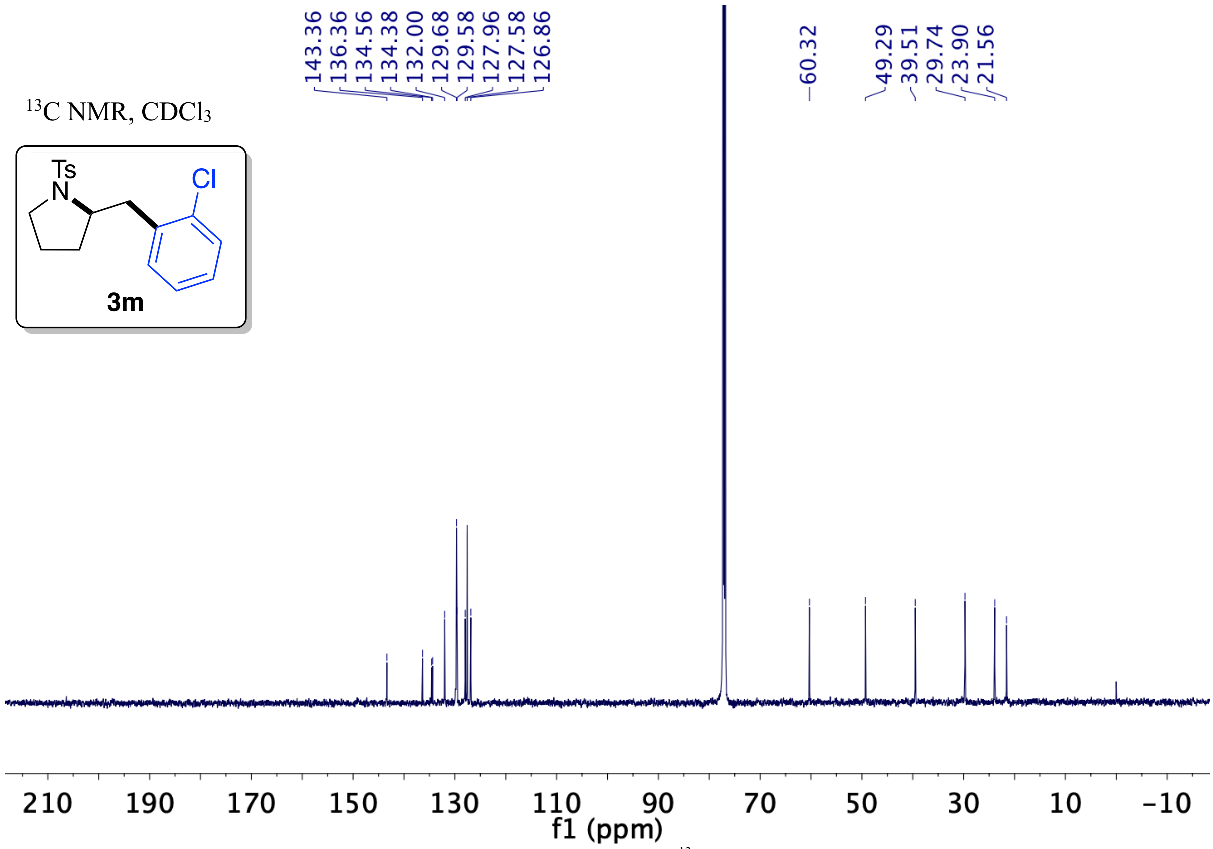

Supplementary Fig. 36. ^{13}C NMR Spectrum for **3m**

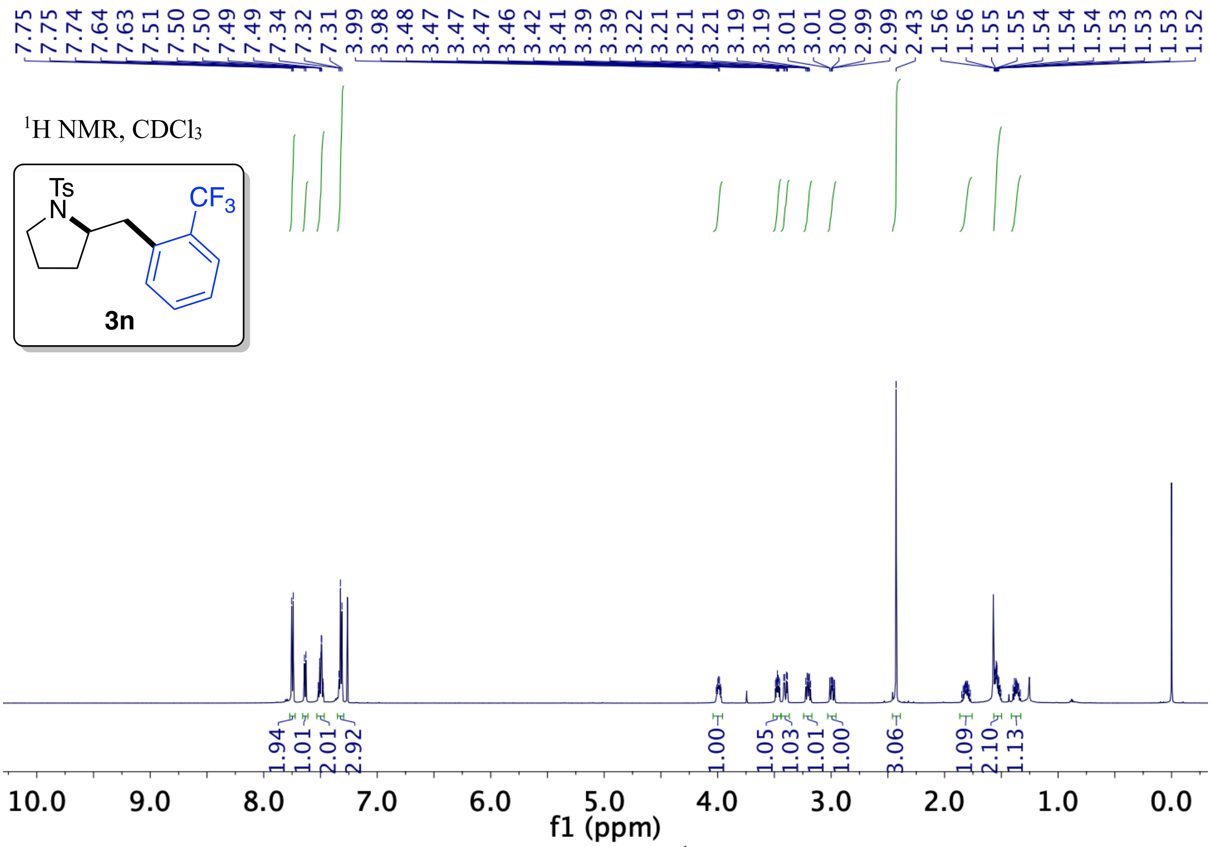

Supplementary Fig. 37. ^{1}H NMR Spectrum for **3n**

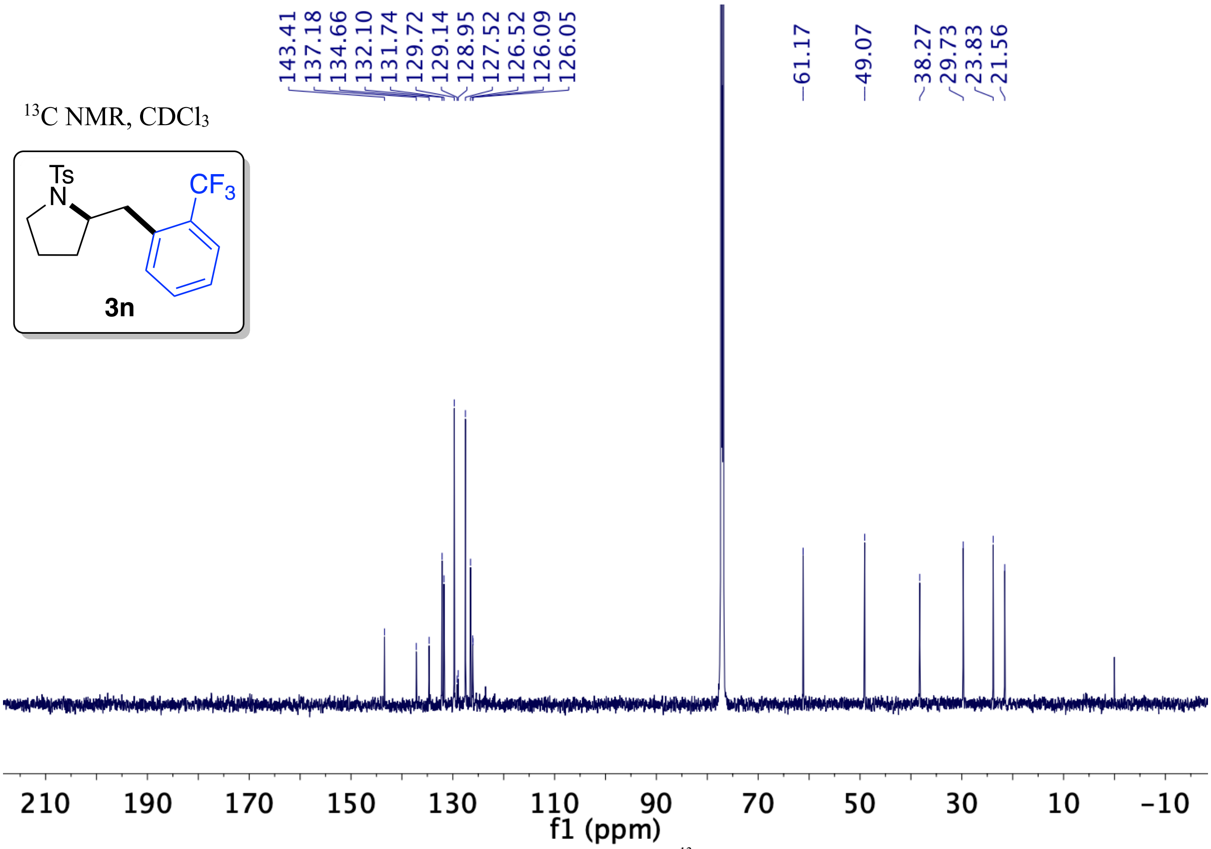

Supplementary Fig. 38. ^{13}C NMR Spectrum for **3n**

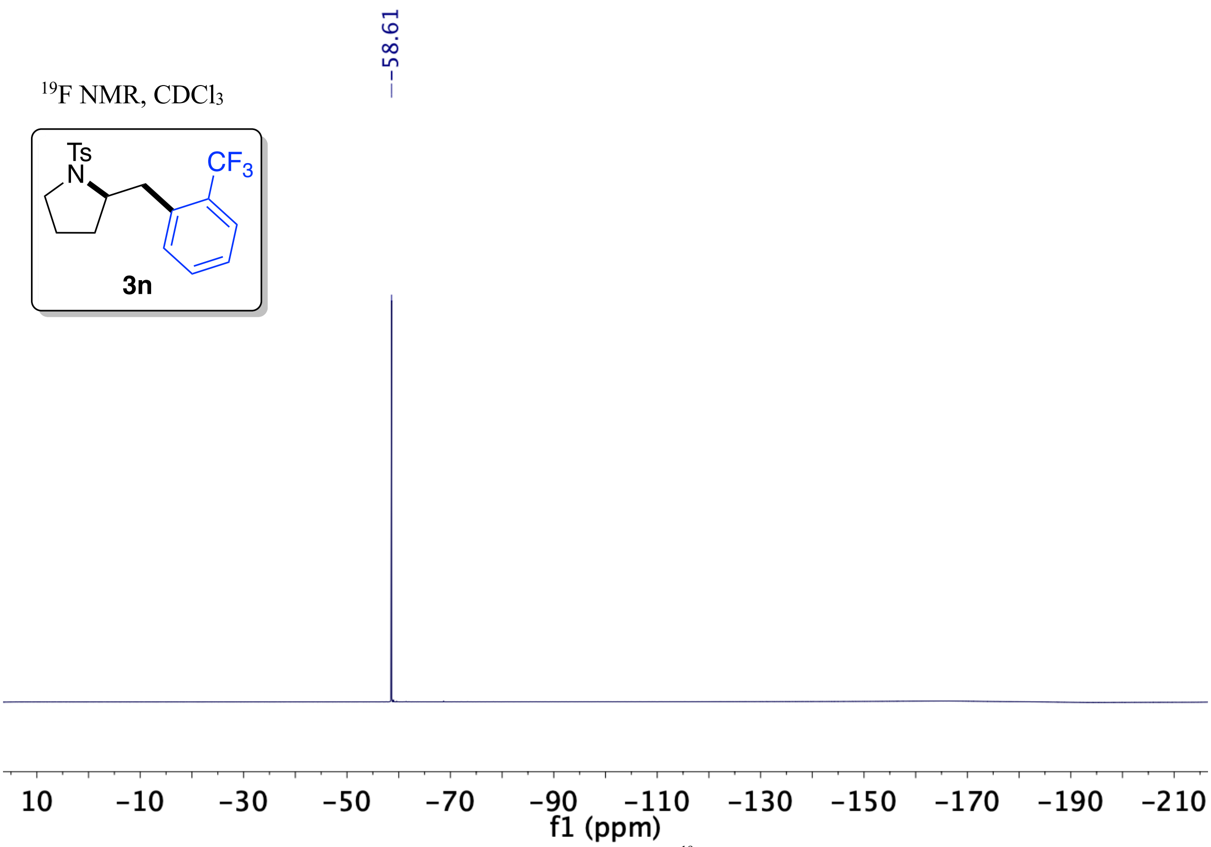

Supplementary Fig. 39. ^{19}F NMR Spectrum for **3n**

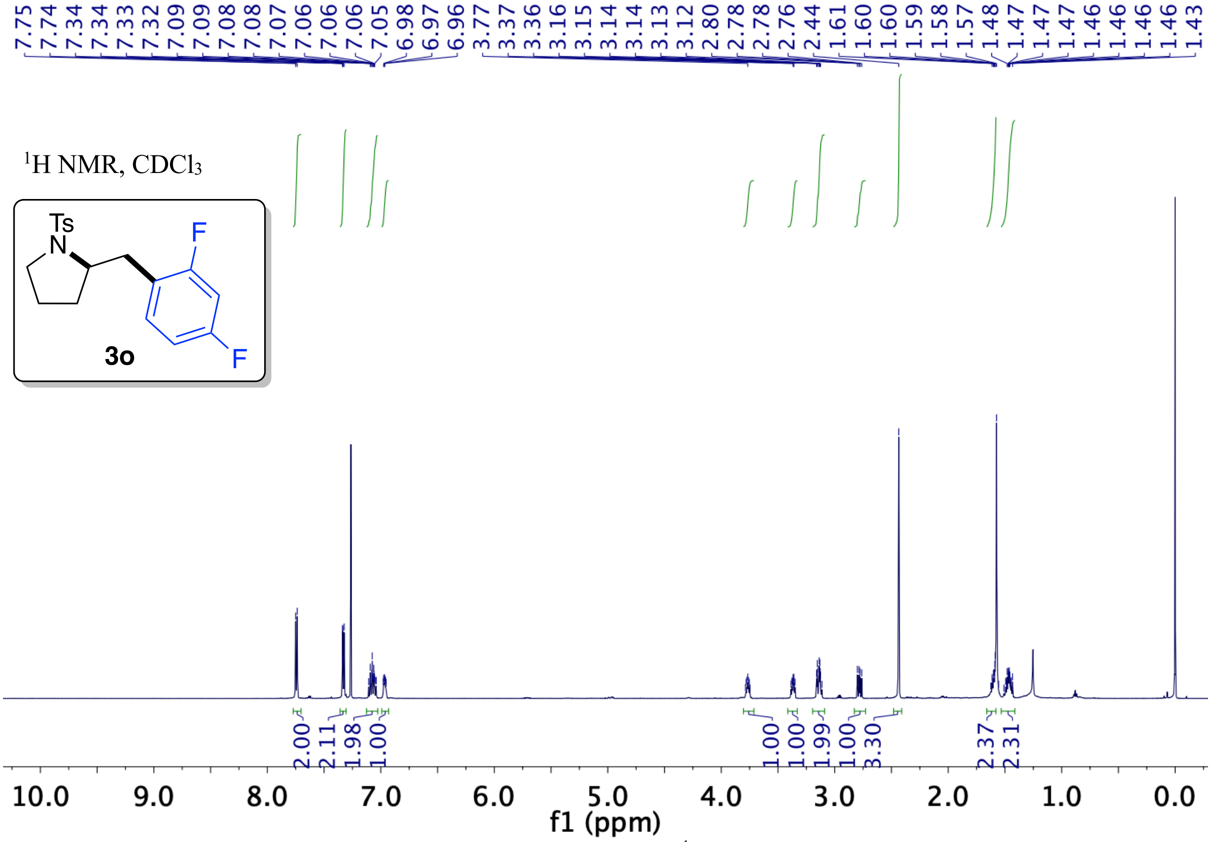

Supplementary Fig. 40. ^{1}H NMR Spectrum for **3o**

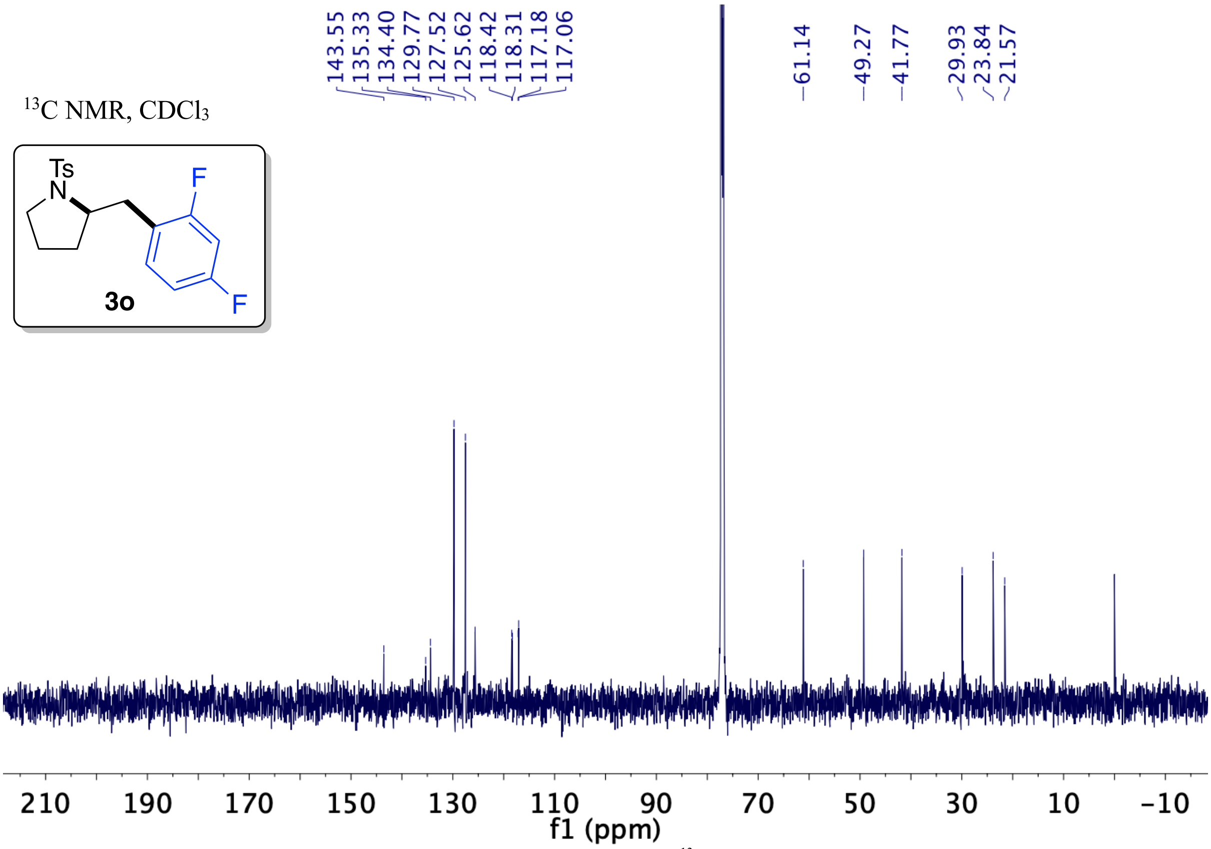

Supplementary Fig. 41. ^{13}C NMR Spectrum for **3o**

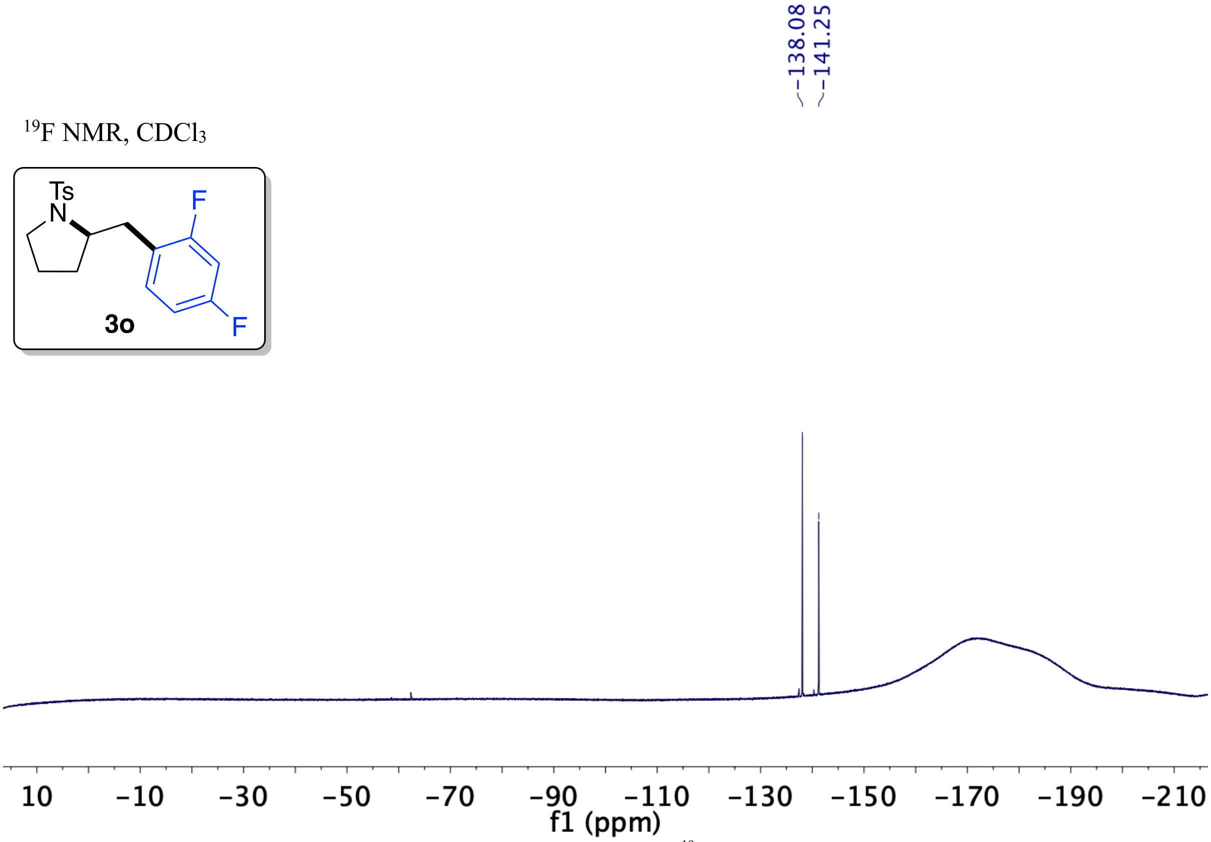

Supplementary Fig. 42. ^{19}F NMR Spectrum for **3o**

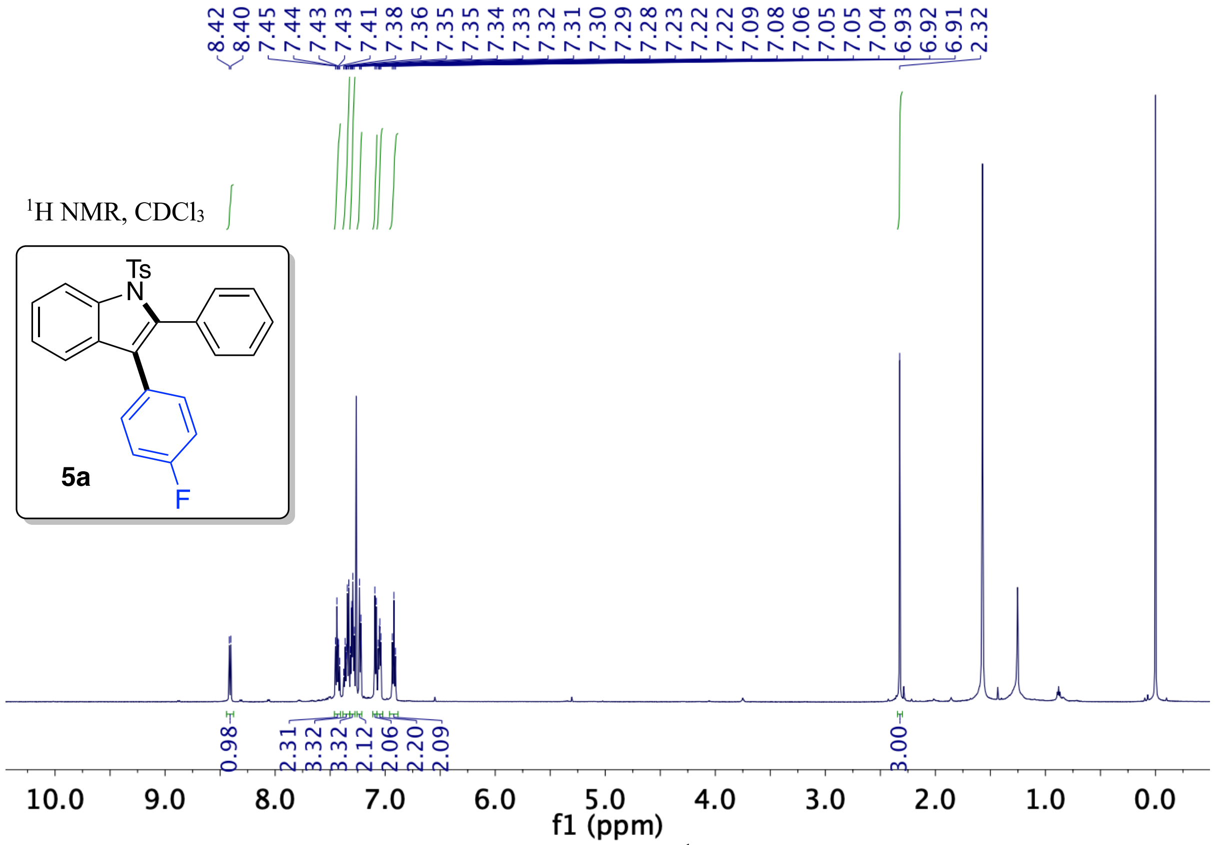

Supplementary Fig. 43. ^{1}H NMR Spectrum for **5a**

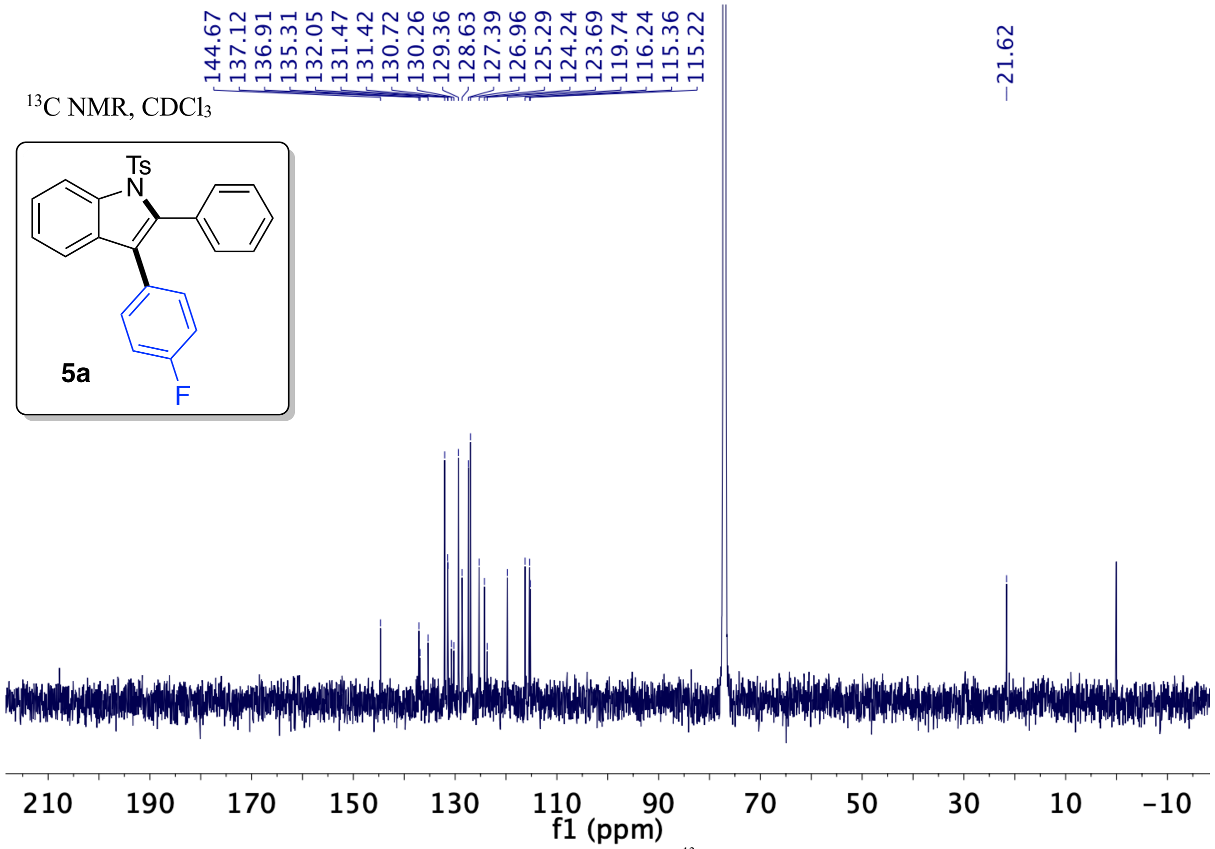

Supplementary Fig. 44. ^{13}C NMR Spectrum for **5a**

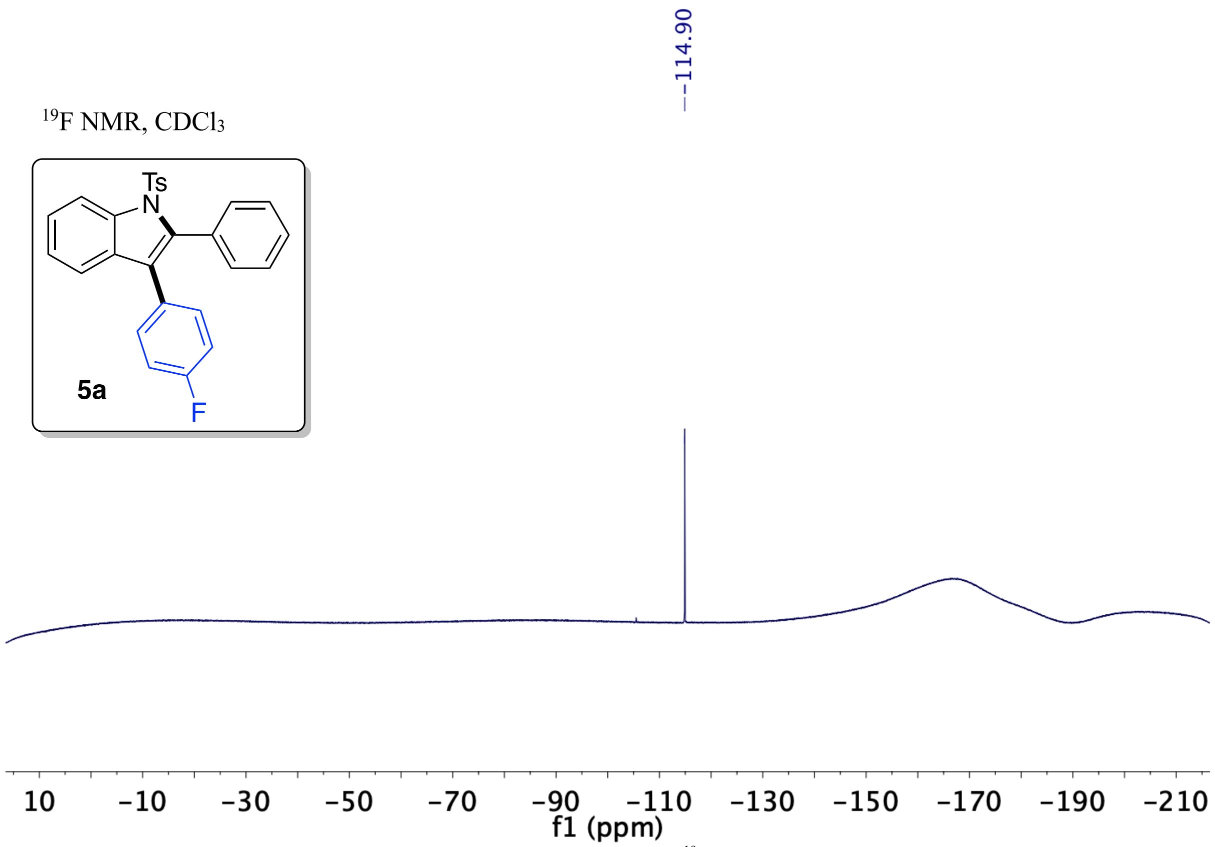

Supplementary Fig. 45. ^{19}F NMR Spectrum for **5a**

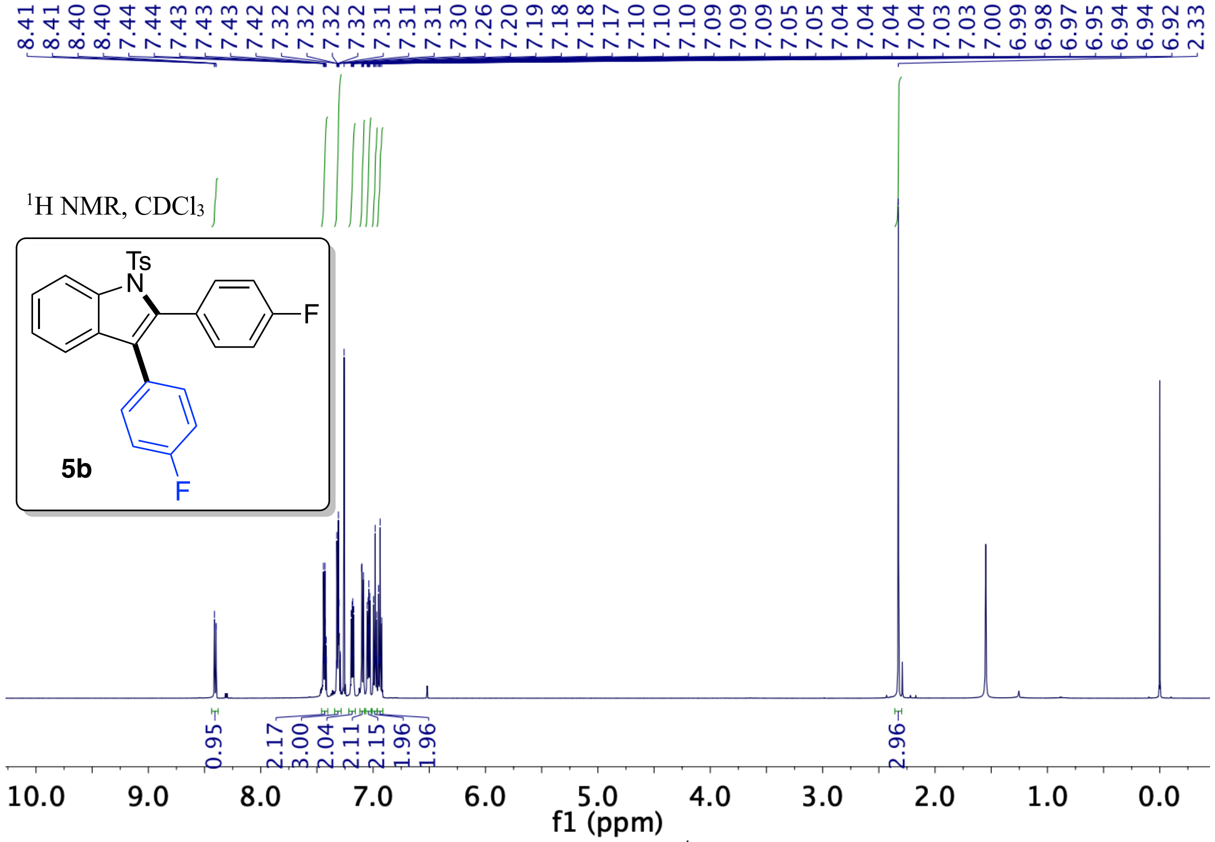

Supplementary Fig. 46. ^{1}H NMR Spectrum for **5b**

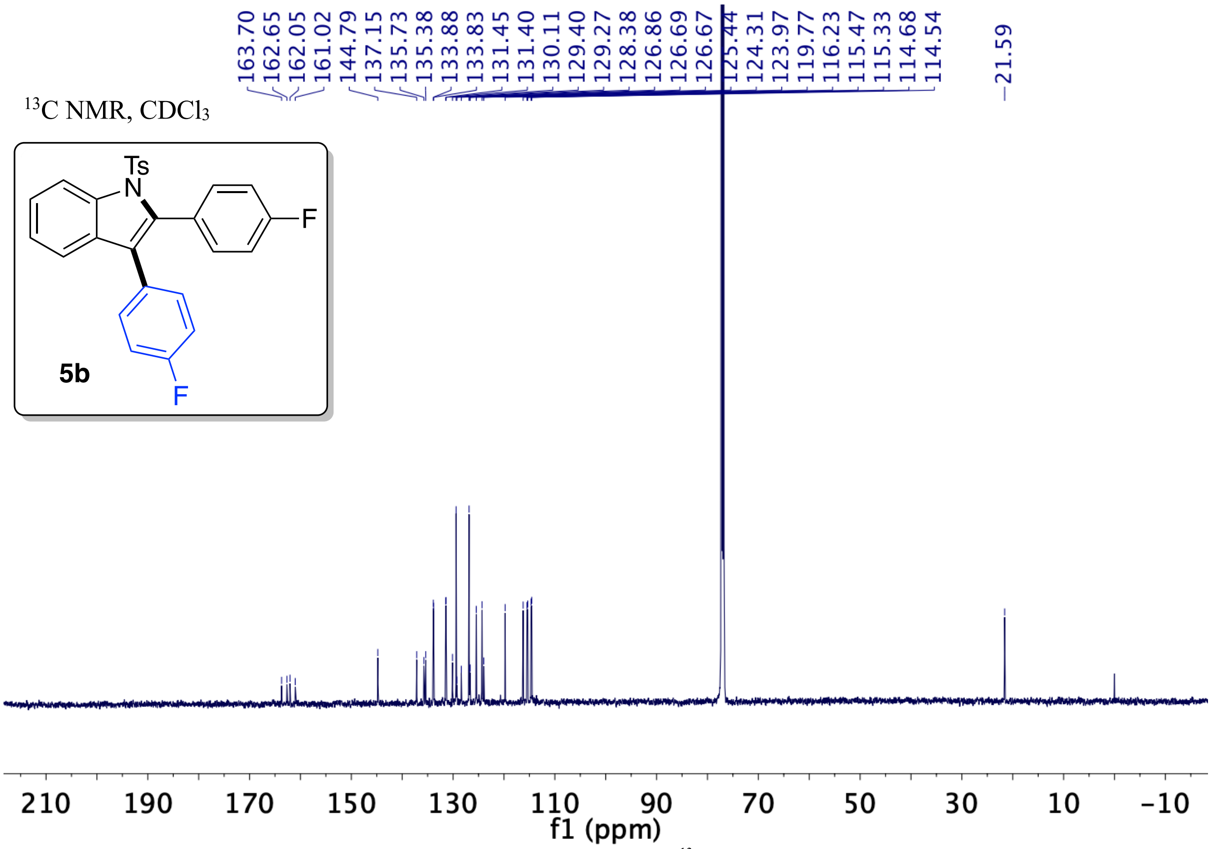

Supplementary Fig. 47. ^{13}C NMR Spectrum for **5b**

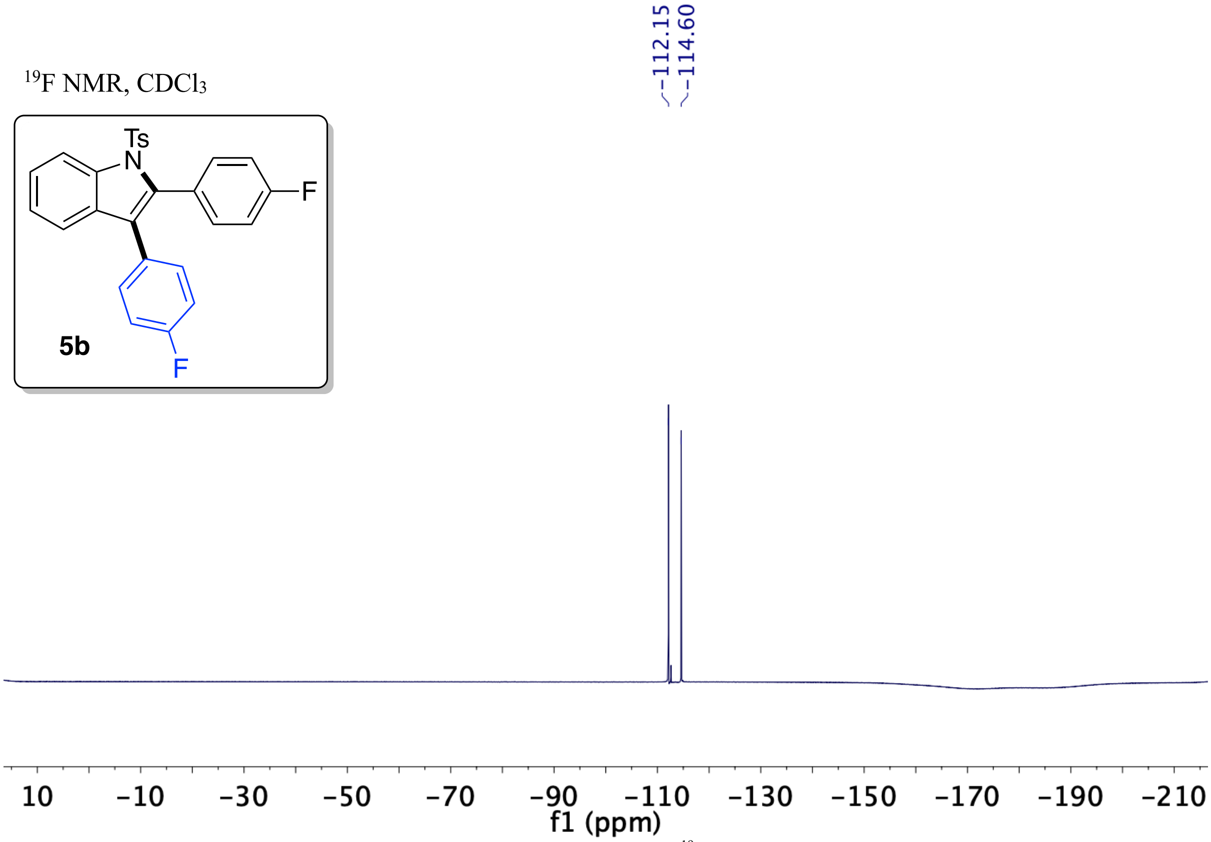

Supplementary Fig. 48. ^{19}F NMR Spectrum for **5b**

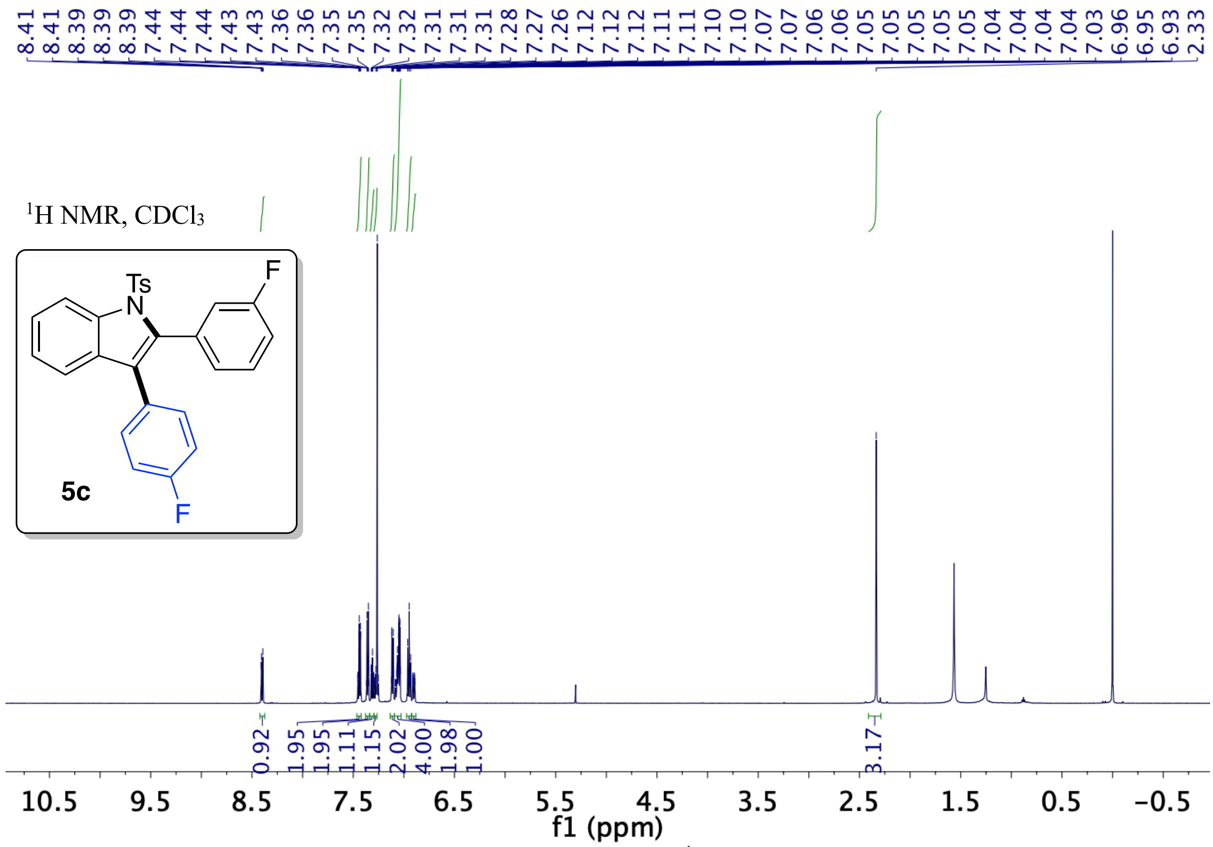

Supplementary Fig. 49. ^{1}H NMR Spectrum for **5c**

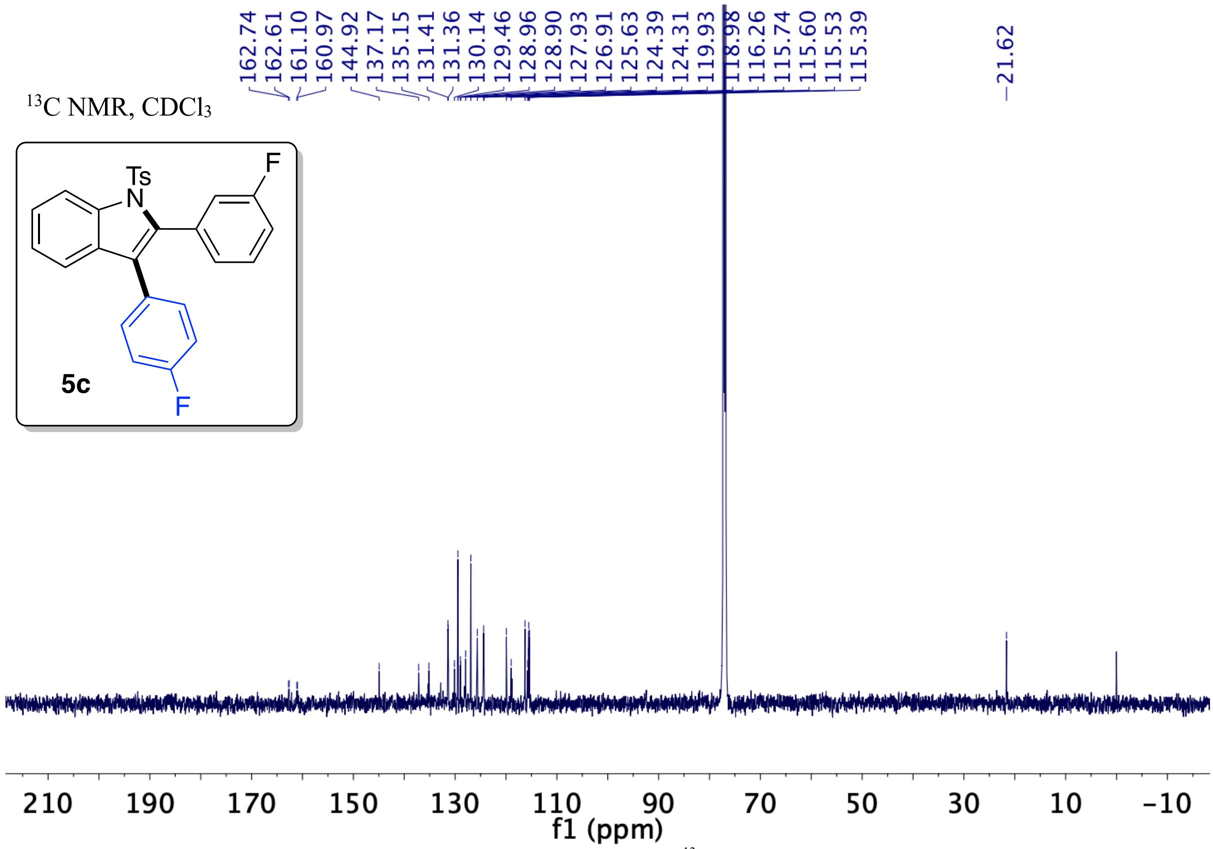

Supplementary Fig. 50. ^{13}C NMR Spectrum for **5c**

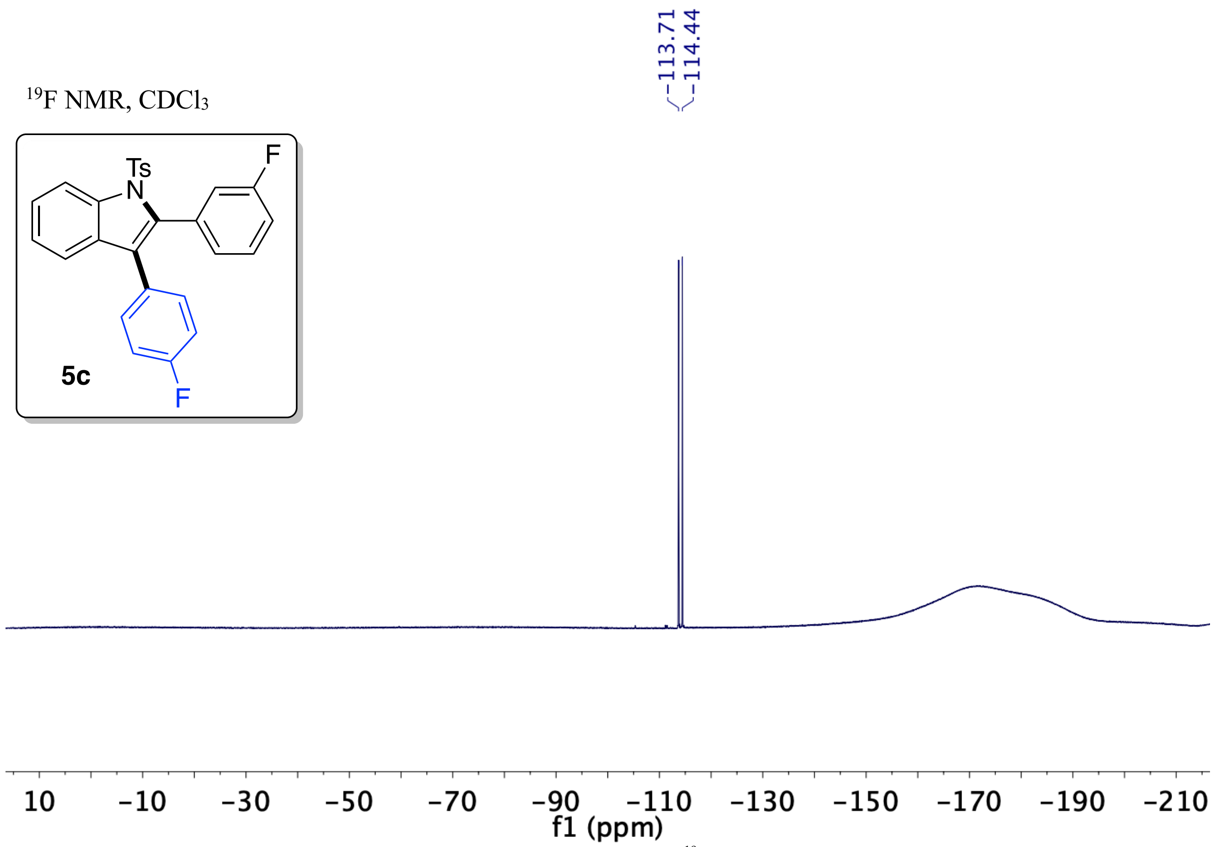

Supplementary Fig. 51. ^{19}F NMR Spectrum for **5c**

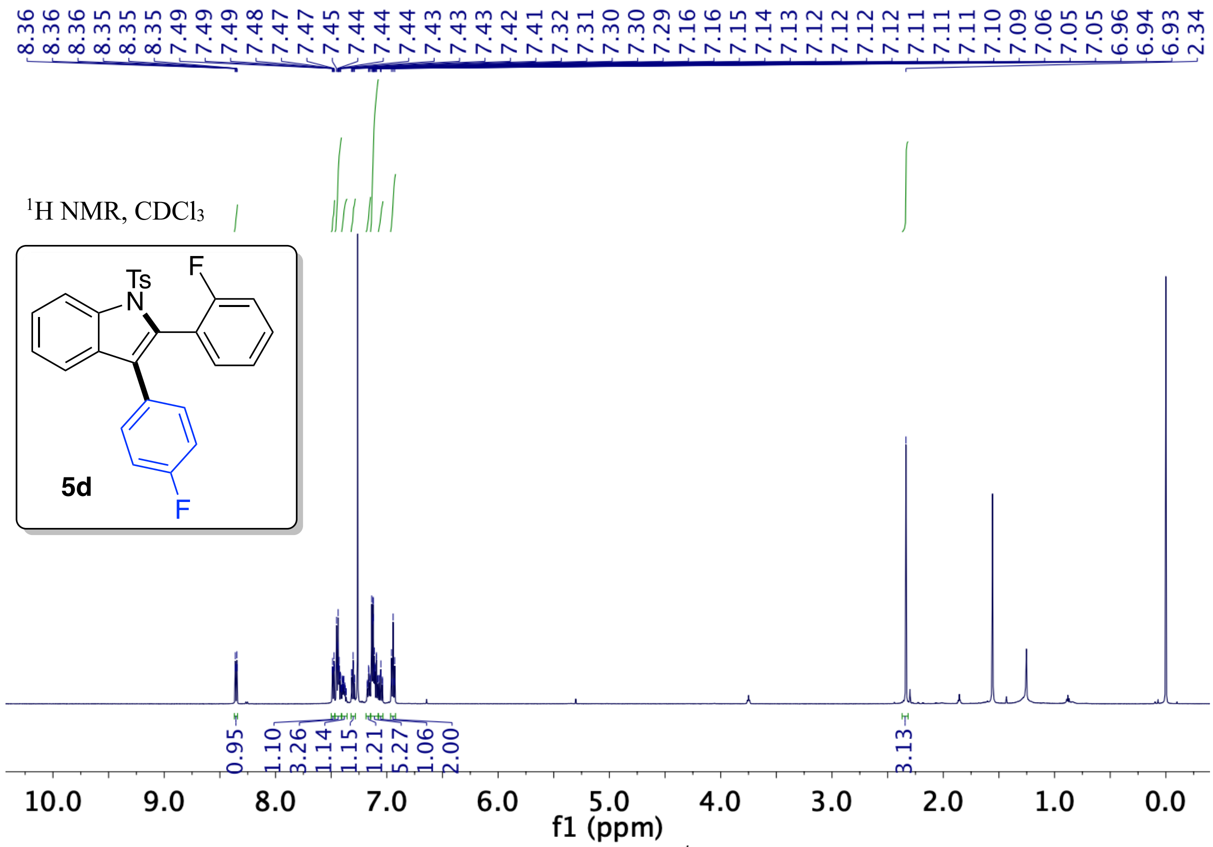

Supplementary Fig. 52. ^{1}H NMR Spectrum for **5d**

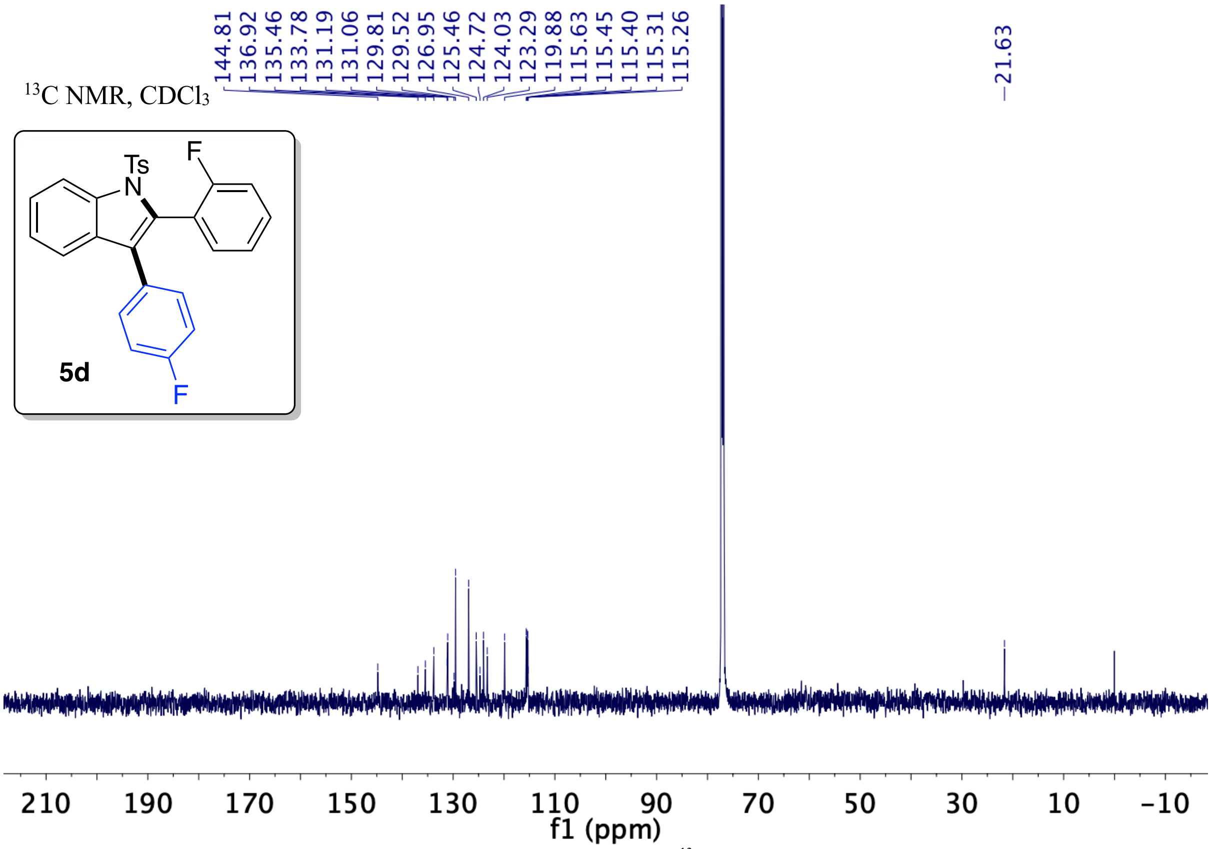

Supplementary Fig. 53. ^{13}C NMR Spectrum for **5d**

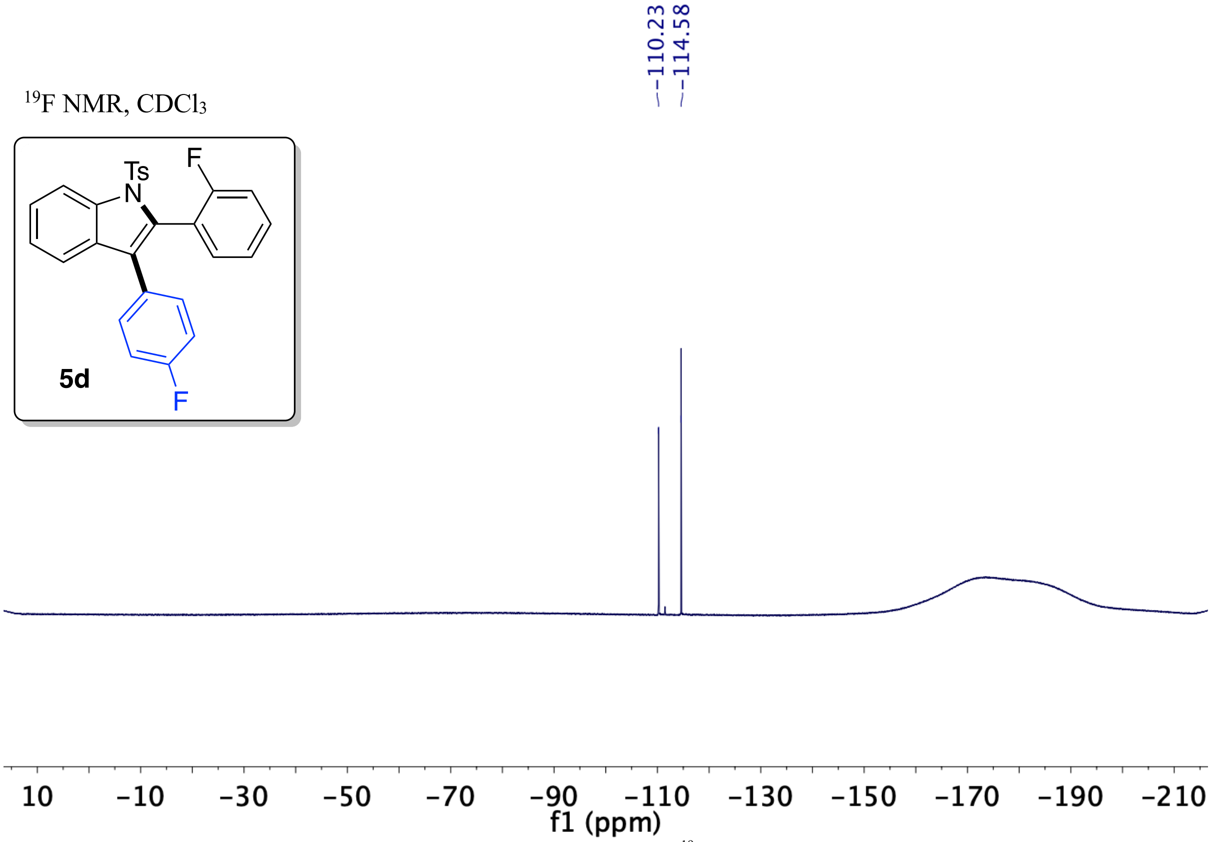

Supplementary Fig. 54. ^{19}F NMR Spectrum for **5d**

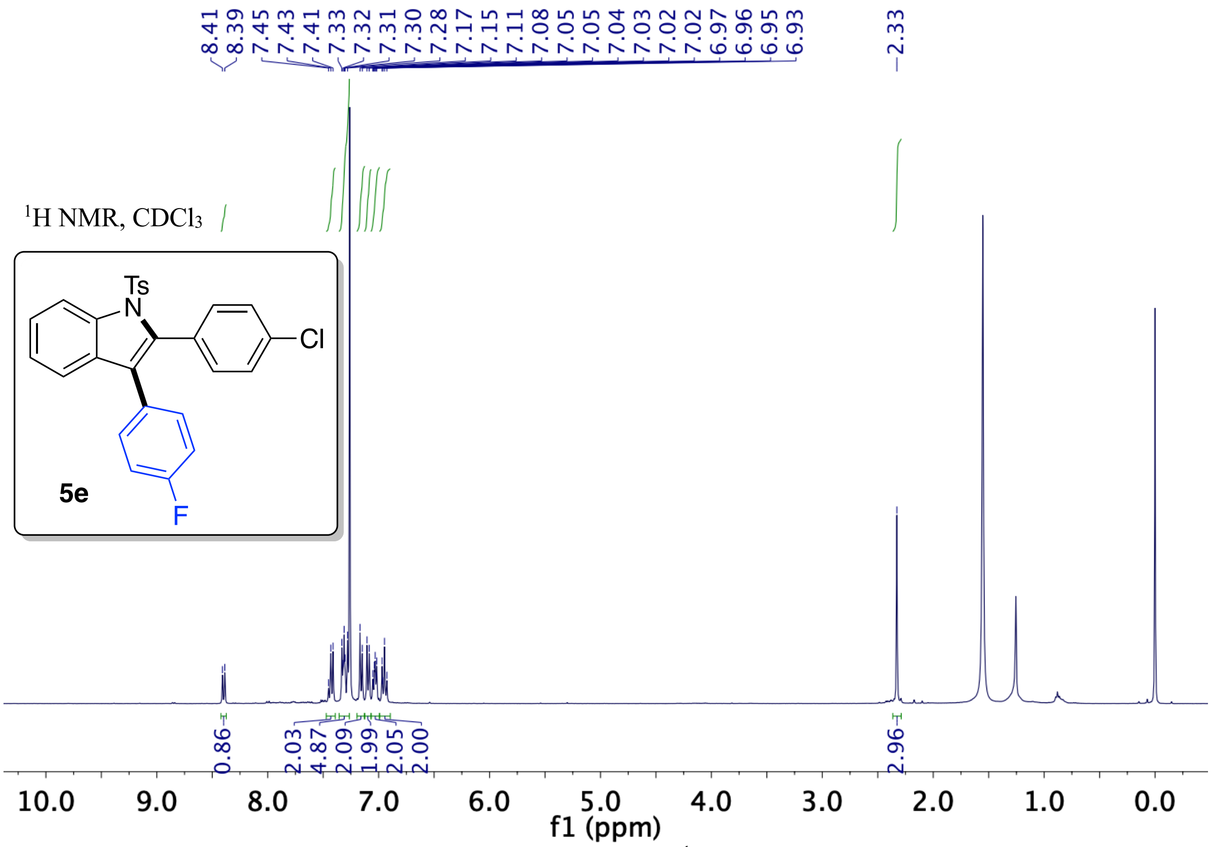

Supplementary Fig. 55. ^{1}H NMR Spectrum for **5e**

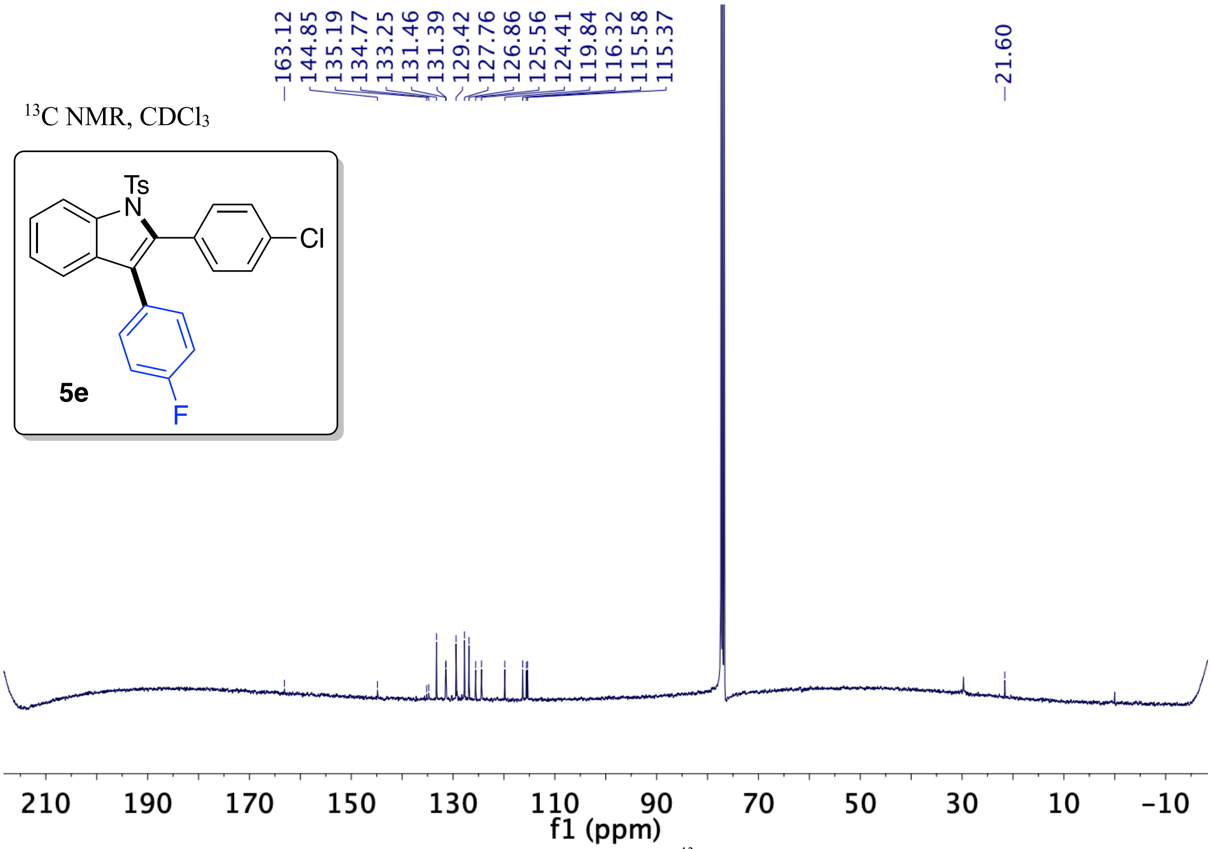

Supplementary Fig. 56. ^{13}C NMR Spectrum for **5e**

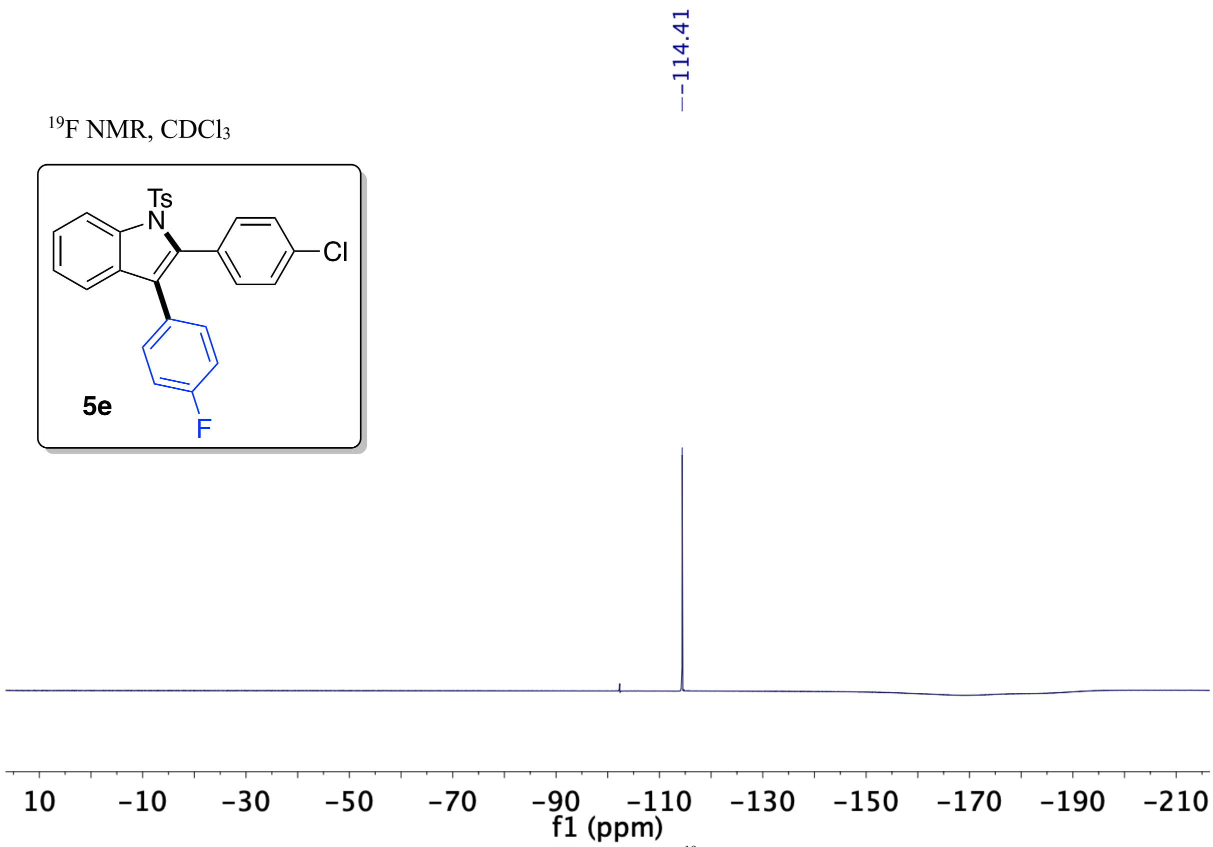

Supplementary Fig. 57. ^{19}F NMR Spectrum for **5e**

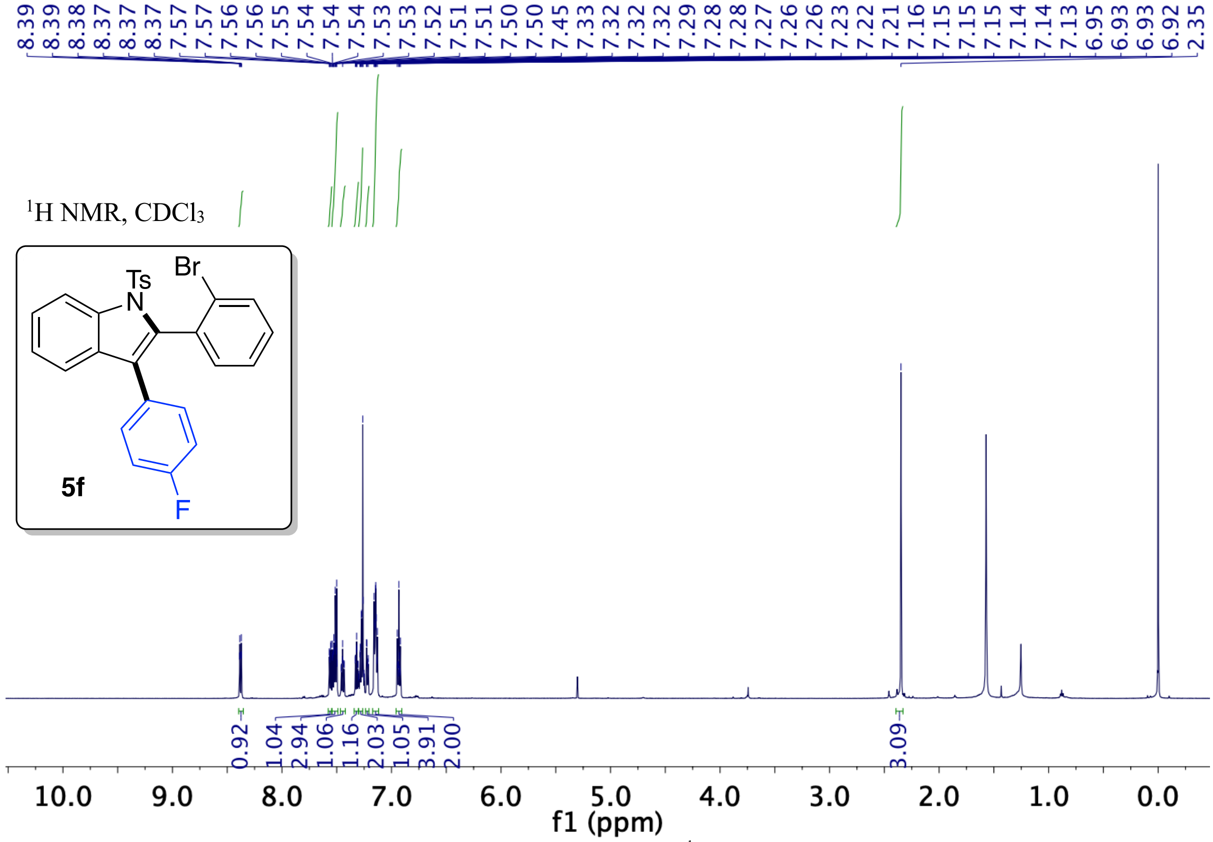

Supplementary Fig. 58. ^{1}H NMR Spectrum for **5f**

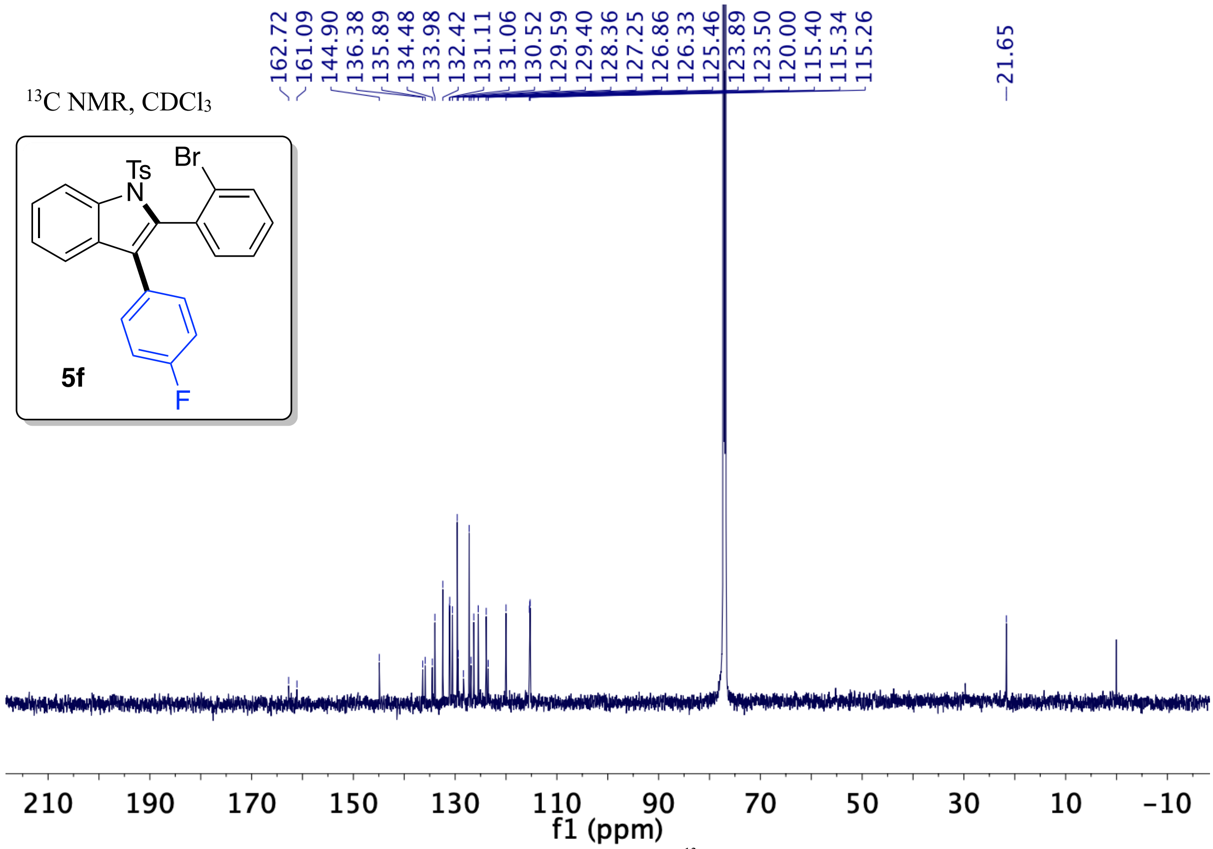

Supplementary Fig. 59. ^{13}C NMR Spectrum for **5f**

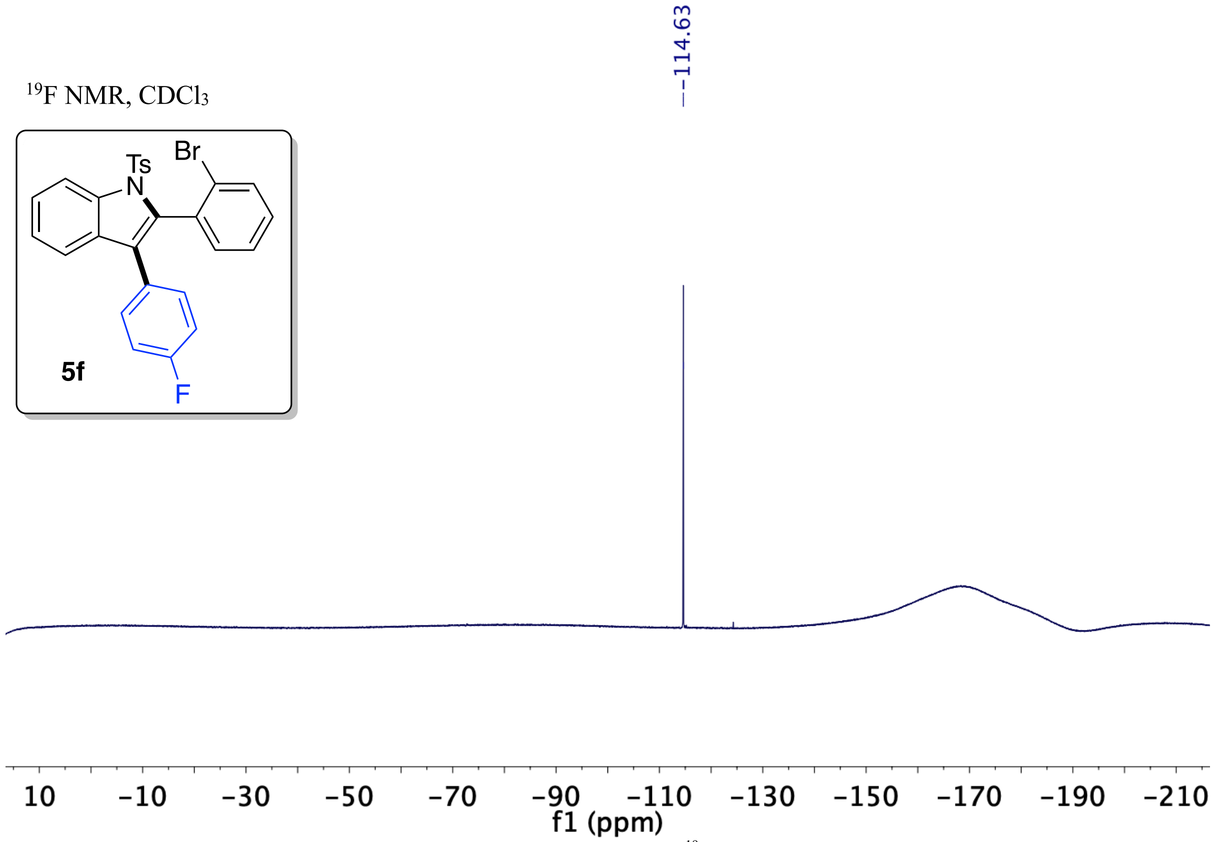

Supplementary Fig. 60. ^{19}F NMR Spectrum for **5f**

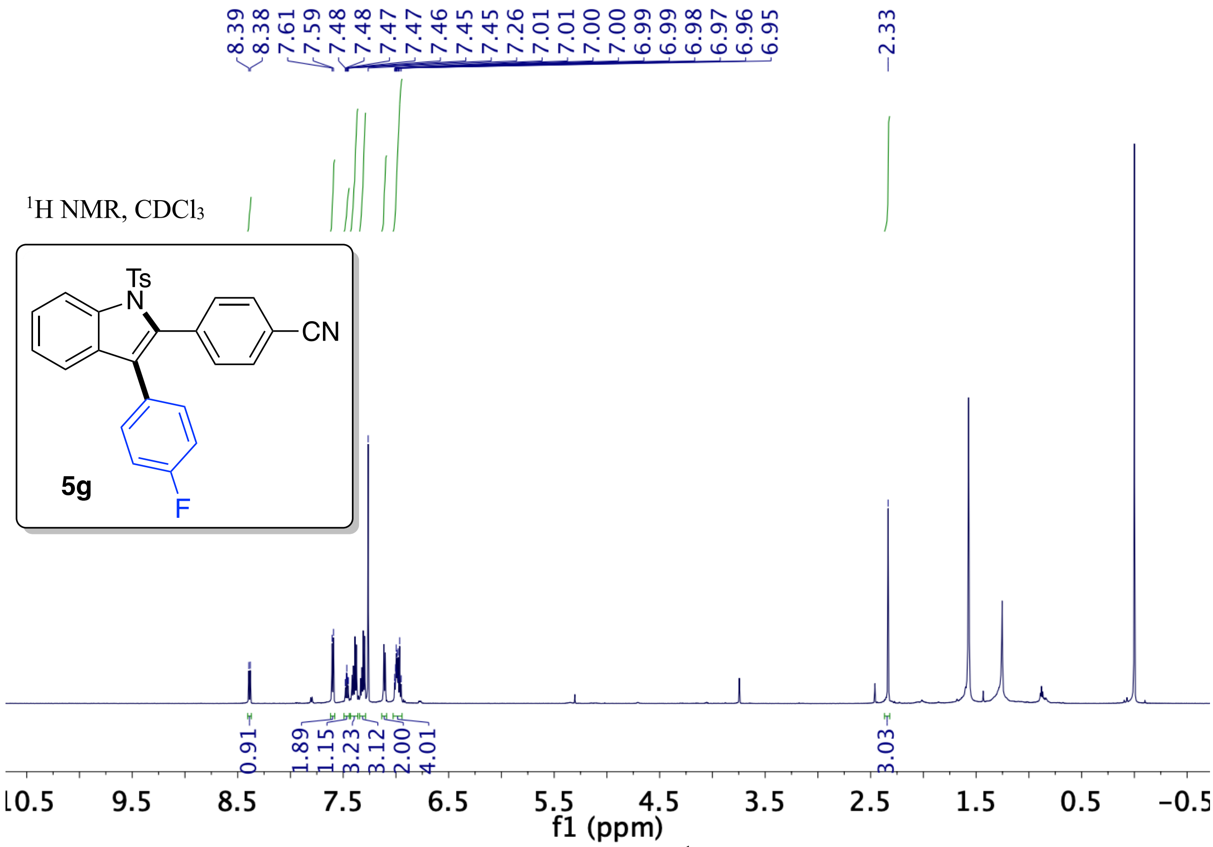

Supplementary Fig. 61. ^{1}H NMR Spectrum for **5g**

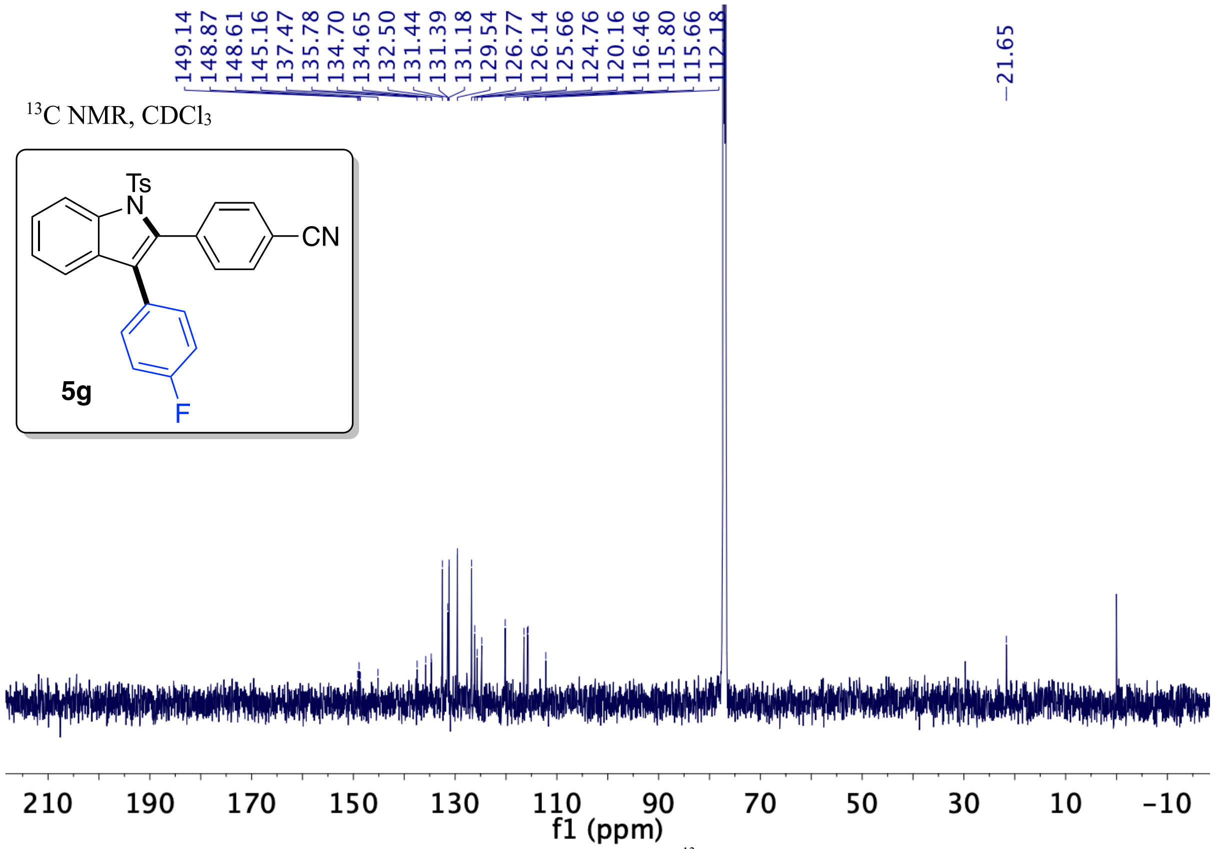

Supplementary Fig. 62. ^{13}C NMR Spectrum for **5g**

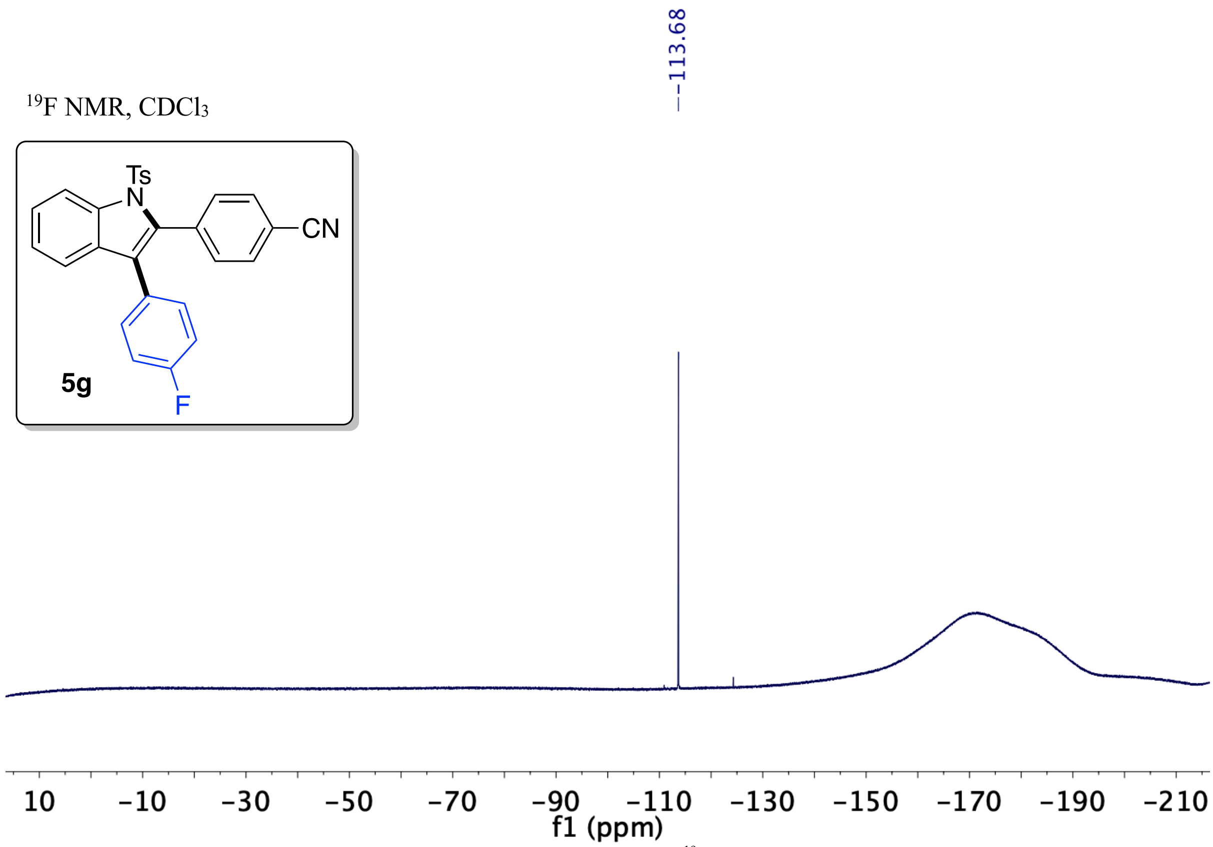

Supplementary Fig. 63. ^{19}F NMR Spectrum for **5g**

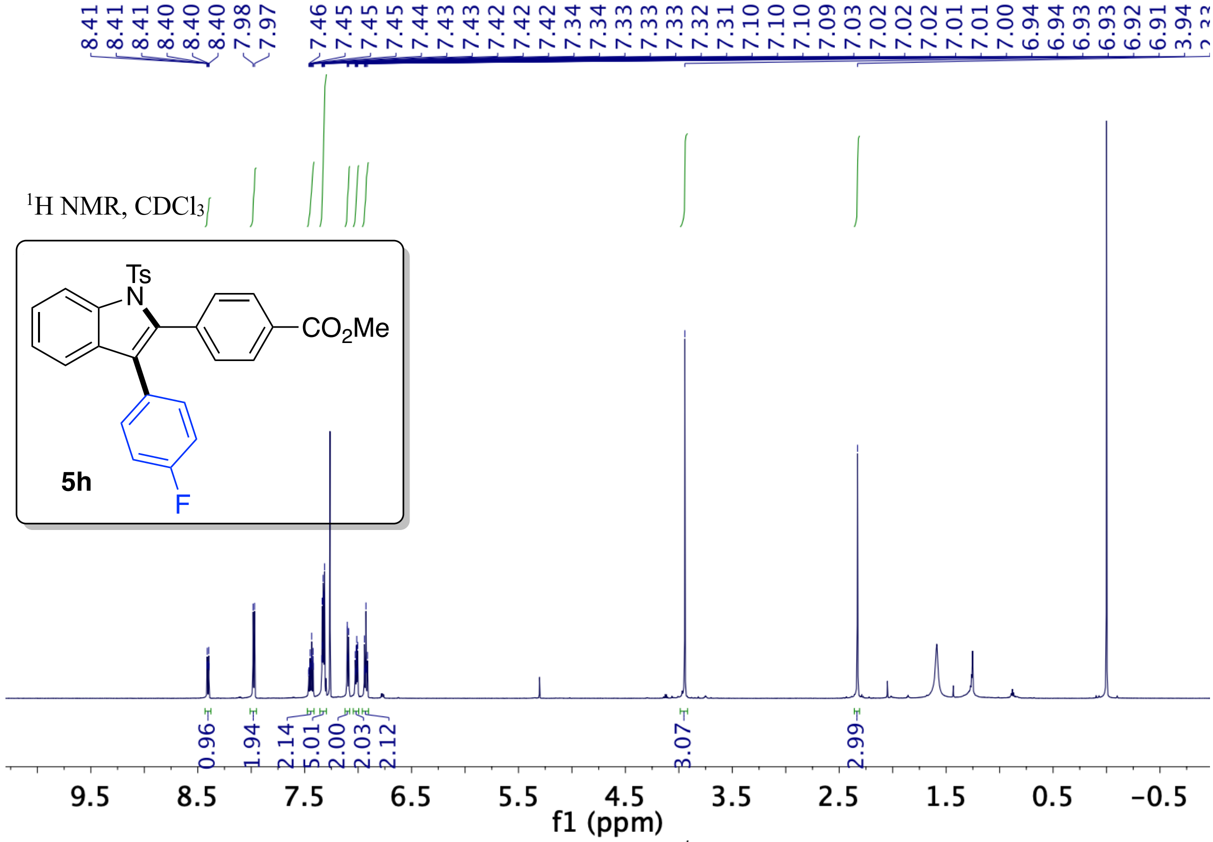

Supplementary Fig. 64. ^{1}H NMR Spectrum for **5h**

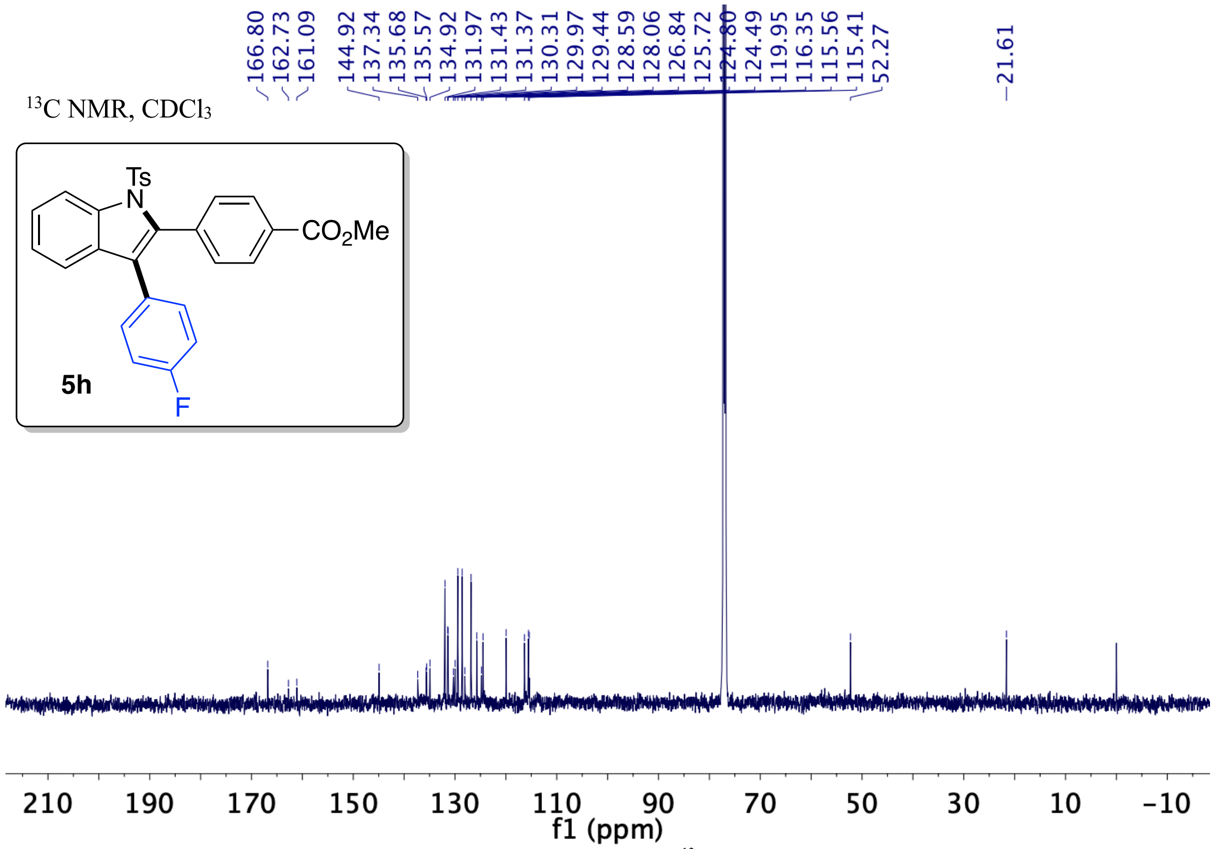

Supplementary Fig. 65. ^{13}C NMR Spectrum for **5h**

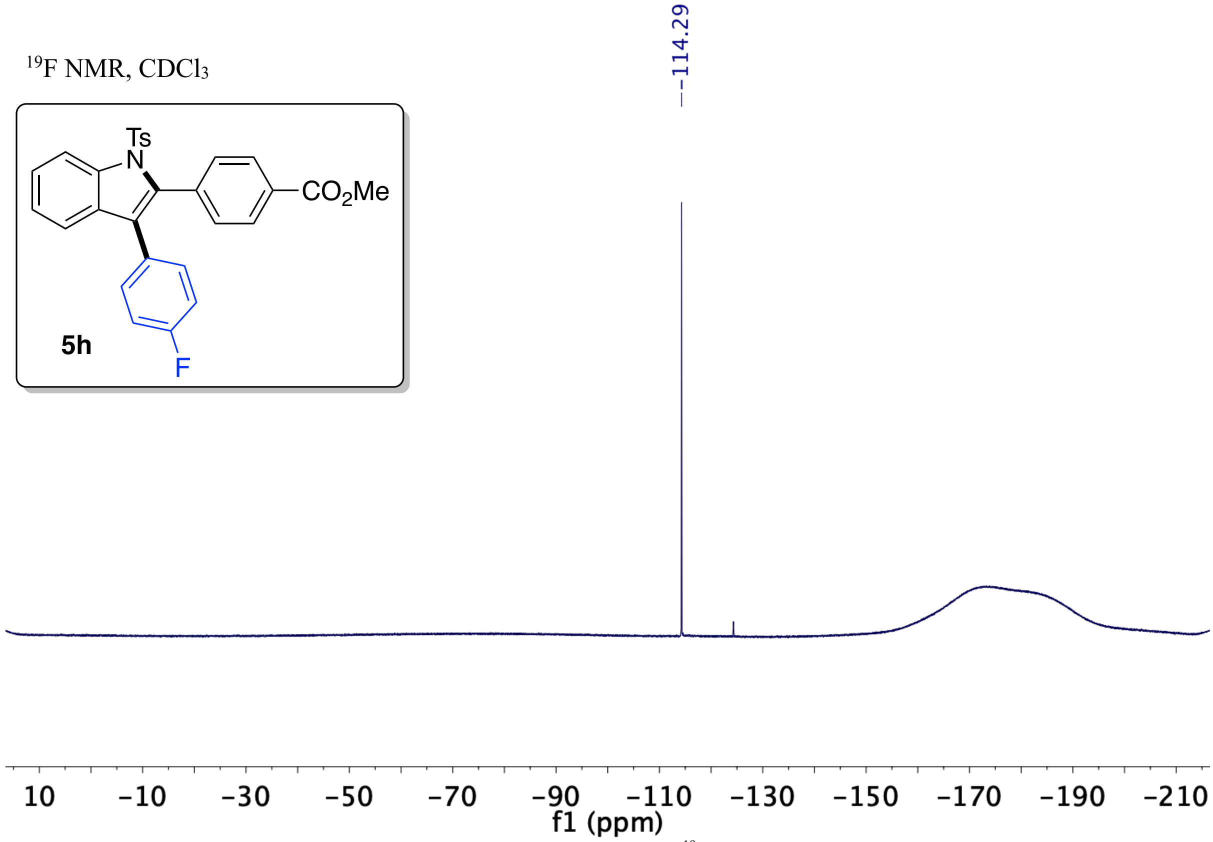

Supplementary Fig. 66. ^{19}F NMR Spectrum for **5h**

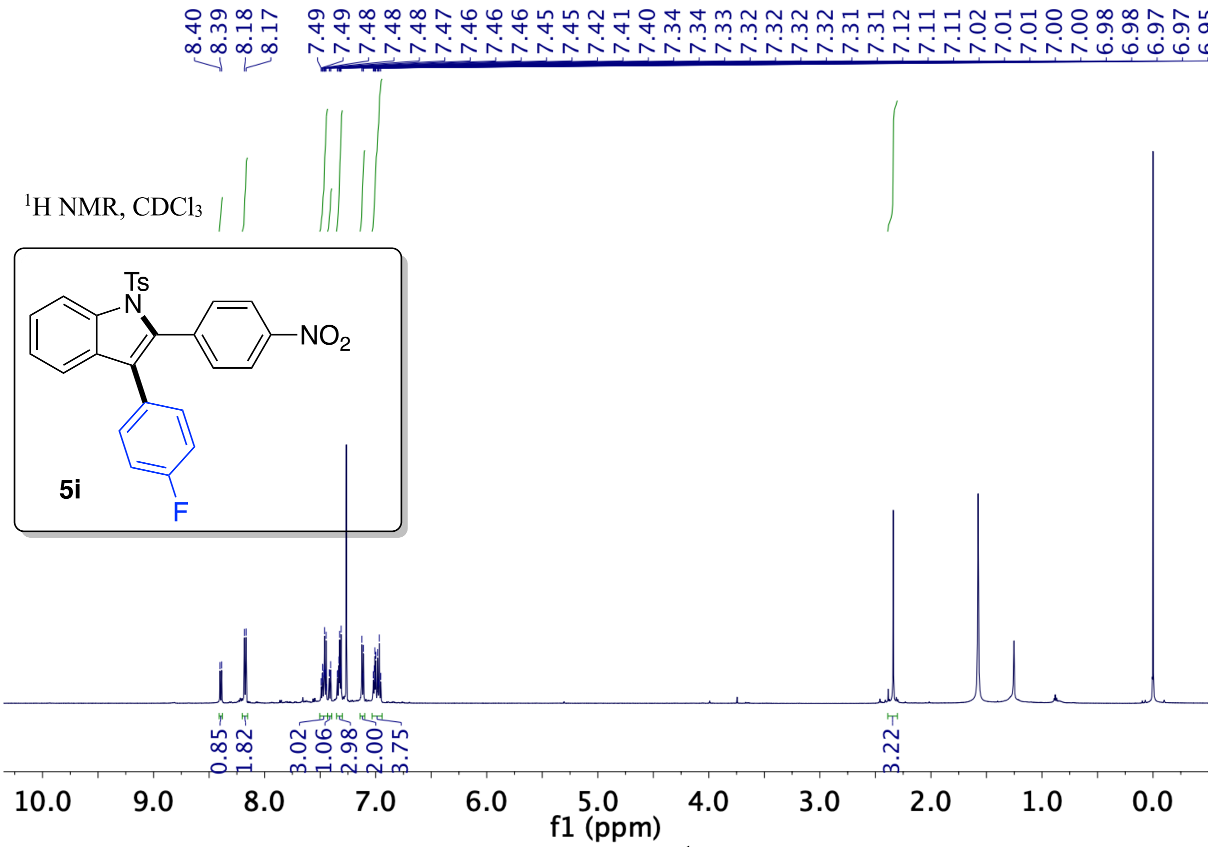

Supplementary Fig. 67. ^{1}H NMR Spectrum for **5i**

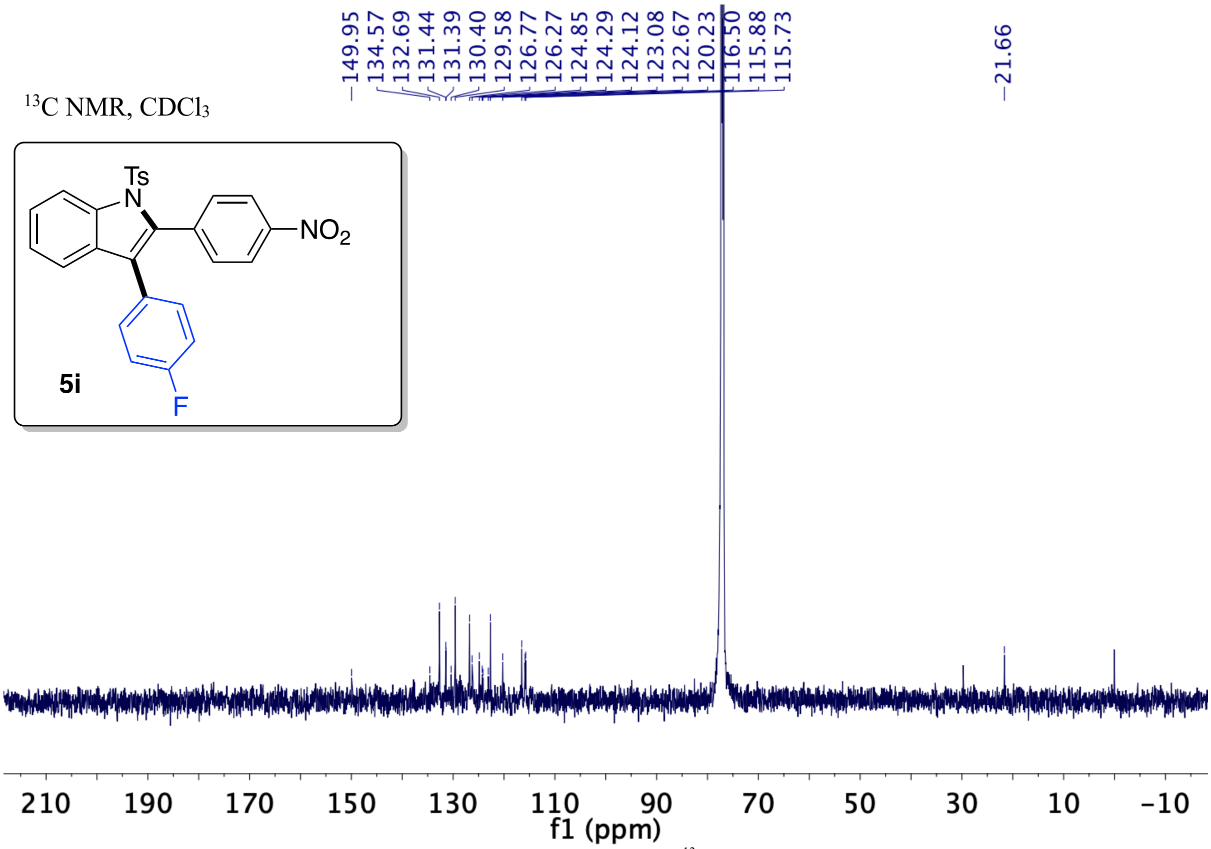

Supplementary Fig. 68. ^{13}C NMR Spectrum for **5i**

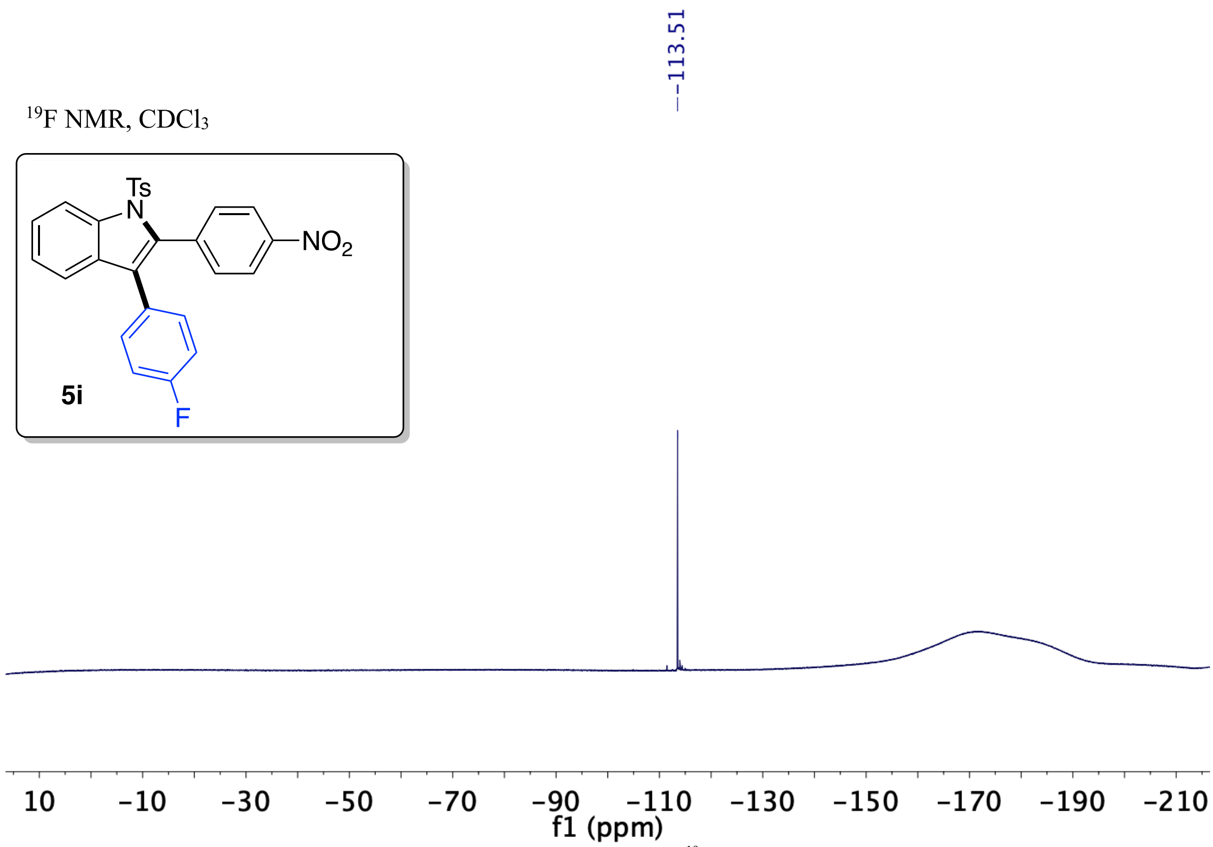

Supplementary Fig. 69. ^{19}F NMR Spectrum for **5i**

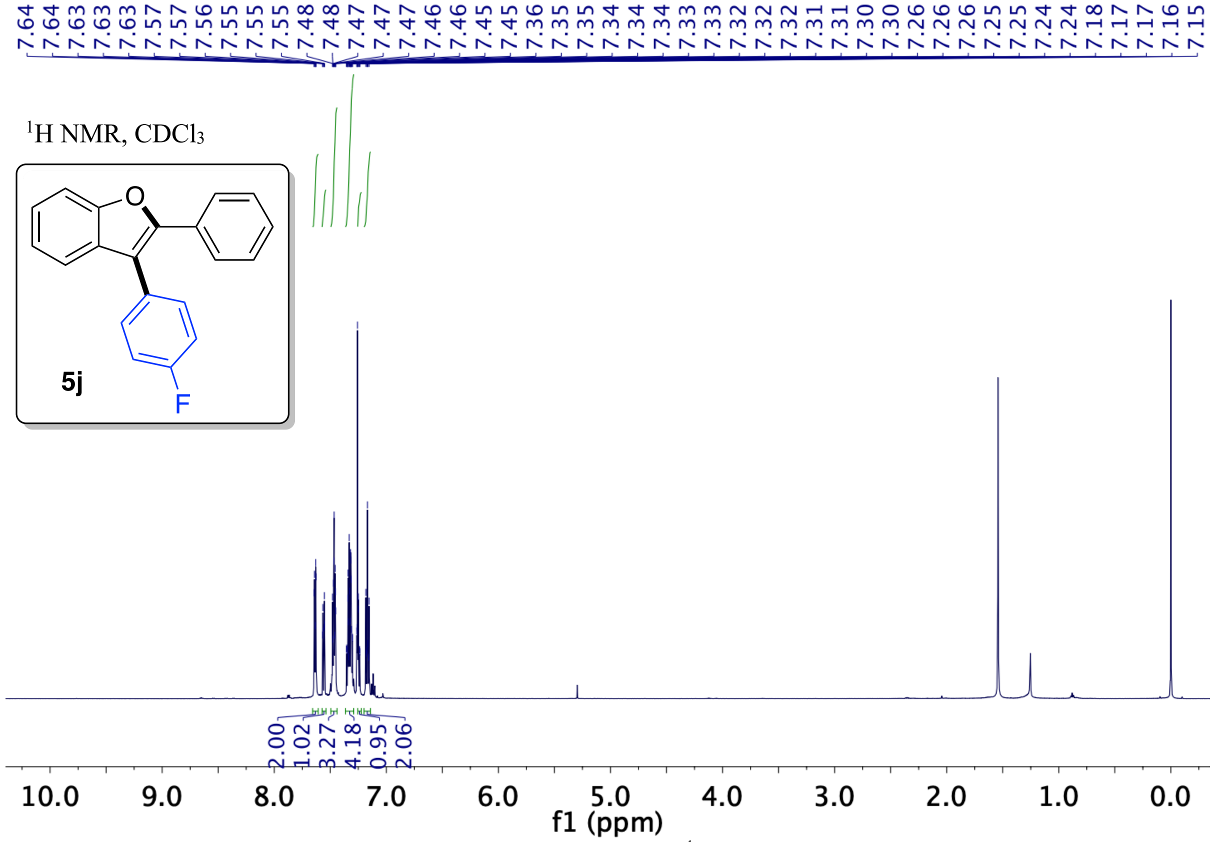

Supplementary Fig. 70. ^{1}H NMR Spectrum for **5j**

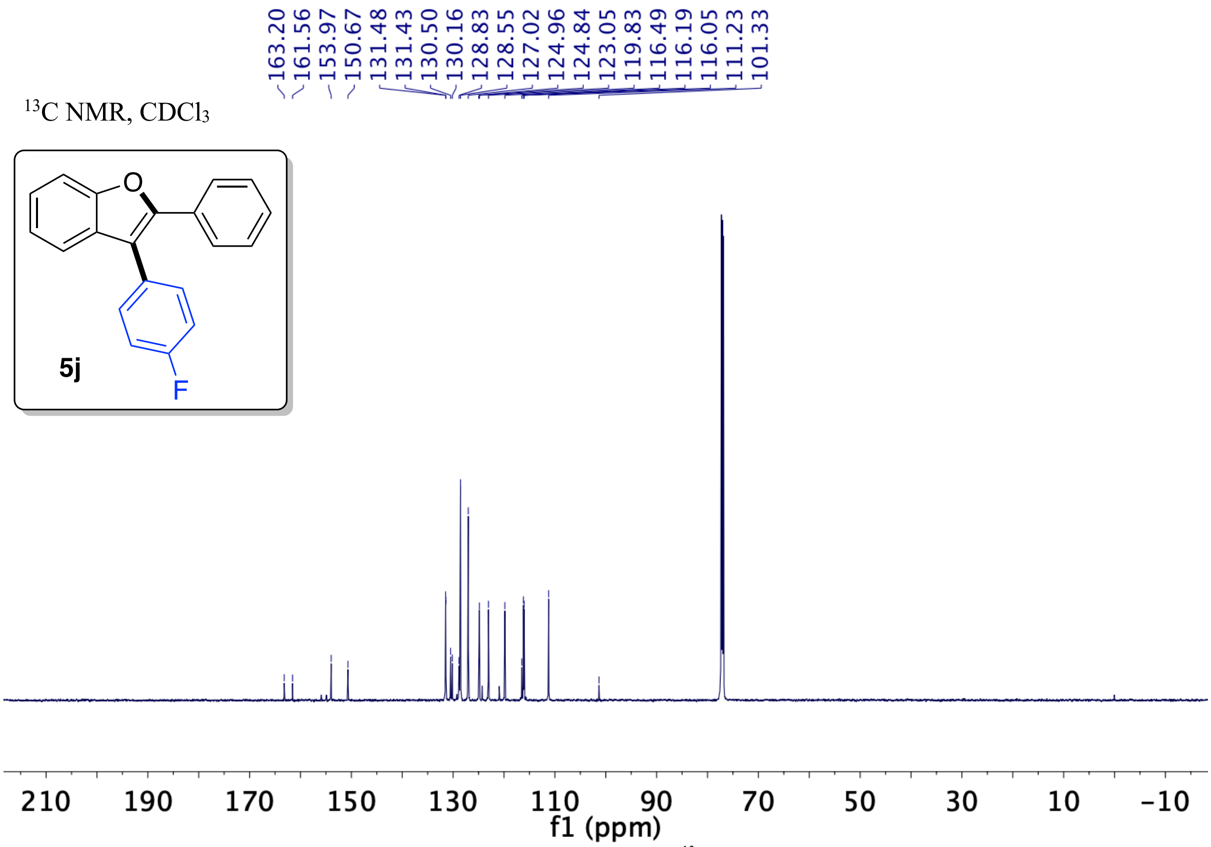

Supplementary Fig. 71. ^{13}C NMR Spectrum for **5j**

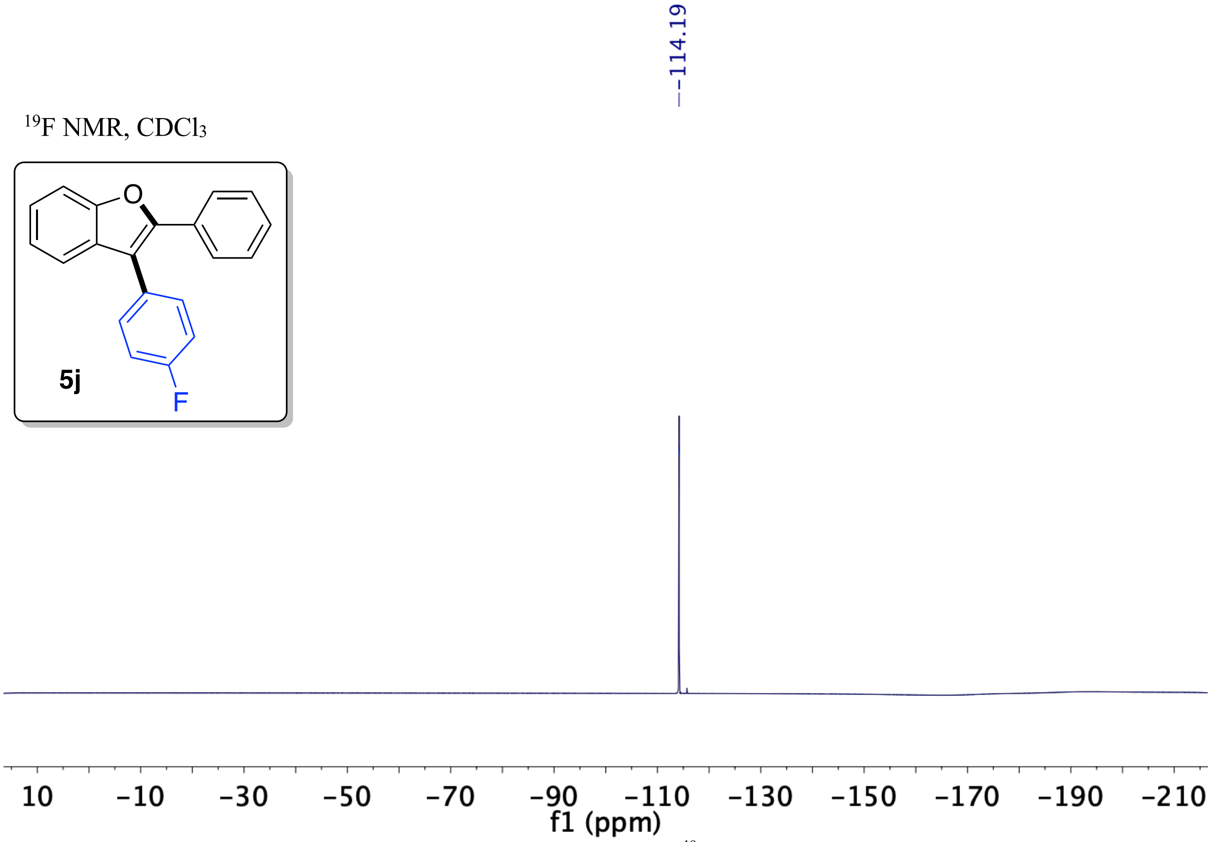

Supplementary Fig. 72. ^{19}F NMR Spectrum for **5j**

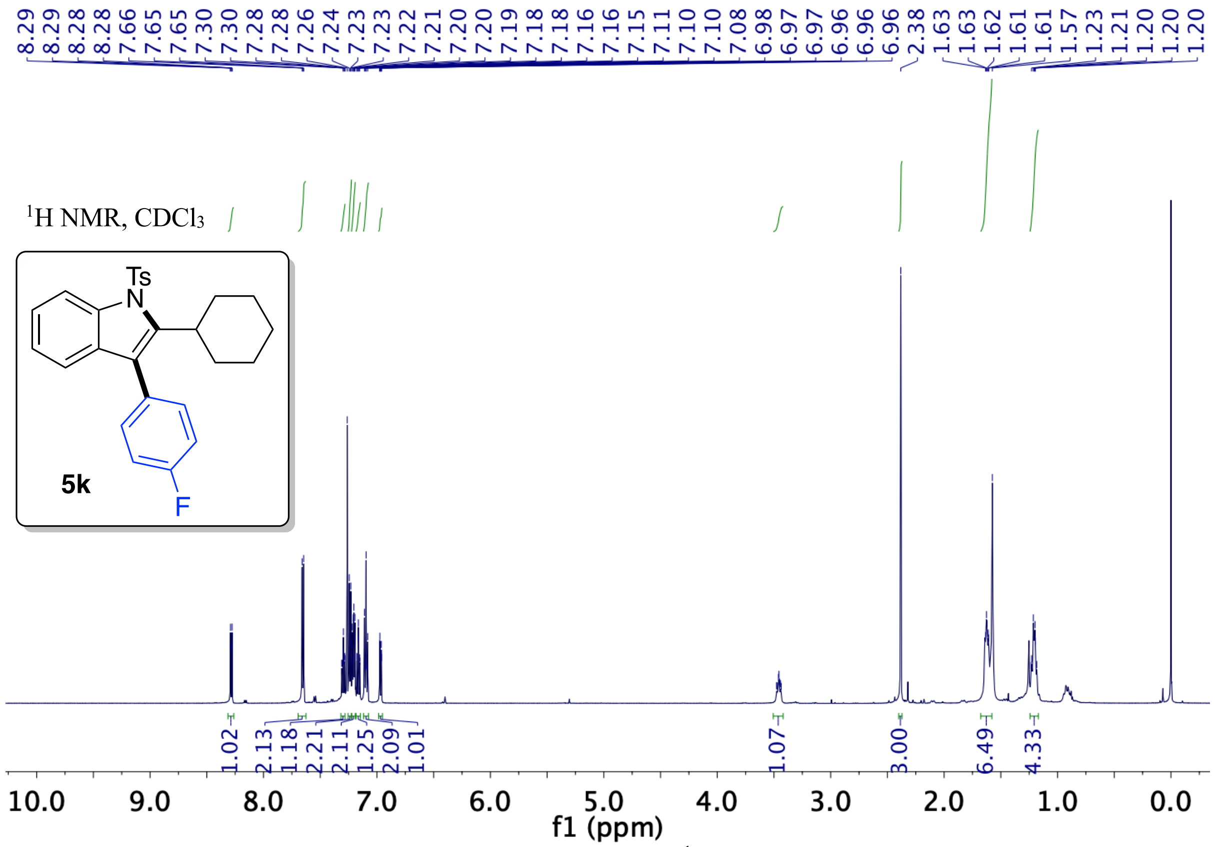

Supplementary Fig. 73. ^{1}H NMR Spectrum for **5k**

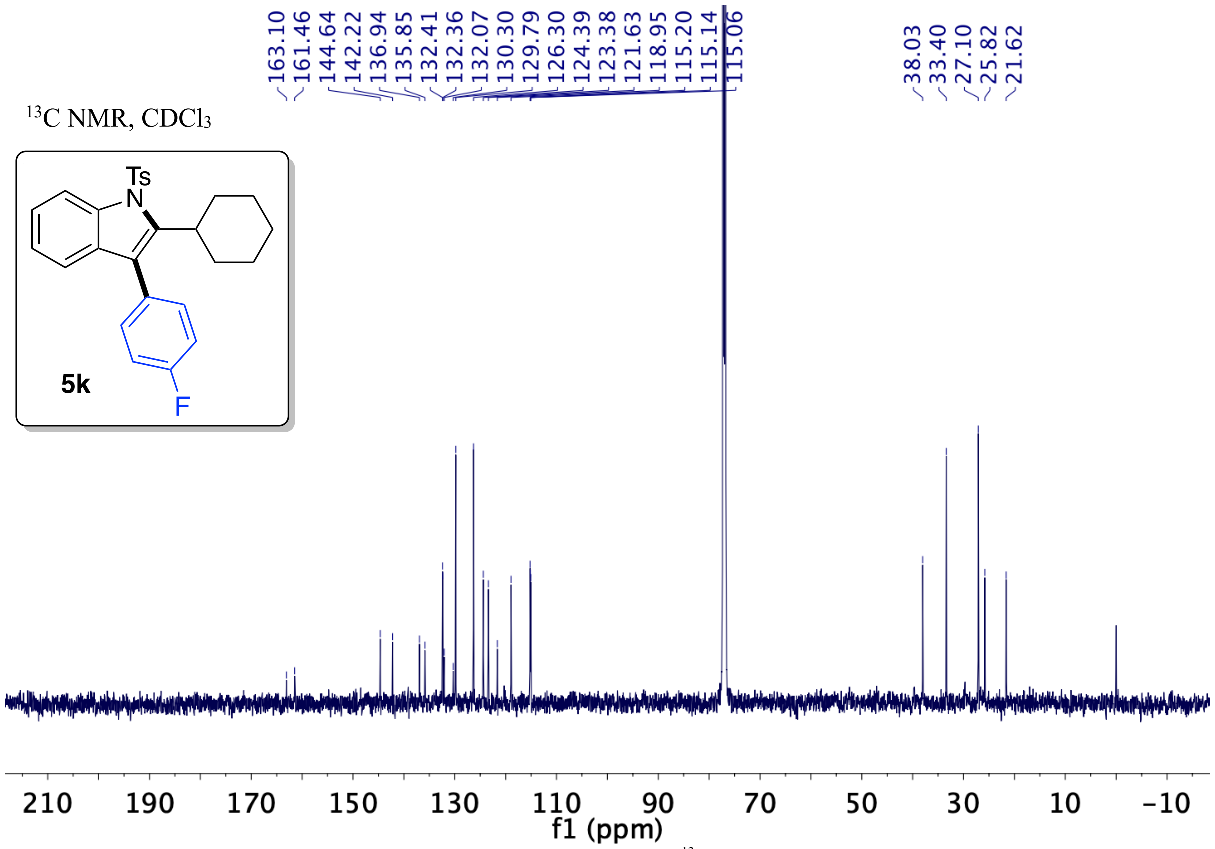

Supplementary Fig. 74. ^{13}C NMR Spectrum for **5k**

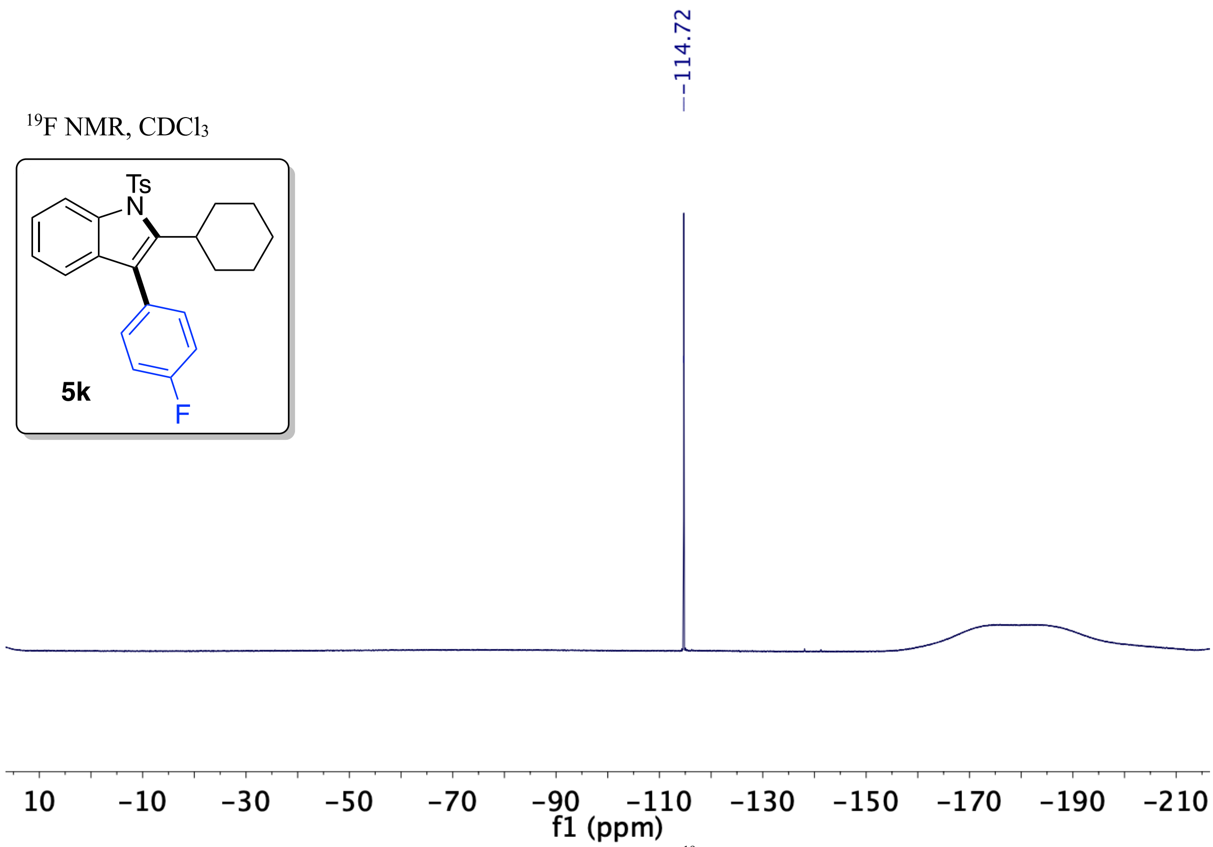

Supplementary Fig. 75. ^{19}F NMR Spectrum for **5k**

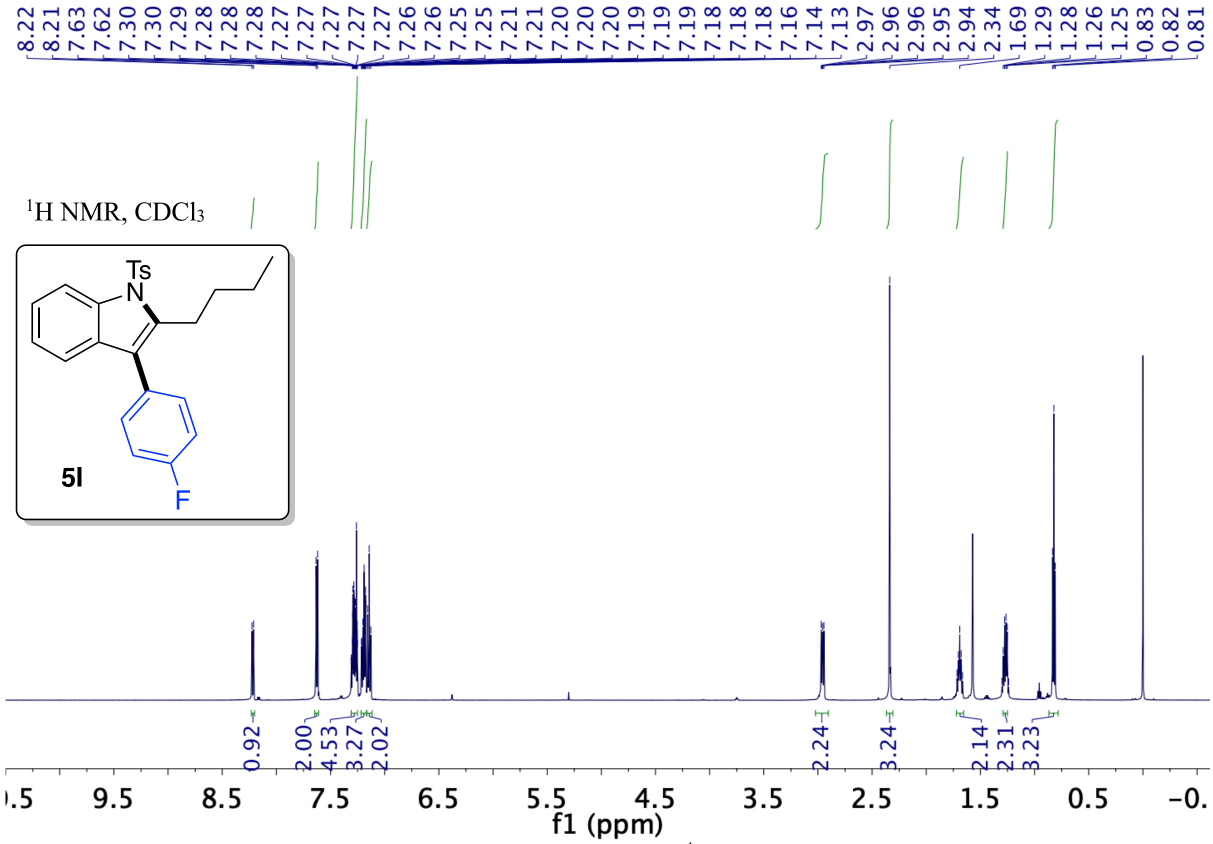

Supplementary Fig. 76. ^{1}H NMR Spectrum for **5l**

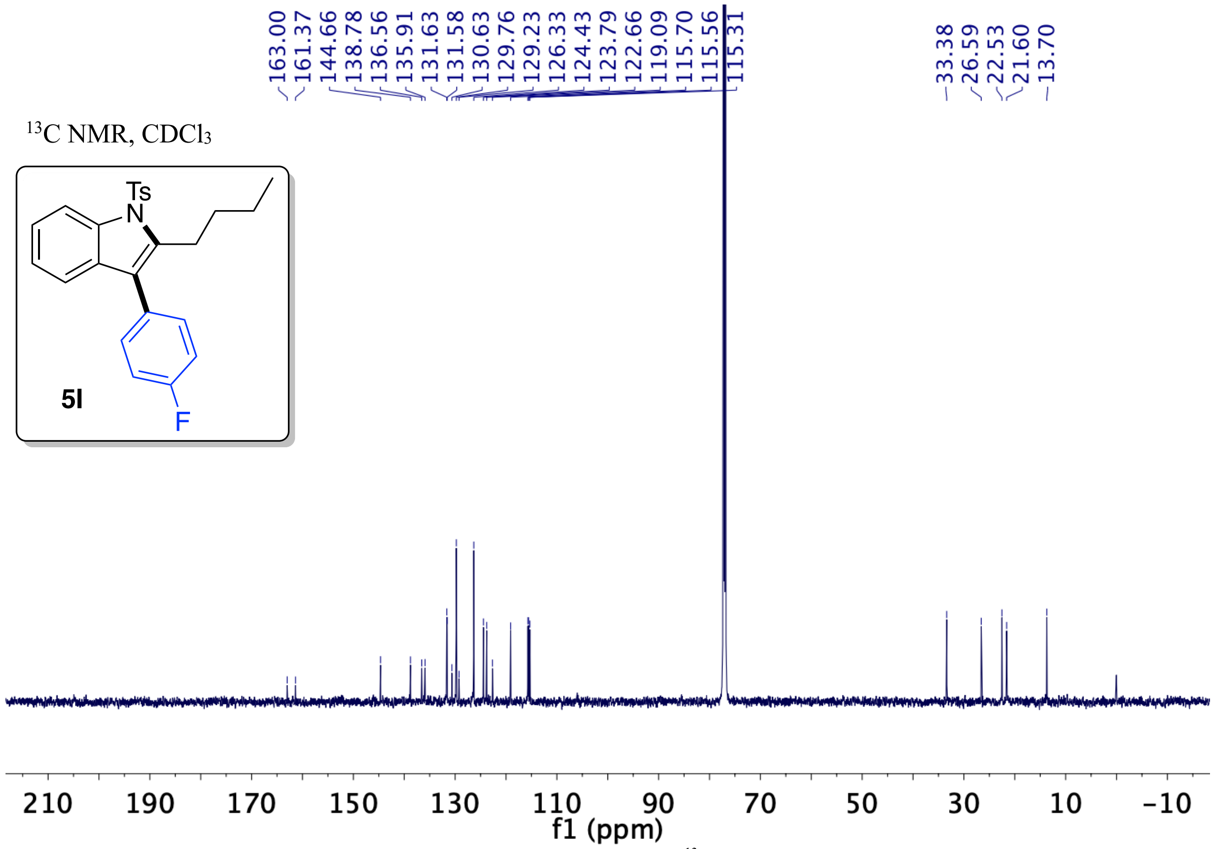

Supplementary Fig. 77. ^{13}C NMR Spectrum for **5l**

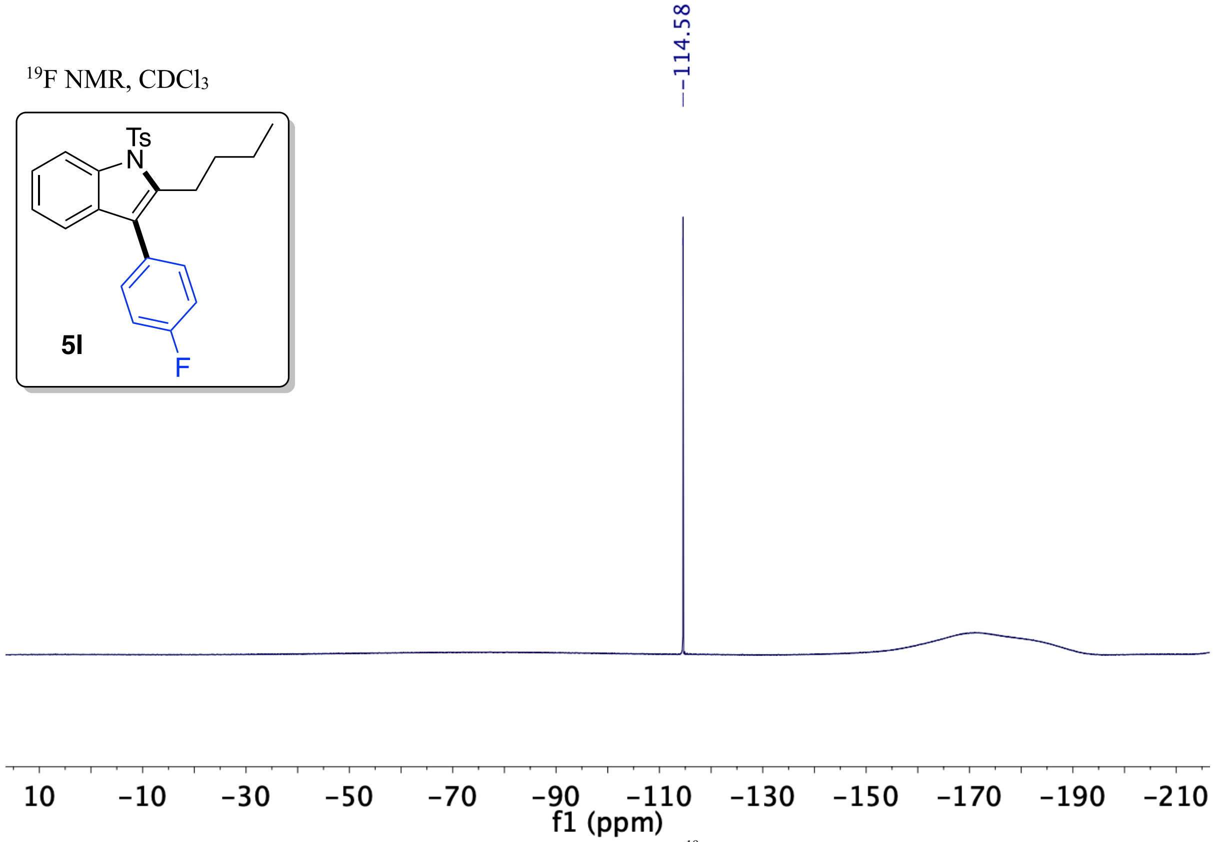

Supplementary Fig. 78. ^{19}F NMR Spectrum for **5l**

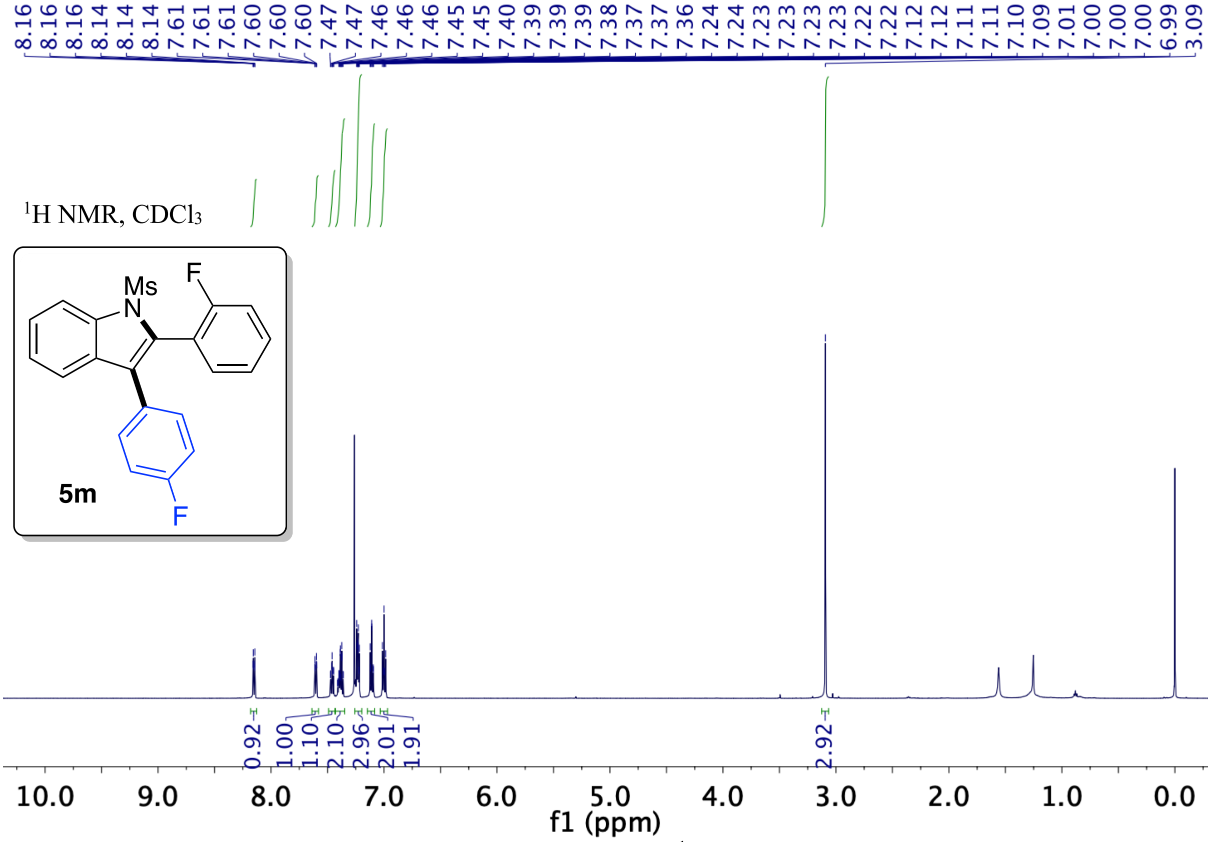

Supplementary Fig. 79. ^{1}H NMR Spectrum for **5m**

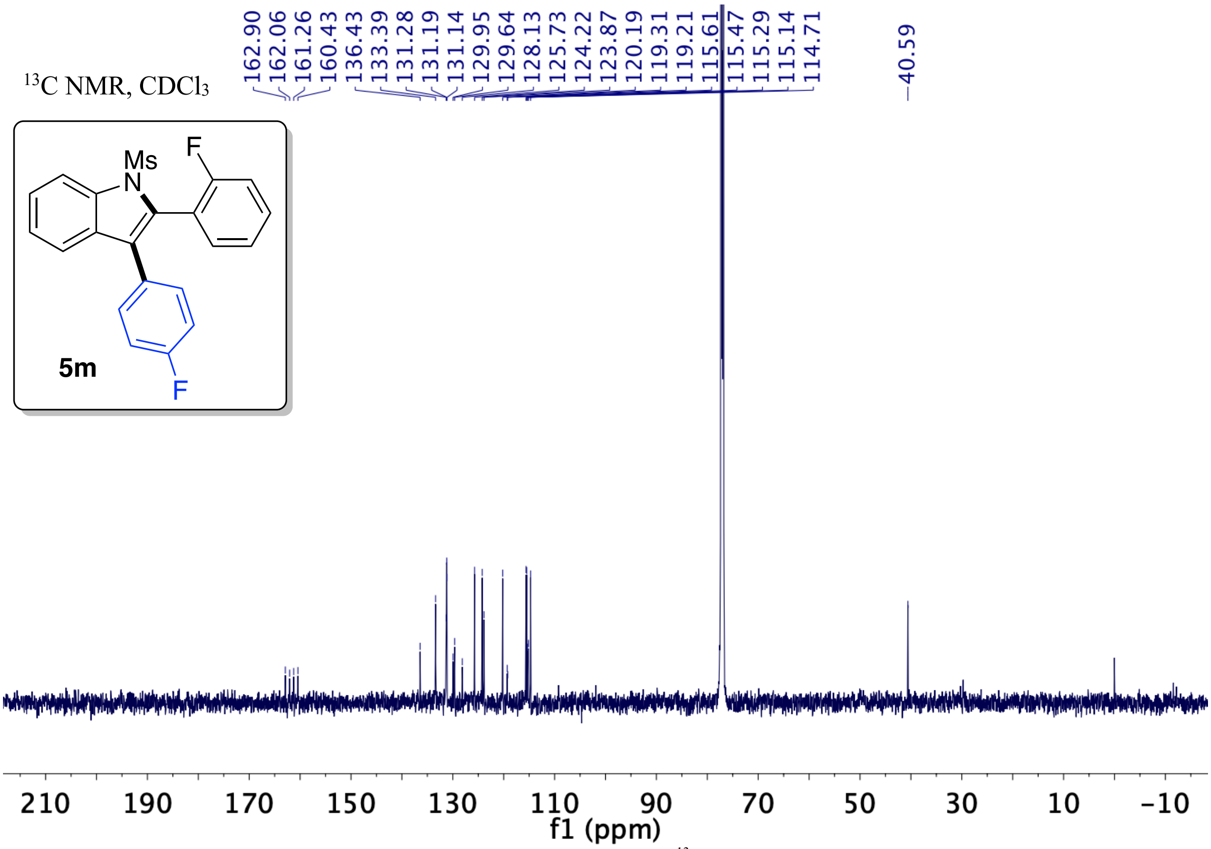

Supplementary Fig. 80. ^{13}C NMR Spectrum for **5m**

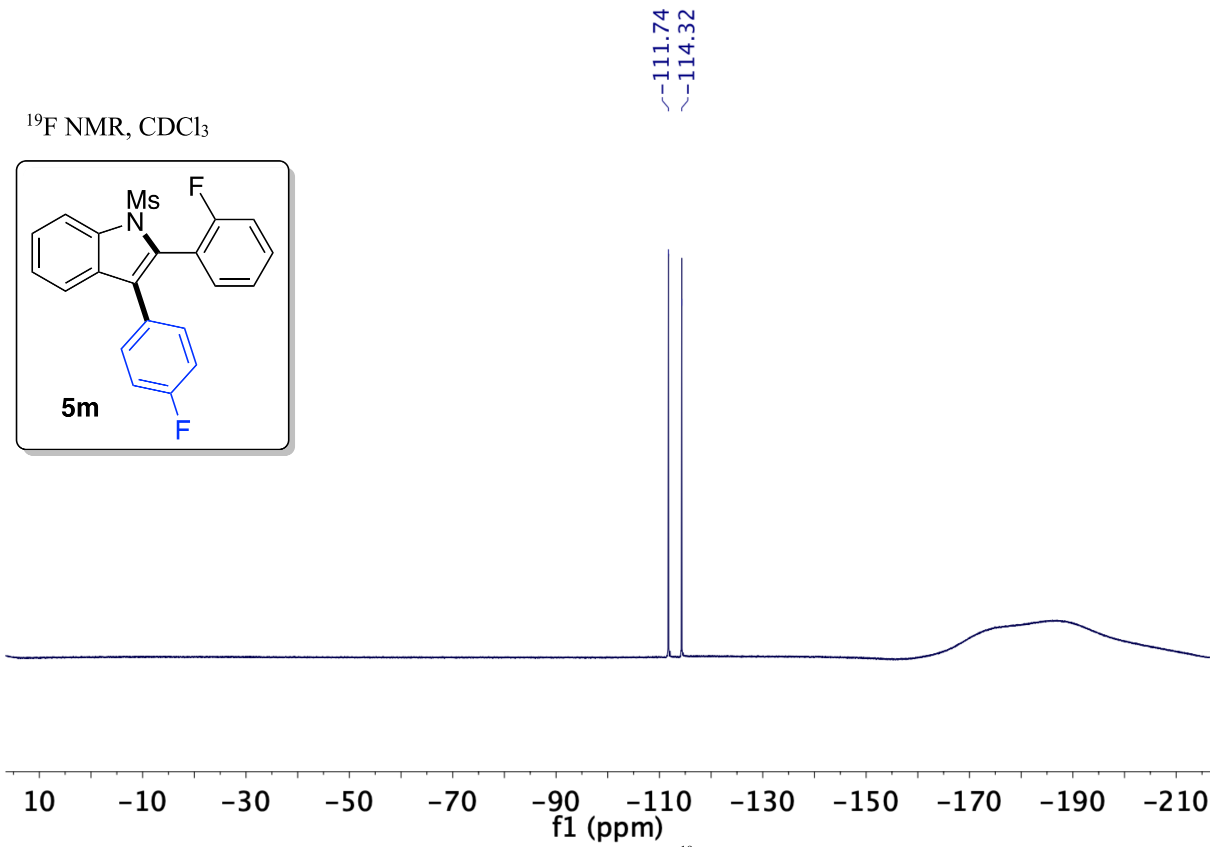

Supplementary Fig. 81. ^{19}F NMR Spectrum for **5m**

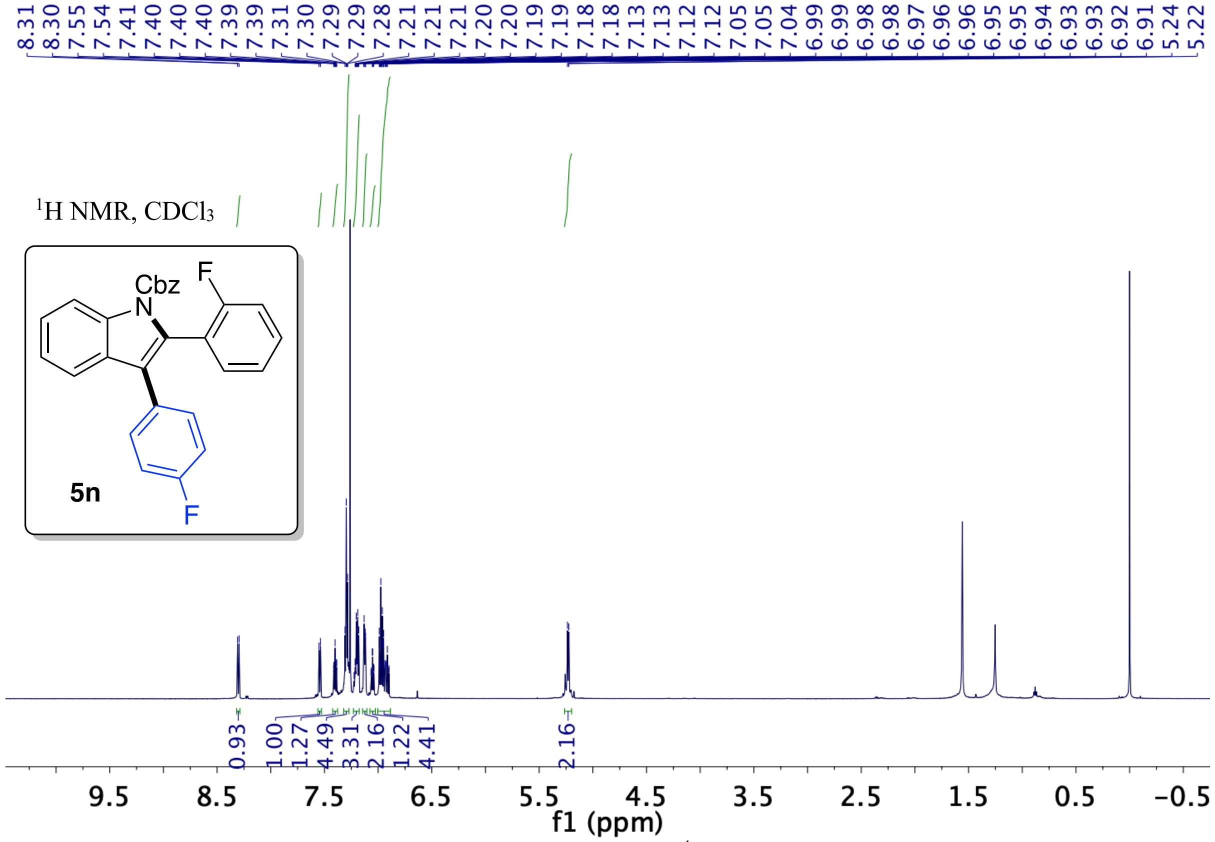

Supplementary Fig. 82. ^{1}H NMR Spectrum for **5n**

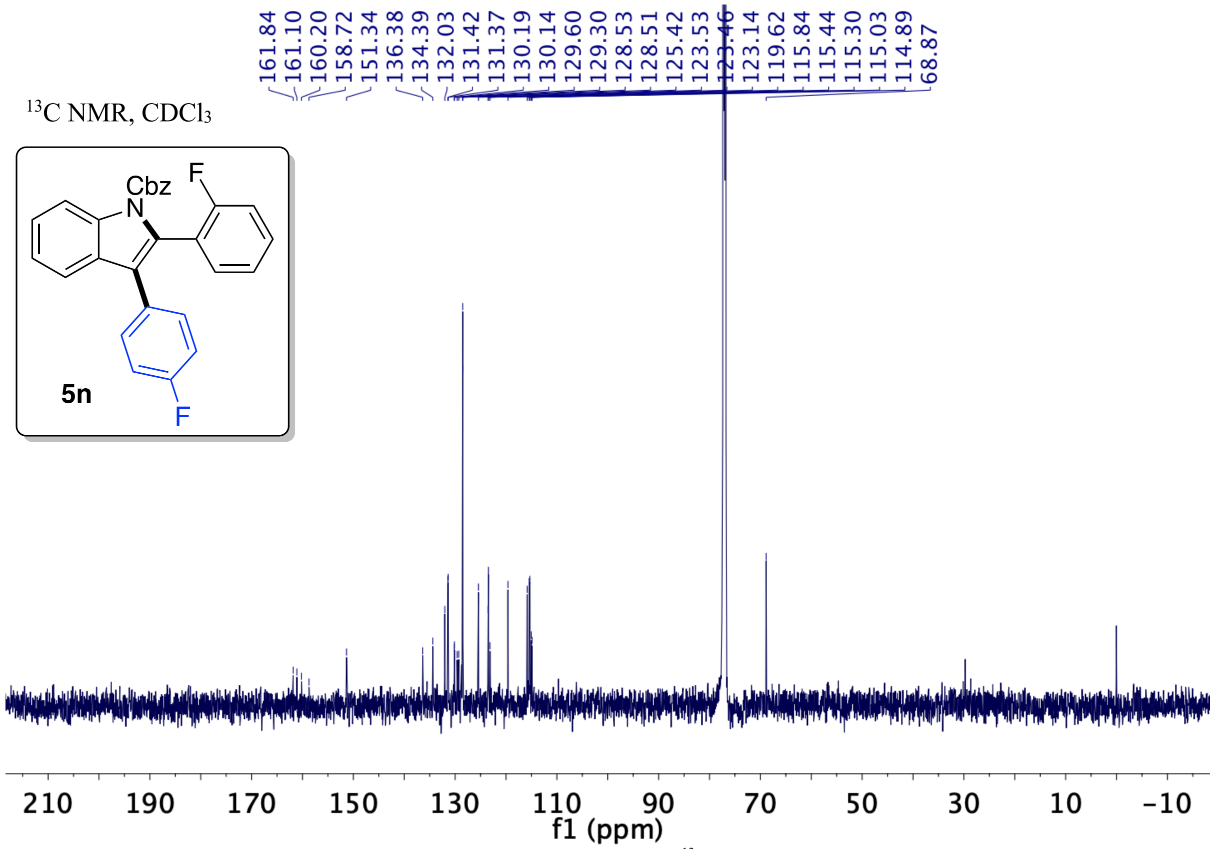

Supplementary Fig. 83. ^{13}C NMR Spectrum for **5n**

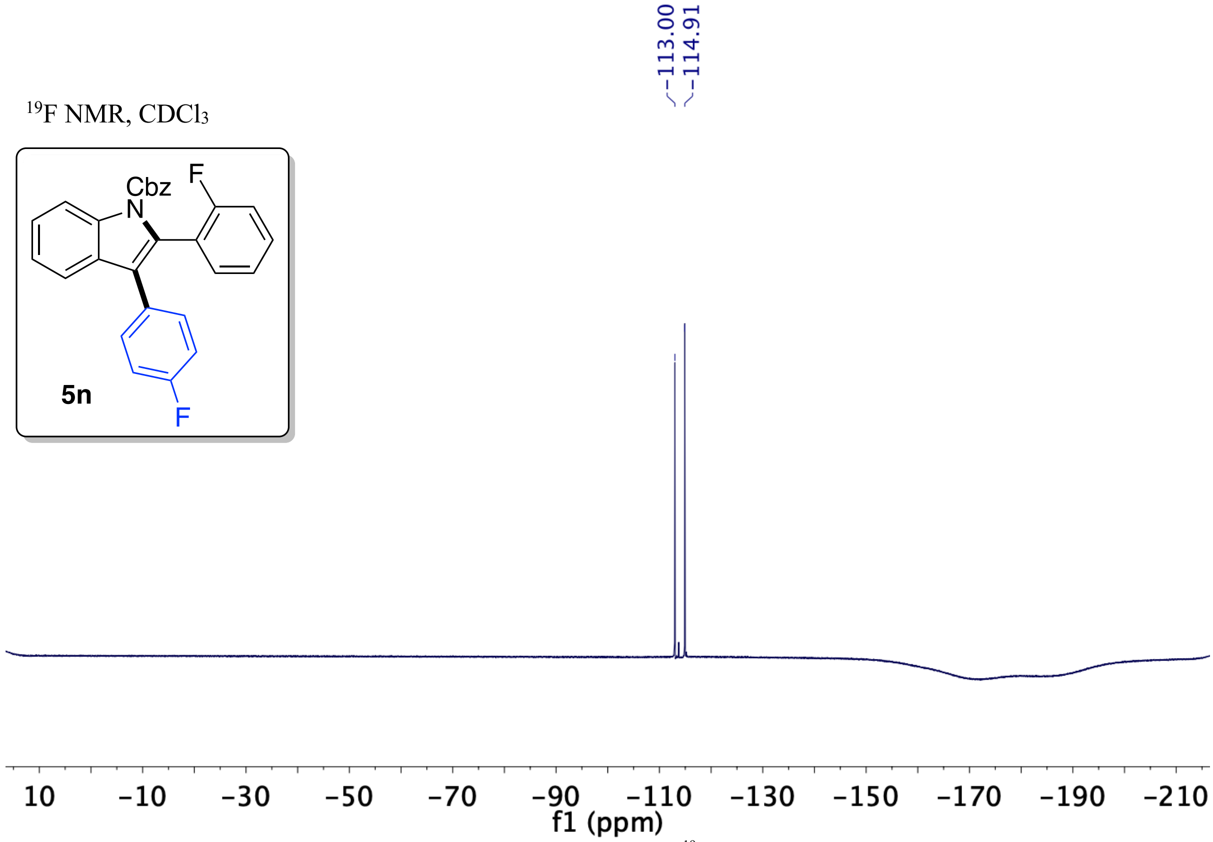

Supplementary Fig. 84. ^{19}F NMR Spectrum for **5n**

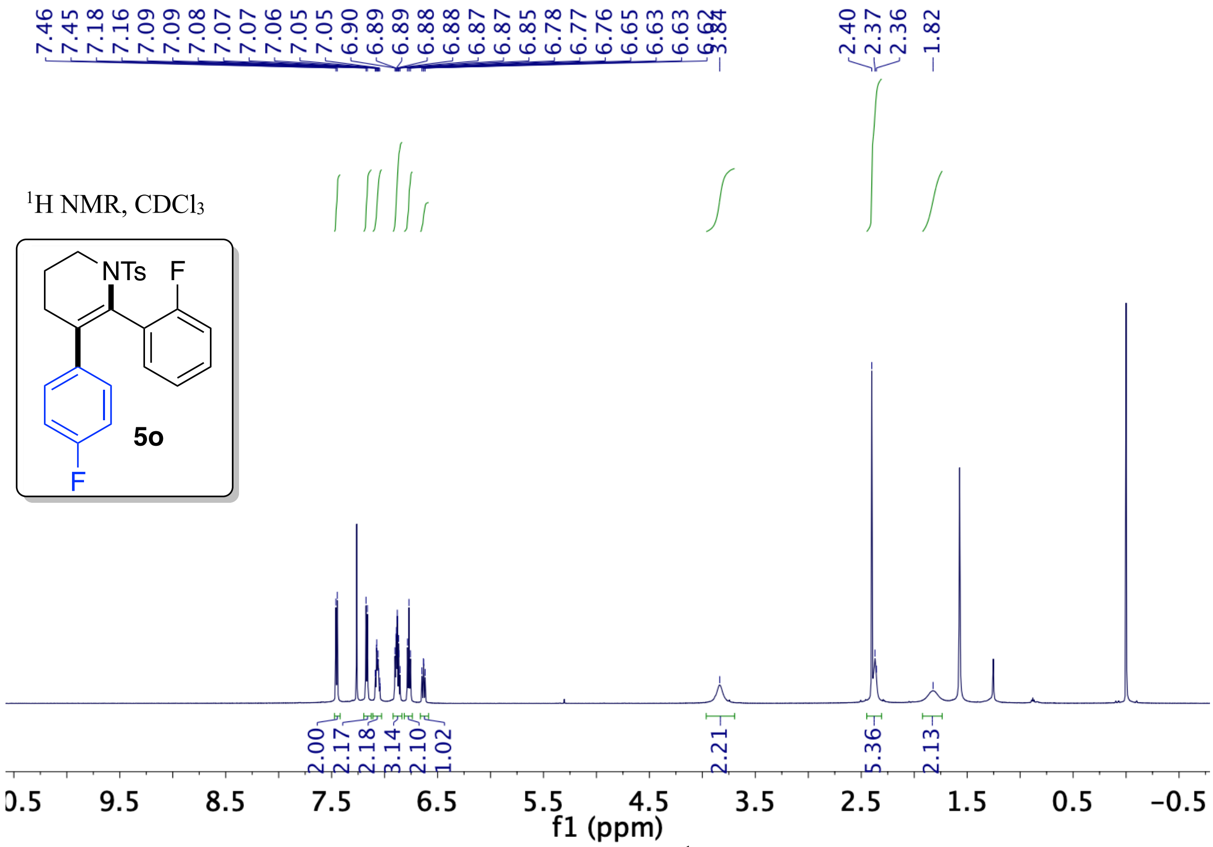

Supplementary Fig. 85. ^{1}H NMR Spectrum for **5o**

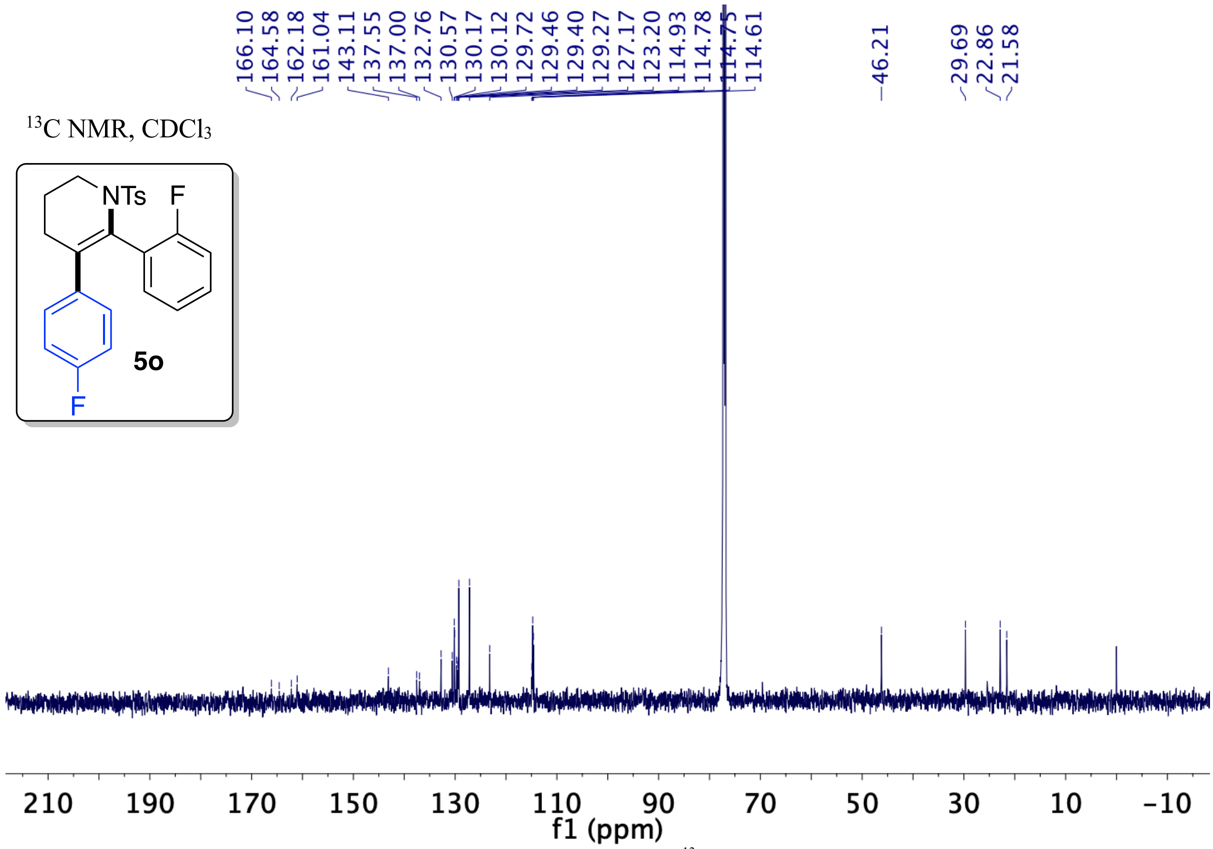

Supplementary Fig. 86. ^{13}C NMR Spectrum for **5o**

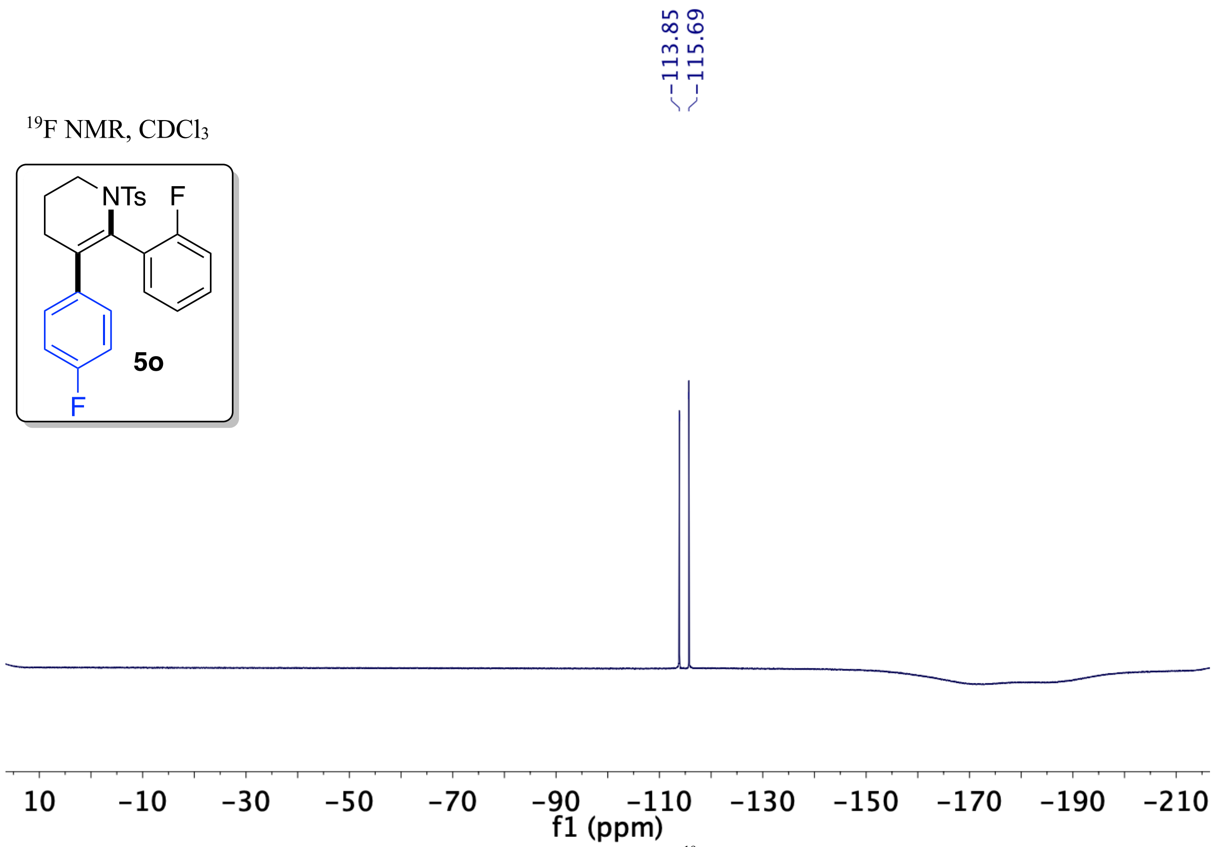

Supplementary Fig. 87. ^{19}F NMR Spectrum for **5o**

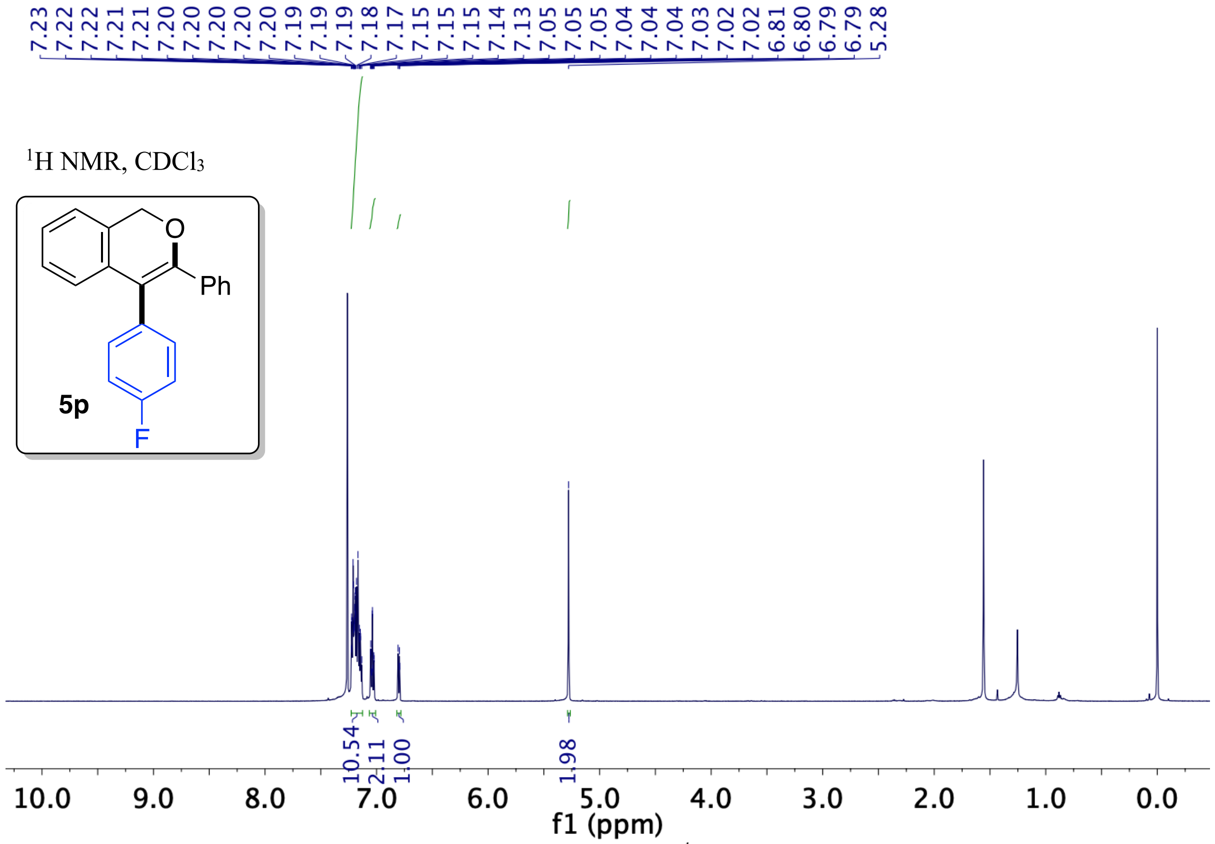

Supplementary Fig. 88. ^{1}H NMR Spectrum for **5p**

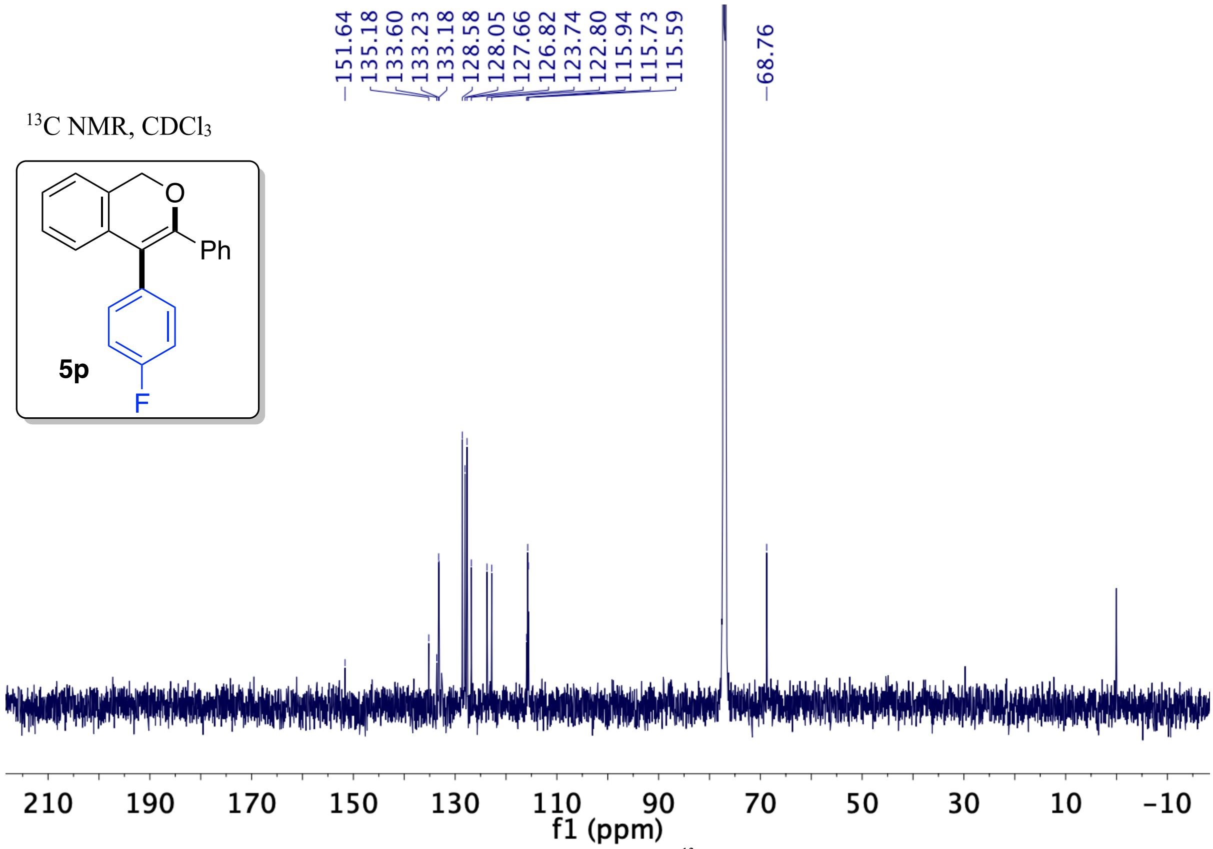

Supplementary Fig. 89. ^{13}C NMR Spectrum for **5p**

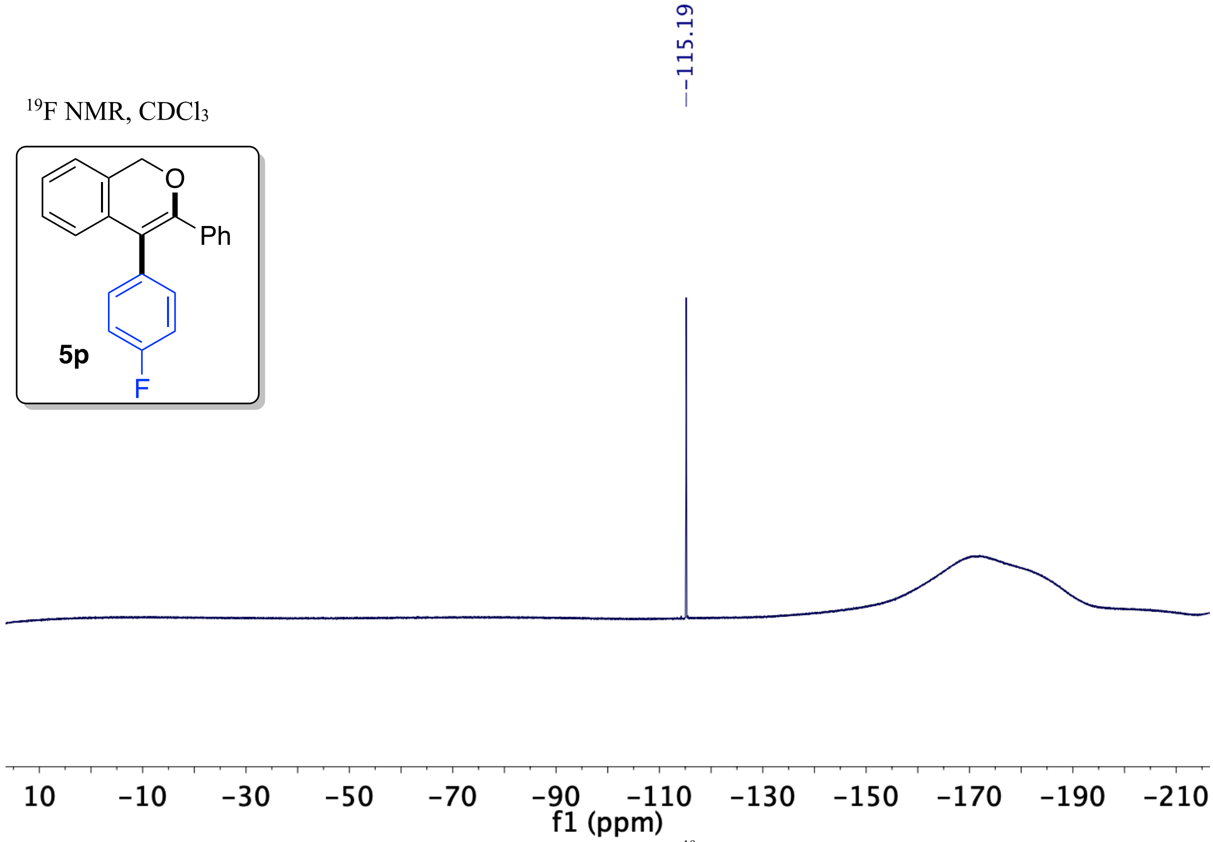

Supplementary Fig. 90. ^{19}F NMR Spectrum for **5p**

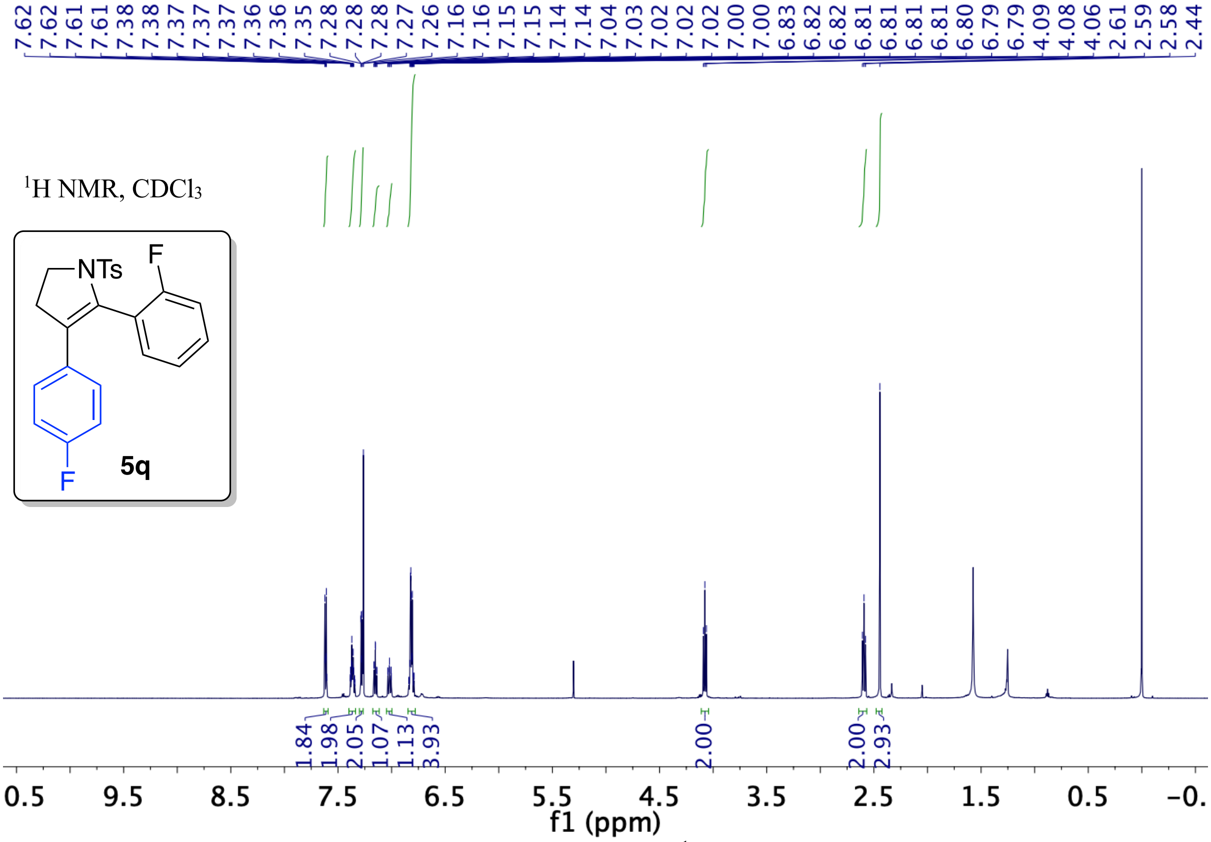

Supplementary Fig. 91. ^{1}H NMR Spectrum for **5q**

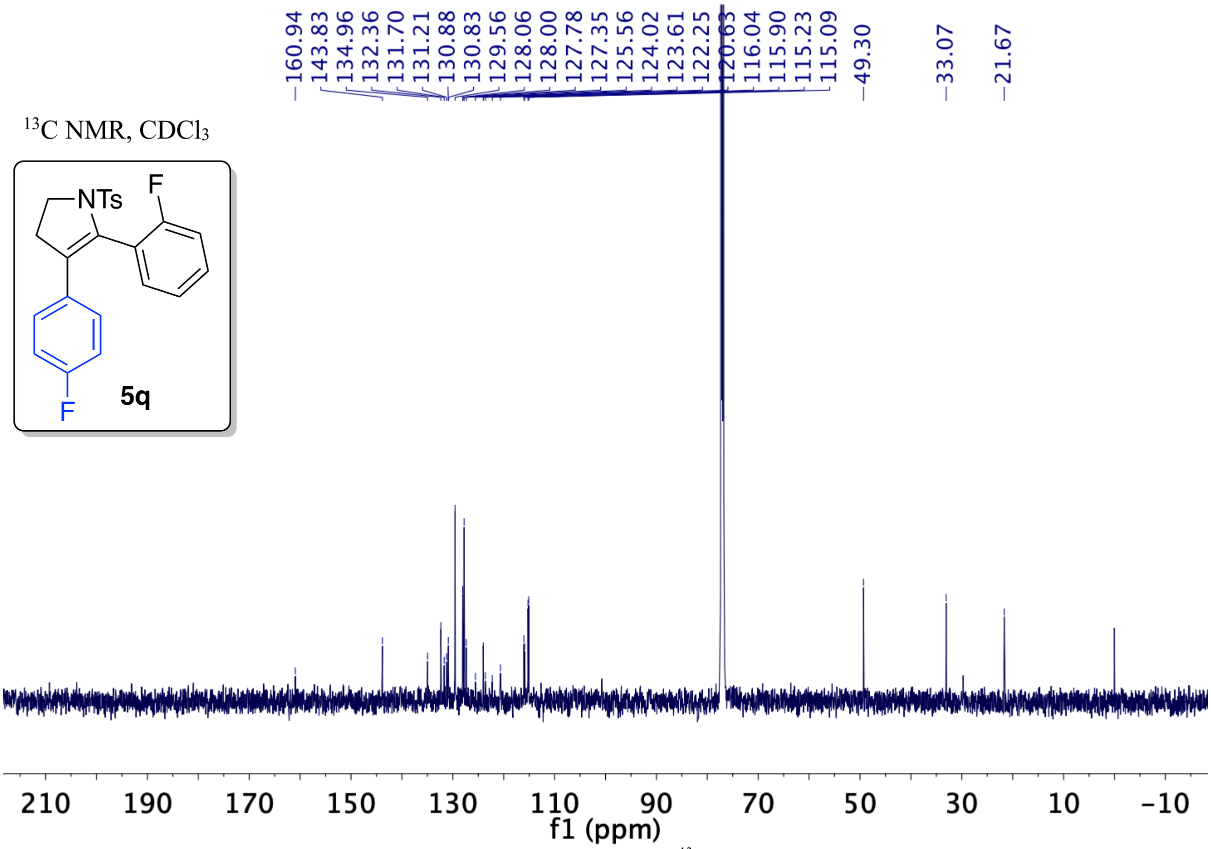

Supplementary Fig. 92. ^{13}C NMR Spectrum for **5q**

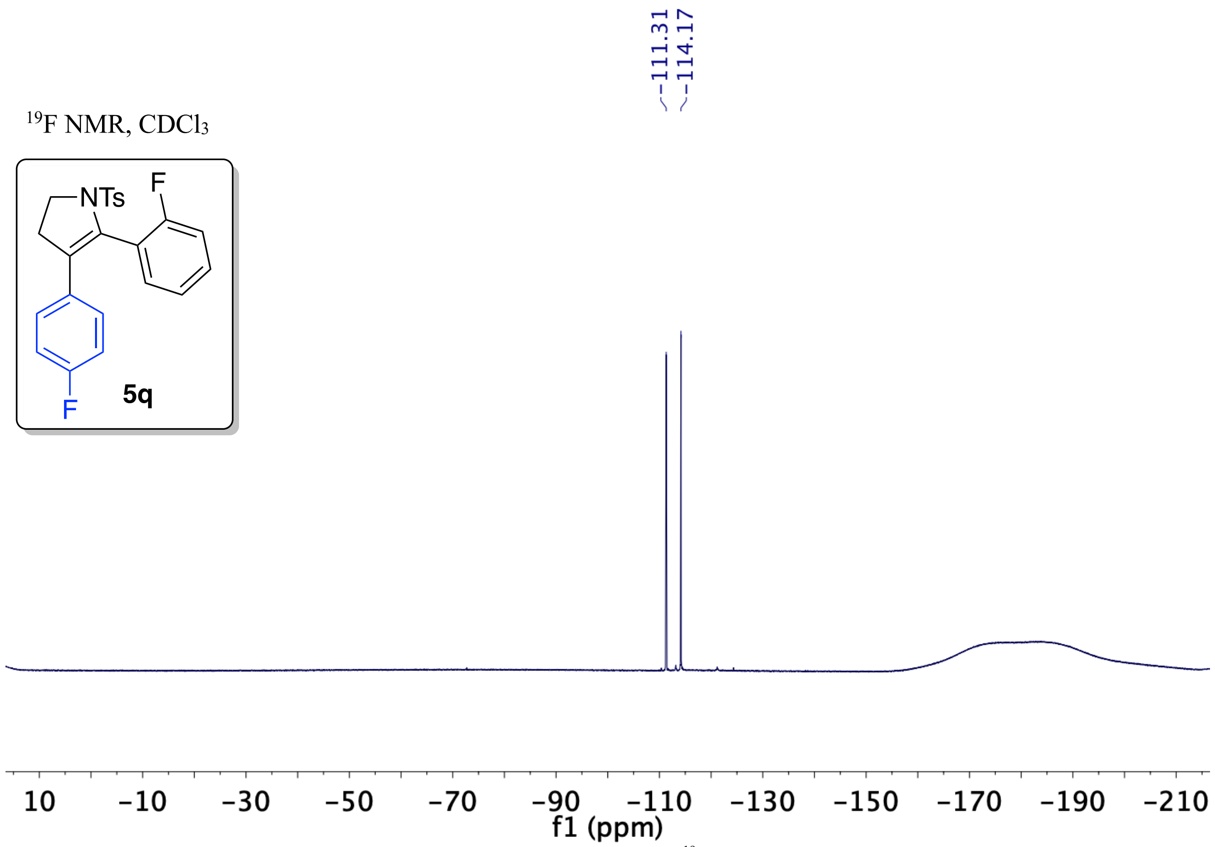

Supplementary Fig. 93. ^{19}F NMR Spectrum for **5q**
